# Assessing and projecting the global impacts of Alzheimer’s disease

**DOI:** 10.3389/fpubh.2024.1453489

**Published:** 2025-01-15

**Authors:** Nanlong Zhang, Shuren Chai, Jixing Wang

**Affiliations:** ^1^Department of Emergency, Ningbo Traditional Chinese Medicine Hospital, Ningbo, China; ^2^Department of Internal Medicine-Neurology, Ningbo Traditional Chinese Medicine Hospital, Ningbo, China

**Keywords:** Alzheimer’s disease, incidence, mortality, disability-adjusted life years, projection

## Abstract

**Background:**

This study aims to assess the global burden of Alzheimer’s disease (AD) from 1990 to 2030, with a focus on incidence, mortality, and disability-adjusted life years (DALY).

**Methods:**

Data on the incidence rates, DALY rates, and death rates of AD across various geographic populations from 1990 to 2021 were obtained from the Global Burden of Disease (GBD) 2021 study. Generalized Additive Models (GAMs) were employed to forecast the disease burden from 2022 to 2030.

**Results:**

The projected global burden of Alzheimer’s disease from 2022 to 2030 indicates a decrease in DALYs, with an Estimated Annual Percentage Change (EAPC) of −1.44 (95% CI: −1.45, −1.42). Similarly, death rates and incidence rates also show a decline, with EAPCs of −1.80 (95% CI: −1.83, −1.77) and −1.27 (95% CI: −1.29, −1.26) respectively. Gender-specific analysis reveals that the projected global incidence EAPC from 2022 to 2030 is estimated at −1.73 (95% CI: −1.75, −1.70) for males and −1.03 (95% CI: −1.04, −1.02) for females. Regionally, Andean Latin America and the Caribbean exhibit the highest positive EAPCs for DALYs at 0.94 (95% CI: 0.93, 0.94) and 0.59 (95% CI: 0.59, 0.60) respectively, while Eastern Europe shows the lowest EAPC at −16.31 (95% CI: −18.60, −13.95). Country-specific projections highlight Cyprus and Serbia with the highest positive EAPCs for DALYs at 12.55 (95% CI: 11.21, 13.91) and 9.6416 (95% CI: 8.86, 10.4333) respectively. On the other hand, Bahrain and Armenia exhibit significant negative EAPCs at −87.28 (95% CI: −94.66, −69.70) and −85.41 (95% CI: −92.80, −70.41). An analysis based on the Socio-Demographic Index (SDI) reveals that regions with higher SDI values have greater burdens of AD, with countries having SDI ≥ 0.8 showing significantly higher age-standardized Incidence Rates (ASIR), age-standardized Death Rates (ASDR), and age-standardized DALY rates compared to those with SDI < 0.8.

**Conclusion:**

From 1990 to 2030, global burden of AD is projected to decrease, with significant gender and regional disparities. Regions with higher SDI show higher disease burdens, underscoring the necessity for targeted interventions and customized public health strategies to effectively address AD in varied socio-economic settings.

## 1 Introduction

Alzheimer’s disease (AD) is a significant public health challenge, impacting more than 50 million individuals worldwide, a number projected to triple by 2050 due to an aging population (1). This progressive neurodegenerative condition is characterized by cognitive decline, memory loss, and diminished functional abilities, leading to complete reliance on caregivers. The economic impact of Alzheimer’s disease is substantial (2), with global costs estimated to surpass $1 trillion annually. In addition to financial implications, the disease also imposes significant psychological and social burdens on patients, families, and caregivers, resulting in widespread societal consequences (3). The rising prevalence and considerable burden of Alzheimer’s disease highlight the critical need for improved understanding and effective interventions (4).

The etiology of Alzheimer’s disease is influenced by a complex interaction of genetic, environmental, and lifestyle factors (5). Major risk factors include older age, genetic predispositions such as the APOE ε4 allele (6), and lifestyle choices like smoking, excessive alcohol consumption, and poor diet (7). Chronic conditions such as hypertension, diabetes, and obesity also contribute to the risk (8). Environmental factors, such as exposure to neurotoxins and air pollution, are significant as well. While progress has been made in understanding these risk factors, there are still gaps in our knowledge regarding the disease’s epidemiology and its impact on different socio-demographic groups and regions. It is crucial to have up-to-date and comprehensive global data on the burden of Alzheimer’s disease to inform the development of effective public health strategies and interventions (9).

This study utilizes data from the Global Burden of Disease (GBD) 2021 to provide a detailed analysis of the burden of AD from 1990 to 2021, with projections extending to 2030. By examining age-standardized rates (ASR) and estimated annual percentage changes (EAPC) for disability-adjusted life years (DALYs), this research offers a thorough overview of global trends. The study employs Generalized Additive Models (GAMs) to predict future trends and investigates the correlation between the Socio-Demographic Index (SDI) and the burden of AD. The findings reveal significant regional disparities, highlighting areas where the burden is most severe and where public health interventions are most urgently needed. This study underscores the necessity for targeted policies and preventive measures to mitigate the escalating burden of AD on a global scale.

## 2 Method

### 2.1 Data source

To comprehensively assess the burden of Alzheimer’s disease from 1990 to 2021, data was sourced from the Global Health Data Exchange (GHDx) platform’s GBD tool, covering 204 countries and territories. The GHDx serves as a vast repository of health-related information, incorporating data from surveys, censuses, and vital statistics. The 2021 GBD study integrated all available epidemiological data, implemented updated standard operating procedures, and conducted a thorough evaluation of health loss. This study examined 371 diseases and injuries, as well as 87 risk factors, across the 204 countries and territories. The GBD 2021 study is widely regarded for its integration of high-quality and representative data derived from multiple sources, including large-scale surveys, censuses, and vital registration systems. Each dataset underwent rigorous validation and standardization processes, including systematic quality assessments to ensure completeness, consistency, and reliability. Alzheimer’s disease data for this study was retrieved from the GBD online database, segmented by gender (male, female, combined), and standardized for various age groups through age-standardization (10). This study is based on a publicly available database and does not require ethical approval.

The Alzheimer’s disease-specific data in the GBD 2021 study incorporated both prevalence and incidence data. Incidence data, newly added in GBD 2021, were sourced from 81 data points across 60 locations. This enhancement was supported by excess mortality rate data derived from cohort studies comparing death risks in individuals with and without dementia, providing a robust basis for estimating incidence and prevalence trends over time. To minimize undue influence, USA claims data were excluded from the dementia modeling due to their disproportionate weight on global prevalence and incidence patterns relative to other input data. Adjustments were made using matched datasets to ensure adherence to the reference case definition.

The GBD study incorporates advanced modeling tools, such as the Cause of Death Ensemble model (CODEm) and DisMod-MR 2.1, a Bayesian meta-regression tool, to address data gaps and maintain internal consistency across locations and time periods. These models use covariate data, geographical patterns, and other relevant inputs to produce robust estimates of mortality and disease burden even in regions with limited direct data availability (11).

The GBD’s SDI is a comprehensive measure that takes into account per capita income, education level, and fertility rate. This index has a range of 0 to 1, where higher scores reflect regions with higher per capita income, increased education levels, and lower fertility rates. Countries were classified into five development level groups—high (≥0.80), upper-middle (0.70–0.79), middle (0.60–0.69), lower-middle (0.50–0.59), and low (<0.50)—based on their SDI scores. As the data utilized in this research is publicly available, ethical approval was considered unnecessary (12).

The ICD codes for Alzheimer’s disease in the ninth revision (ICD-9) range from 290.0 to 290.9, encompassing conditions like senile dementia (290.0), presenile dementia (290.1), and unspecified senile dementia (290.9). In the tenth revision (ICD-10), the codes span from G30.0 to G30.9, covering Alzheimer’s disease with early onset (G30.0), Alzheimer’s disease with late onset (G30.1), other Alzheimer’s disease (G30.8), and Alzheimer’s disease, unspecified (G30.9). These codes provide a comprehensive framework for diagnosing Alzheimer’s disease across all age groups (13, 14).

### 2.2 Statistical analysis

This study assesses the burden of Alzheimer’s disease using ASRs, incidence rates, death rates, and DALY rates directly obtained from the GBD 2021 study for the years 1990 to 2021. These metrics were generated using GBD’s standardized analytical framework and were not calculated independently. The use of this framework ensures global comparability of disease metrics. All projections from 2022 to 2030 were based on Generalized Additive Models (GAMs) using these pre-computed metrics. The analysis includes ASR for incidence, mortality, and DALYs across all age groups to determine the global health impact.

The ASR was calculated by taking a weighted average of the specific rates for each age group, with the standard population used as the weighting factor (15). The formula for ASR is as follows:


$$ASR=\overset{n}{\underset{i=1}{\sum }}w_{i}r_{i}$$

The weight for each age group ( $w_{i}$ ) represents the standard population weight, while $r_{i}$ denotes the rate (incidence, death, or DALY) for that specific age group (16). The calculation of the standard population weight ( $w_{i}$ ) for each age group follows a specific formula.


$$w_{i}=\frac{P_{i}}{\sum_{i=1}^{n}P_{i}}$$

where $P_{i}$ is the population of the $i^{th}$ age group in the standard population.

To assess the impact of Alzheimer’s disease on quality of life, we combined incidence data with disability weights to calculate DALYs, providing a comprehensive measure of the disease burden. Utilizing GAMs, we examined the non-linear correlation between time and age-standardized rates of Alzheimer’s disease. This approach enabled us to flexibly model disease trends over time, with smooth functions adjusting to variations in predictor variables like calendar year and median age (17). The model can be represented as:


$$y=\beta_{0}+s\left(x1\right)+s\left(x2\right)\ldots +s\left(xn\right)$$

The predicted age-standardized rates (y) are calculated using the intercept ( $\beta_{0}$ ), a smooth function ( $s\left(x\right)$ ) representing the non-linear relationship with predictor variables, and the residual term ( ϵ ) capturing unexplained variation (16).

We calculated the EAPC for the periods 1990–2021 and 2022–2030 to assess trends over time in disease burden. EAPC offers valuable information on the rate of change, where positive values signify an increase and negative values indicate a decrease (17). The formula for EAPC is:


$$\mathrm{EAPC}=\left({\left(\frac{{ASR}_{\mathrm{end}}}{{ASR}_{\mathrm{start}}}\right)}^{\frac{1}{t}}-1\right)\times 100$$

where ${ASR}_{\mathrm{end}}\;\mathrm{and}\;{ASR}_{\mathrm{start}}$ represent the age-standardized rates at the end and start of the period, respectively, and t is the number of years in the period (18).

Intergroup comparisons were conducted using the Mann–Whitney U test to analyze variations in Alzheimer’s disease burden across the five continents. This non-parametric test is appropriate for comparing independent samples and determining if there are notable differences between groups (19). The formula for the Mann-Whitney U test is:


$$U=n_{1}n_{2}+\frac{n_{1}\left(n_{1}+1\right)}{2}-R_{1}$$

where $n_{1}$ and $n_{2}$ are the sample sizes of the two groups, and $R_{1}$ is the sum of the ranks for the first group (20).

The study investigated the association between the SDI and Alzheimer’s disease burden in various regions. Linear regression and Pearson correlation were utilized to evaluate these connections, while considering potential confounders like gender and regional economic status. SDI, a composite index incorporating per capita income, education level, and fertility rate, ranges from 0 to 1, with higher values indicating better socio-demographic conditions. This analysis shed light on the relationship between socio-economic factors and Alzheimer’s disease prevalence, offering valuable insights for public health interventions.

## 3 Results

### 3.1 Projected global burden of Alzheimer’s disease, 2022–2030

**Figure 1:**
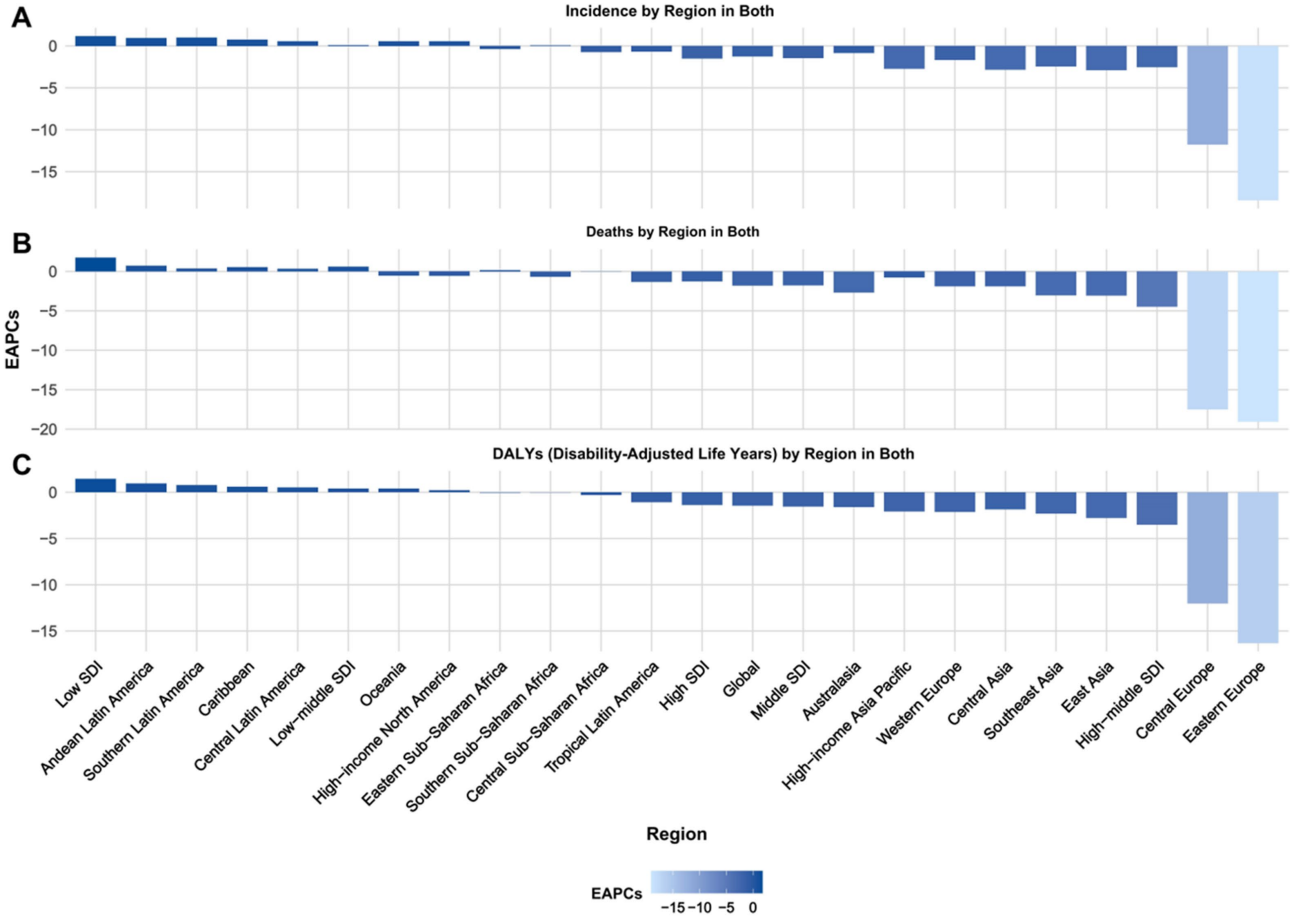
Projected EAPC of global burden of Alzheimer’s disease from 2022 to 2030, by locations. **(A)** ASIR, **(B)** ASDR, and **(C)** age-standardized DALY rate. DALY = disability adjusted life-year. ASIR = age standardized incidence rate. ASDR = age standardized death rate. ASRs = age standardized rates.

The EAPC for DALYs shows a decrease of −1.56 (95% CI: −1.63, −1.48) from 1990 to 2021 and a projected EAPC of −1.44 (95% CI: −1.45, −1.42) from 2022 to 2030. Similarly, the EAPC for death rates decreased to −1.53 (95% CI: −1.64, −1.43) from 1990 to 2021 and is expected to further decrease to −1.80 (95% CI: −1.82, −1.77) from 2022 to 2030. The EAPC for the incidence rate decreased to −1.58 (95% CI: −1.65, −1.51) from 1990 to 2021 and is projected to decrease to −1.27 (95% CI: −1.29, −1.26) from 2022 to 2030 (Table 1; Figure 1).

**Table 1:** The projected EAPC of Alzheimer’s disease from 1990 to 2021 and from 2022 to 2030 in different regions.

Location	DALYs (Disability-Adjusted Life Years)		Deaths		Incidence	
	1990–2021	2022–2030	1990–2021	2022–2030	1990–2021	2022–2030
Global	−1.5552 (−1.6338, −1.4765)	−1.4375 (−1.4548, −1.4201)	−1.5340 (−1.6420, −1.4259)	−1.7982 (−1.8254, −1.7711)	−1.5811 (−1.6515, −1.5106)	−1.2718 (−1.2854, −1.2583)
High SDI	−1.5359 (−1.6362, −1.4355)	−1.3768 (−1.3927, −1.3609)	−1.2994 (−1.4293, −1.1693)	−1.2757 (−1.2893, −1.2620)	−1.7039 (−1.7918, −1.6159)	−1.5120 (−1.5312, −1.4928)
High-middle SDI	−1.9608 (−2.0728, −1.8487)	−3.5088 (−3.6123, −3.4053)	−1.9799 (−2.1084, −1.8513)	−4.4899 (−4.6594, −4.3201)	−1.8240 (−1.9353, −1.7126)	−2.5446 (−2.5990, −2.4902)
Middle SDI	−1.8764 (−1.9214, −1.8313)	−1.5541 (−1.5744, −1.5338)	−1.9140 (−1.9747, −1.8533)	−1.7881 (−1.8150, −1.7613)	−1.7509 (−1.7978, −1.7040)	−1.4431 (−1.4606, −1.4256)
Low-middle SDI	−0.8225 (−1.0177, −0.6270)	0.3827 (0.3815, 0.3840)	−0.7075 (−0.9462, −0.4682)	0.6081 (0.6050, 0.6112)	−1.1799 (−1.3746, −0.9848)	0.0898 (0.0897, 0.0899)
Low SDI	−0.9409 (−1.2267, −0.6543)	1.4387 (1.4213, 1.4560)	−0.7516 (−1.0655, −0.4368)	1.7575 (1.7315, 1.7834)	−1.3055 (−1.5642, −1.0460)	1.1660 (1.1546, 1.1774)
Andean Latin America	−0.8578 (−1.0347, −0.6807)	0.9371 (0.9298, 0.9445)	−0.7216 (−0.8828, −0.5601)	0.7193 (0.7150, 0.7237)	−0.8531 (−1.0585, −0.6473)	0.9506 (0.9430, 0.9582)
Australasia	−1.9689 (−2.0724, −1.8653)	−1.6019 (−1.6234, −1.5804)	−1.5846 (−1.6763, −1.4928)	−2.6780 (−2.7383, −2.6178)	−2.4095 (−2.5272, −2.2916)	−0.8525 (−0.8586, −0.8464)
Caribbean	−2.2176 (−2.6458, −1.7875)	0.5931 (0.5902, 0.5961)	−2.2061 (−2.6876, −1.7222)	0.5578 (0.5552, 0.5605)	−2.2986 (−2.7170, −1.8784)	0.7553 (0.7505, 0.7600)
Central Asia	0.1902 (−0.0485, 0.4295)	−1.8372 (−1.8655, −1.8088)	0.0483 (−0.2683, 0.3659)	−1.8902 (−1.9202, −1.8602)	0.2948 (0.0196, 0.5708)	−2.8444 (−2.9123, −2.7764)
Central Europe	−2.0893 (−2.4364, −1.7409)	−12.0323 (−13.2652, −10.7819)	−1.9335 (−2.3407, −1.5245)	−17.4959 (−20.1515, −14.7520)	−2.1841 (−2.5428, −1.8241)	−11.7452 (−12.9190, −10.5555)
Central Latin America	−1.6558 (−1.8630, −1.4481)	0.5100 (0.5078, 0.5122)	−1.6311 (−1.8649, −1.3967)	0.3524 (0.3513, 0.3534)	−1.7699 (−1.9853, −1.5541)	0.5536 (0.5510, 0.5562)
Central Sub-Saharan Africa	−1.7051 (−1.9678, −1.4417)	−0.2830 (−0.2837, −0.2823)	−1.3490 (−1.6892, −1.0077)	0.0273 (0.0273, 0.0273)	−2.0907 (−2.3664, −1.8142)	−0.7369 (−0.7414, −0.7323)
East Asia	−3.7791 (−3.9877, −3.5700)	−2.7709 (−2.8354, −2.7064)	−4.1028 (−4.3379, −3.8671)	−3.0900 (−3.1702, −3.0097)	−3.3151 (−3.5264, −3.1034)	−2.9029 (−2.9737, −2.8321)
Eastern Europe	−1.6335 (−2.1065, −1.1582)	−16.3071 (−18.6030, −13.9464)	−1.4849 (−1.8916, −1.0766)	−19.0569 (−22.2295, −15.7549)	−1.7262 (−2.2657, −1.1837)	−18.4027 (−21.3524, −15.3424)
Eastern Sub-Saharan Africa	−1.3434 (−1.4894, −1.1971)	−0.0259 (−0.0259, −0.0259)	−1.2433 (−1.3987, −1.0877)	0.1719 (0.1717, 0.1722)	−1.6510 (−1.7887, −1.5132)	−0.3794 (−0.3806, −0.3782)
High-income Asia Pacific	−2.5378 (−2.7984, −2.2764)	−2.0629 (−2.0986, −2.0271)	−2.0830 (−2.4510, −1.7136)	−0.7750 (−0.7801, −0.7700)	−2.6393 (−2.8307, −2.4475)	−2.7236 (−2.7859, −2.6612)
High-income North America	−0.9496 (−1.0038, −0.8953)	0.2162 (0.2158, 0.2166)	−0.7462 (−0.8003, −0.6921)	−0.5813 (−0.5841, −0.5784)	−1.1648 (−1.2424, −1.0872)	0.5365 (0.5340, 0.5389)
Oceania	−2.9271 (−3.2021, −2.6512)	0.3937 (0.3924, 0.3951)	−3.0667 (−3.4052, −2.7271)	−0.5430 (−0.5455, −0.5405)	−2.9121 (−3.1914, −2.6320)	0.5631 (0.5604, 0.5657)
Southeast Asia	−0.5103 (−0.6261, −0.3944)	−2.2938 (−2.3379, −2.2496)	−0.3476 (−0.4816, −0.2134)	−3.0312 (−3.1084, −2.9540)	−0.7474 (−0.8733, −0.6213)	−2.4581 (−2.5088, −2.4073)
Southern Latin America	−1.6771 (−1.8942, −1.4595)	0.7651 (0.7602, 0.7700)	−1.5095 (−1.7360, −1.2825)	0.3688 (0.3677, 0.3700)	−1.8273 (−2.0434, −1.6108)	0.9995 (0.9911, 1.0079)
Southern Sub-Saharan Africa	1.2465 (0.8724, 1.6219)	0.0033 (0.0033, 0.0033)	1.5847 (1.1438, 2.0275)	−0.6755 (−0.6794, −0.6717)	1.0432 (0.7072, 1.3803)	0.0592 (0.0592, 0.0593)
Tropical Latin America	−2.2432 (−2.4935, −1.9922)	−1.0657 (−1.0753, −1.0562)	−2.2974 (−2.5892, −2.0048)	−1.3193 (−1.3339, −1.3047)	−2.2681 (−2.4946, −2.0411)	−0.6871 (−0.6910, −0.6831)
Western Europe	−1.7577 (−1.9674, −1.5475)	−2.1207 (−2.1585, −2.0829)	−1.5321 (−1.7887, −1.2747)	−1.8998 (−1.9301, −1.8695)	−1.9223 (−2.1272, −1.7169)	−1.6820 (−1.7058, −1.6582)

**Figure 2:**
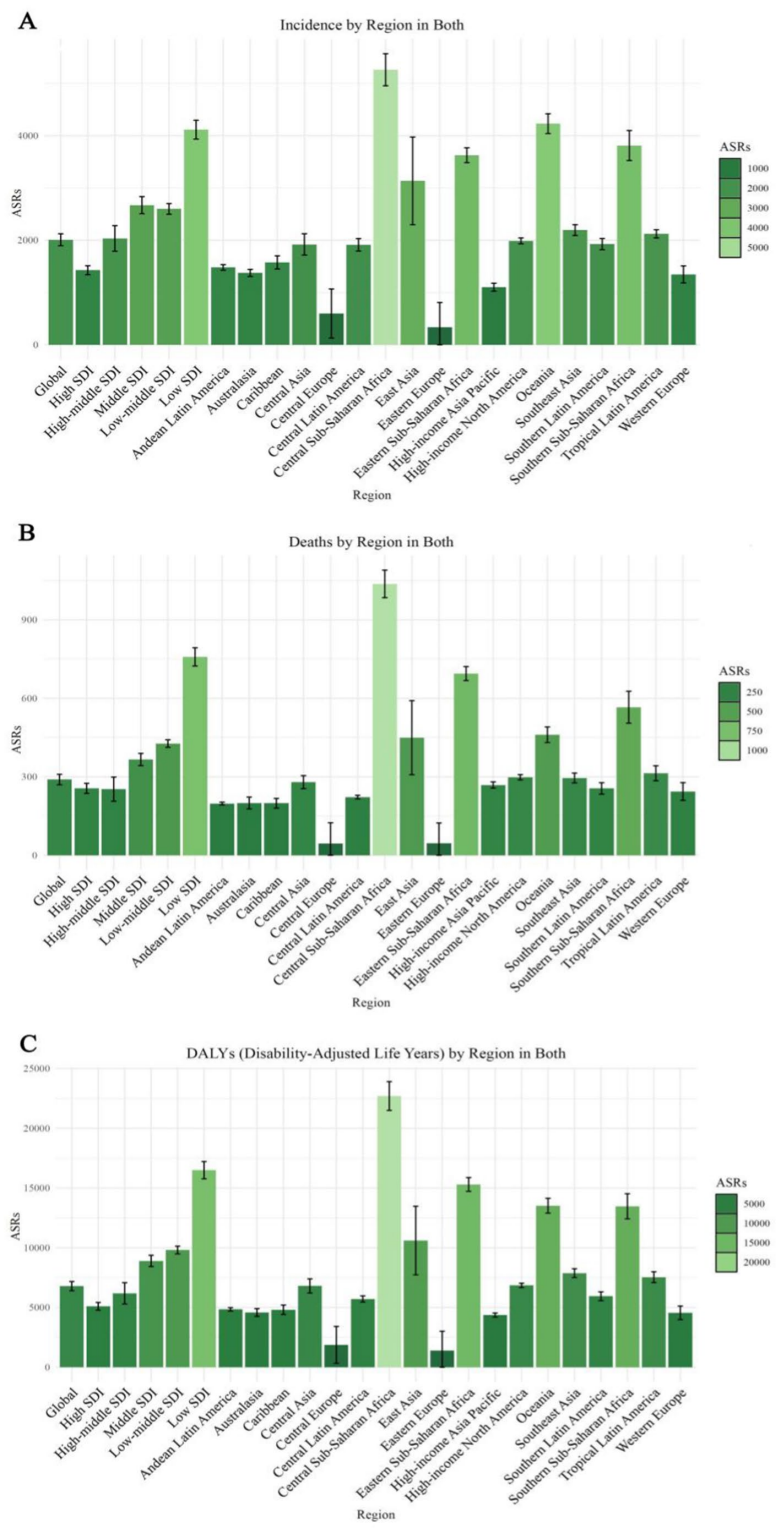
Projected global burden of age-standardized rates of Alzheimer’s disease in 2030, by locations. **(A)** ASIR, **(B)** ASDR, and **(C)** age-standardized DALY rate. DALY = disability adjusted life-year. ASIR = age standardized incidence rate. ASDR = age standardized death rate. ASRs = age standardized rates.

**Figure 3:**
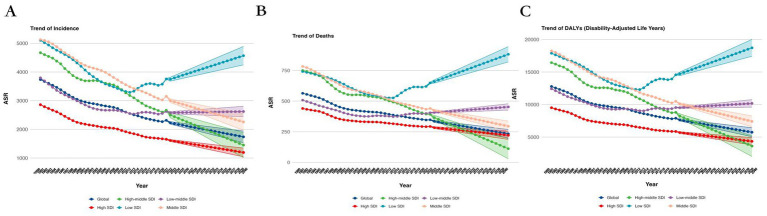
Projected Trends of global burden of age-standardized rates of Alzheimer’s disease from 1990 to 2030, by SDI regions. **(A)** ASIR, **(B)** ASDR, and **(C)** age-standardized DALY rate. DALY = disability adjusted life-year. ASIR = age standardized incidence rate. ASDR = age standardized death rate. ASRs = age standardized rates.

In 1990, the DALYs for ASR were 644.16 (95% UI: 640.90, 647.43), with a projected decrease to 299.92 (95% UI: 274.94, 324.91) by 2030. The death rate in 1990 was 14775.89 (95% UI: 14760.61, 14791.18) and is expected to decrease to 7210.92 (95% UI: 6730.65, 7691.19) by 2030. Similarly, the incidence rate in 1990 stood at 4483.76 (95% UI: 4475.36, 4492.18) and is projected to decrease to 2199.67 (95% UI: 2054.49, 2344.86) by 2030 (Supplementary Table 1, Figures 2, 3).

### 3.2 Projected global burden of Alzheimer’s disease by sex, 2022–2030

From 2022 to 2030, the global burden of Alzheimer’s disease demonstrates distinct trends in both EAPC and ASR. The death rates for both males and females show a decline in EAPC. The global EAPC for males is −2.28 (95% CI: −2.32, −2.23) and for females is −1.03 (95% CI: −1.04, −1.02). Similarly, the incidence rates for both sexes also exhibit a decrease, with the EAPC for males at −1.73 (95% CI: −1.75, −1.70) and for females at −1.03 (95% CI: −1.04, −1.02).

**Figure 4:**
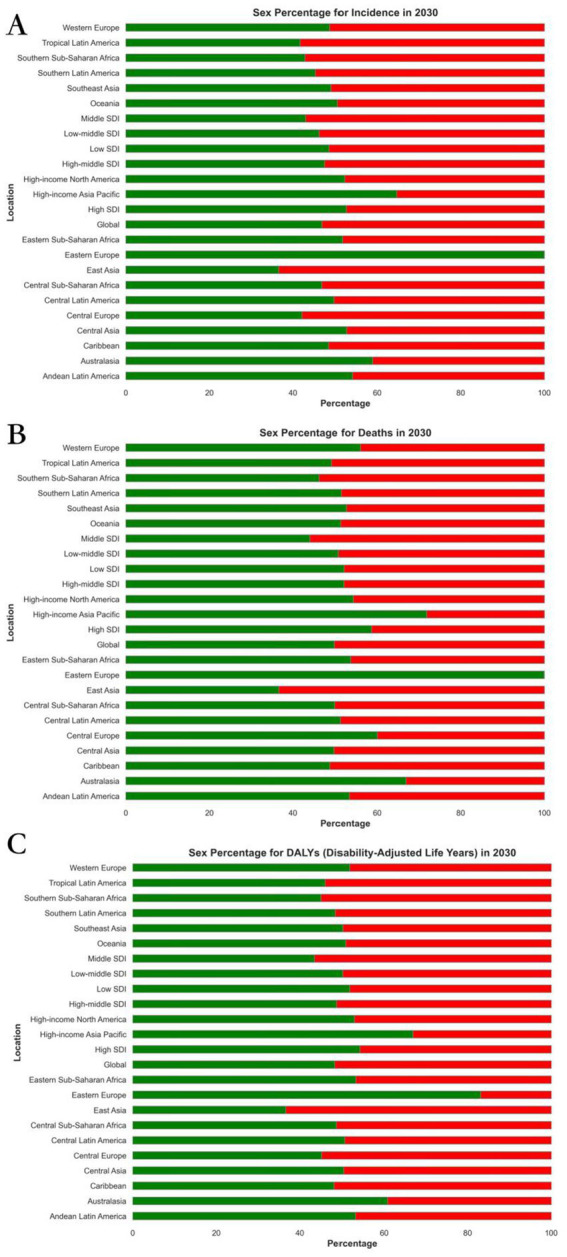
Projected global burden of rate percentages of Alzheimer’s disease in 2030, by sexes and locations. **(A)** ASIR, **(B)** ASDR, and **(C)** age-standardized DALY rate. DALY = disability adjusted life-year. ASIR = age standardized incidence rate. ASDR = age standardized death rate. ASRs = age standardized rates.

In 2030, the age-standardized death rate for males was 6786.66 (95% UI: 6401.84, 7171.47) and for females it was 6751.16 (95% UI: 6389.90, 7112.42). The age-standardized DALY rate for males was 289.55 (95% UI: 269.55, 309.55) and for females it was 298.85 (95% UI: 279.72, 317.97). Additionally, the age-standardized incidence rate for males in 2030 was 2009.00 (95% UI: 1895.67, 2122.33) and for females it was 1947.26 (95% UI: 1843.42, 2051.10). These figures can be further explored in Supplementary Tables 2, 3, and Figure 4.

### 3.3 Projection for the distribution of Alzheimer’s disease in different regions, 2022–2030

From 2022 to 2030, the projected global burden of Alzheimer’s disease displays notable regional disparities, as evidenced by the EAPC and ASR of DALYs.

Regions with the highest positive EAPC in DALYs due to Alzheimer’s disease are Andean Latin America, Southern Latin America, and the Caribbean. Andean Latin America has the highest EAPC at 0.94 (95% CI: 0.93, 0.94), followed by Southern Latin America at 0.77 (95% CI: 0.76, 0.77), and the Caribbean at 0.59 (95% CI: 0.59, 0.60). Central Latin America and Oceania also exhibit positive EAPCs of 0.51 (95% CI: 0.51, 0.51) and 0.39 (95% CI: 0.39, 0.39) respectively, indicating a rising trend in the burden of Alzheimer’s disease in these regions (Table 1; Supplementary Table 4; Figure 1).

Regions like Eastern Europe, Central Europe, and East Asia demonstrate notable negative EAPCs. Specifically, Eastern Europe shows the lowest EAPC at −16.31 (95% CI: −18.60, −13.95), followed by Central Europe at −12.03 (95% CI: −13.26, −10.78), and East Asia at −2.77 (95% CI: −2.83, −2.70). Additionally, Southeast Asia and Western Europe also exhibit negative EAPCs of −2.29 (95% CI: −2.34, −2.25) and − 2.12 (95% CI: −2.16, −2.08) respectively, indicating a downward trend in these regions (Table 1, Supplementary Table 4, and Figure 1).

In terms of ASR for DALYs, Central Sub-Saharan Africa is expected to have the highest ASR at 22703.53 (95% UI: 21500.55, 23906.51), followed by Eastern Sub-Saharan Africa at 15299.70 (95% UI: 14724.70, 15874.71), and Oceania at 13522.19 (95% UI: 12900.14, 14144.24). Southern Sub-Saharan Africa also exhibits a high ASR of 13477.61 (95% UI: 12428.99, 14526.24), highlighting a considerable burden of Alzheimer’s disease in these regions (Supplementary Tables 2, 5; Figure 2).

Regions with the lowest ASR for DALYs include Eastern Europe, Central Europe, and High-income Asia Pacific. Eastern Europe has the lowest ASR at 1404.84 (95% Uncertainty Interval [UI]: 0.05, 3014.43), followed by Central Europe at 1872.69 (95% UI: 330.75, 3414.64), and High-income Asia Pacific at 4365.22 (95% UI: 4190.62, 4539.83). Additionally, Western Europe and Australasia exhibit low ASRs of 4555.77 (95% UI: 3986.67, 5124.87) and 4581.69 (95% UI: 4256.90, 4906.48), respectively (Supplementary Tables 2, 5; Figure 2).

These projections highlight the disparities in the burden of Alzheimer’s disease across different regions, with some areas experiencing significant increases while others show declining trends. The regions with the highest ASR values indicate a continued high burden, while those with the lowest ASR values suggest better management and control of the disease.

### 3.4 Projection for the distribution of Alzheimer’s disease in different countries, 2022–2030

From 2022 to 2030, the projected global burden of Alzheimer’s disease shows notable disparities among various countries, as evidenced by the EAPC and ASR of DALYs. These discrepancies highlight the varying degrees of disease management and control measures implemented on a global scale (see Supplementary Tables 6, 7).

Countries with the highest positive EAPC in DALYs due to Alzheimer’s disease include Cyprus, Serbia, and Montenegro. Cyprus leads with an EAPC of 12.55 (95% CI: 11.21, 13.91), followed by Serbia at 9.64 (95% CI: 8.85, 10.43), and Montenegro at 5.16 (95% CI: 4.93, 5.38). Additionally, Andorra and Cuba also exhibit positive EAPCs of 5.11 (95% CI: 4.89, 5.33) and 4.95 (95% CI: 4.74, 5.15), respectively, indicating an increasing trend in the burden of Alzheimer’s disease in these regions. Conversely, countries such as Bahrain, Armenia, and Qatar show substantial negative EAPCs. Bahrain has the lowest EAPC at −87.28 (95% CI: −94.66, −69.69), followed by Armenia at −85.40 (95% CI: −92.80, −70.41), and Qatar at −85.39 (95% CI: −93.13, −68.92). Romania and Guatemala also demonstrate negative EAPCs of −84.49 (95% CI: −92.08, −69.66) and − 78.37 (95% CI: −90.61, −50.17), respectively, indicating a declining trend in these regions (Supplementary Tables 6, 8; Supplementary Figure 1).

In terms of ASR for DALYs, Cyprus is expected to have the highest ASR at 296472.95 (95% UI: 127878.51, 465067.40), followed by North Macedonia at 260543.83 (95% UI: 202167.92, 318919.74), and the United Arab Emirates at 82789.36 (95% UI: 79632.65, 85946.07). Eswatini and the Central African Republic also exhibit high ASRs of 52861.67 (95% UI: 49104.71, 56618.63) and 52156.45 (95% UI: 47941.08, 56371.83) respectively, indicating a notable burden of Alzheimer’s disease in these nations. On the other hand, countries with the lowest ASRs for DALYs include Armenia, Bulgaria, and Romania. Armenia records the lowest ASR at 0.05 (95% UI: 0.05, 1653.38), followed by Bulgaria at 0.05 (95% UI: 0.05, 54018.61), and Romania at 0.05 (95% UI: 0.05, 2897.19). Guatemala and Bahrain also display low ASRs of 0.05 (95% UI: 0.05, 4016.29) and 0.05 (95% UI: 0.05, 12522.80) respectively (Supplementary Tables 7, 9; Supplementary Figure 1).

### 3.5 Projection for the distribution and of correlation analysis of Alzheimer’s disease in different levels of SDI and different continents, 2022–2030

The analysis of Alzheimer’s disease projections from 2021 to 2030 highlights notable disparities among various SDI levels and continents. These differences are apparent in the age-standardized Incidence Rate (ASIR), age-standardized Death Rate (ASDR), and age-standardized DALY rate.

In 2030, countries with a SDI of 0.8 or higher exhibit notably higher values in ASIR, ASDR, and age-standardized DALY rate compared to countries with an SDI below 0.8 (Supplementary Figure 2). This suggests that regions with higher SDI face a greater burden of Alzheimer’s disease. Similarly, when using an SDI threshold of 0.7, the same patterns emerge, with higher SDI categories showing significantly higher ASIR, ASDR, and age-standardized DALY rates (Supplementary Figure 3). The consistent and significant differences observed between these groups at both thresholds highlight the strong influence of socioeconomic status on the burden of Alzheimer’s disease.

Further breakdown by specific SDI categories (low, middle, high-middle, high, and low-middle) highlights significant differences. High-SDI regions show the highest values for ASIR, ASDR, and age-standardized DALY rates, while low-SDI regions have the lowest values. All comparisons demonstrate highly significant *p*-values (Supplementary Figure 4), indicating that higher SDI is associated with a greater burden of disease.

The correlation analysis between Alzheimer’s disease burden and SDI across five continents reveals diverse relationships. Oceania exhibits the highest correlation with an R^2^ of 0.203 and a *p*-value of 0.0463, indicating a moderate positive association (Supplementary Figure 5). This implies that in Oceania, regions with higher SDI tend to have higher ASIR of Alzheimer’s disease. In contrast, Africa demonstrates a negligible correlation (R^2^ = 0.008, *p* = 0.0699), suggesting that SDI has minimal impact on ASIR in this continent. The other continents display weak correlations, with America (R^2^ = 0.088, p = 0.0699) and Asia (R^2^ = 0.055, *p* = 0.1093) showing subtle but not significant trends.

In terms of ASDR, the trends are less pronounced but still notable. Oceania exhibits a moderate correlation with an R^2^ of 0.144 and a *p*-value of 0.0984 (Supplementary Figure 5), suggesting a relationship between higher SDI and death rates. America shows a weak correlation (R^2^ = 0.091, *p* = 0.0652), while Africa, Asia, and Europe demonstrate very low correlations. Africa, for instance, has an R^2^ of 0.099 and a p-value of 0.0539, indicating almost no relationship (Supplementary Figure 5).

The age-standardized DALY rate in Oceania shows a notable correlation, with an R^2^ of 0.204 and a p-value of 0.0459 (Supplementary Figure 5). This suggests a strong association between higher SDI and increased DALY rates. In contrast, other continents exhibit less prominent trends, with America displaying a weak correlation (R^2^ = 0.099, *p* = 0.0539) and Africa, Asia, and Europe showing minimal correlations (Supplementary Figure 5).

## 4 Discussion

Our research forecasts indicate that between 2021 and 2030, the global burden of Alzheimer’s disease will exhibit notable trends and disparities across different regions, sexes, and socio-demographic levels. Our findings reveal a projected decline in the burden of Alzheimer’s disease measured by DALYs, with decreases in ASIR, ASDR, and age-standardized DALY rates globally. This decline is consistent across both sexes, indicating a general trend of reduced burden over the next decade. However, there are significant regional variations, with Andean Latin America and the Caribbean showing increasing trends, while Eastern Europe and East Asia are projected to experience declines. Country-specific analysis highlights substantial disparities, with Cyprus and Serbia facing rising burdens, while Bahrain and Armenia are expected to see reductions. Correlation analysis emphasizes the impact of socioeconomic development on Alzheimer’s disease burden, with higher SDI regions consistently showing greater disease metrics. Oceania shows a strong positive correlation between SDI and burden, while Africa exhibits minimal correlation, suggesting different influencing factors. These findings stress the importance of targeted public health interventions and policies to address the diverse and evolving burden of Alzheimer’s disease globally.

The projected data suggests a notable decrease in the global burden of Alzheimer’s disease from 2021 to 2030, with decreasing trends in EAPC for DALYs, death rates, and incidence rates. These reductions indicate that advancements in medical interventions, early detection, and public health strategies are contributing to better disease management. The data also reflects the impact of increased awareness and preventive measures targeting risk factors associated with Alzheimer’s disease. While there has been a consistent decline in these key metrics, the overall burden of the disease remains significant. The projections emphasize the crucial need for continued investment in healthcare systems and ongoing research into innovative treatment methods to further alleviate the global impact of Alzheimer’s disease (21). Despite these positive trends, the projected burden for 2030 highlights the persistent challenges that require tailored interventions and policies to address the diverse needs of populations (22).

The projected decline in the global burden of Alzheimer’s disease across both sexes from 2021 to 2030 highlights several critical mechanisms that may be contributing to this trend. The decreasing EAPC for death and incidence rates in both males and females suggests that advancements in early detection and intervention strategies are becoming more effective. Enhanced screening programs, increased public awareness, and improved access to healthcare are likely playing significant roles in identifying Alzheimer’s disease at earlier stages, allowing for timely and more effective management (23). Furthermore, advancements in pharmacological treatments and non-pharmacological interventions, such as cognitive training and lifestyle modifications, may also be contributing to the observed declines (24).

The differential EAPC between males and females could be indicative of varying biological and social factors influencing disease progression and outcomes. Hormonal differences, particularly the protective effects of estrogen in females, might explain the slightly lower decline rate in incidence for females compared to males (25). Additionally, sex-specific differences in lifestyle factors, such as smoking and alcohol consumption, along with differing healthcare-seeking behaviors, may also contribute to these trends. Social support structures, which often differ between genders, could further influence the observed disparities. These projections underscore the importance of continued investment in gender-specific research and public health strategies to sustain and accelerate the progress in reducing the global burden of Alzheimer’s disease (26).

The anticipated regional variations in the burden of Alzheimer’s disease from 2021 to 2030 highlight the intricate interplay of socio-economic, cultural, and healthcare factors shaping this neurodegenerative disorder. In regions like Andean Latin America, Southern Latin America, and the Caribbean, the highest positive EAPC indicates a growing burden driven by various factors. Economic development in these areas may coincide with urbanization and lifestyle modifications that heighten risk factors such as unhealthy diet, sedentary lifestyle, and a higher prevalence of metabolic conditions like obesity and diabetes (27). Moreover, cultural aspects, including the social stigma linked to mental health and cognitive decline, could lead to delays in diagnosis and treatment. These regions also encounter challenges in healthcare infrastructure, with limited access to specialized care and diagnostic facilities, further compounding the burden of the disease (28).

Conversely, the significant negative EAPC in regions like Eastern and Central Europe and East Asia indicates successful management and prevention strategies. These areas may benefit from advanced healthcare systems, comprehensive public health policies, and widespread awareness campaigns that promote early detection and risk factor modification (29). For example, policies promoting cardiovascular health, reducing smoking rates, and managing hypertension and diabetes likely contribute to these positive trends. Moreover, cultural attitudes towards aging and mental health in these regions may encourage proactive healthcare-seeking behaviors and better support systems for older adults (30).

The apparent contradiction between the lowest ASRs in High-income Asia Pacific and the overall trend of higher ASIR, ASDR, and DALY rates in higher SDI regions reflects the multifaceted influence of socio-economic factors. High SDI regions generally exhibit higher disease burdens due to extended life expectancies, increased urbanization, and lifestyle factors like reduced physical activity and dietary changes. However, within these high SDI regions, robust healthcare systems and effective public health interventions, as seen in High-income Asia Pacific, can mitigate these risks and result in lower ASRs. This demonstrates the critical role of tailored healthcare policies and resources in managing disease burden effectively, even in high-risk settings.

The disparities highlighted by ASR values, with Central Sub-Saharan Africa showing the highest burden, underscore the impact of socio-economic challenges, political instability, and limited healthcare resources. In contrast, regions with the lowest ASRs, such as High-income Asia Pacific, demonstrate the advantages of robust healthcare infrastructure, economic stability, and effective disease management programs. These findings emphasize the need for tailored, region-specific interventions that consider the unique socio-cultural and economic contexts to effectively address and mitigate the global burden of Alzheimer’s disease (31).

The projected disparities in the burden of Alzheimer’s disease across different countries from 2021 to 2030 underscore the impact of various socio-economic, cultural, and healthcare factors. Countries like Cyprus, Serbia, and Montenegro, exhibiting the highest positive EAPCs, may be facing increasing burdens due to a combination of aging populations, shifts in dietary patterns, and a higher prevalence of lifestyle-related risk factors such as hypertension and diabetes. Economic transformations in these areas could drive urbanization and lifestyle changes that worsen these risk factors (32). Furthermore, healthcare systems in these nations may be grappling with challenges related to resource allocation and access to advanced diagnostic tools, leading to delays in the early detection and effective management of Alzheimer’s disease (33).

In contrast, countries such as Bahrain, Armenia, and Qatar, which exhibit significant negative EAPCs, likely benefit from robust healthcare infrastructure, proactive public health policies, and higher levels of health literacy. These nations may have implemented comprehensive screening programs and public health campaigns that emphasize the importance of early detection and lifestyle modifications to reduce risk. Cultural factors, including strong family support systems and societal attitudes towards aging and mental health, may also play a role in promoting early intervention and ongoing care (34). Furthermore, economic stability in these regions facilitates better access to healthcare services and medications, contributing to more effective disease management. The stark differences in ASR values, with countries like Cyprus and North Macedonia showing the highest burdens, underscore the urgent need for targeted interventions that address the unique socio-economic and cultural contexts of each country. These findings emphasize the critical importance of tailored public health strategies, enhanced healthcare resources, and international collaboration to mitigate the global burden of Alzheimer’s disease (35).

The projection for the distribution and correlation analysis of Alzheimer’s disease from 2021 to 2030 highlights significant socio-demographic disparities and their intricate mechanisms. Higher SDI regions consistently exhibit higher ASIR, ASDR, and age-standardized DALY rates, indicating the complex influence of socio-economic status. In regions with high SDI, factors such as advanced healthcare systems contribute to improved diagnosis and reporting, while longer life expectancies expand the population at risk for Alzheimer’s disease. Urbanization and lifestyle changes in these areas may also increase risk factors like poor diet and sedentary behavior. On the other hand, low-SDI regions, despite lower disease metrics, may face challenges such as underdiagnosis and limited healthcare access, leading to an underestimation of the true burden. The analysis further demonstrates that Oceania shows a strong positive correlation between SDI and disease burden, highlighting how improved longevity and healthcare access contribute to a higher prevalence of Alzheimer’s disease (36). In contrast, Africa’s minimal correlation implies that socio-economic advancements alone are insufficient; issues such as healthcare infrastructure gaps, cultural perceptions of aging, and limited public health efforts are also significant factors. These insights emphasize the importance of targeted interventions that address not just economic disparities but also strengthen healthcare systems and promote cultural changes to effectively manage Alzheimer’s disease in diverse global settings (37).

The study’s main strength lies in its thorough analysis of the global burden of Alzheimer’s disease, examining various demographic factors such as age, gender, countries, regions, and socio-economic levels. The data, collected from 1990 to 2021 and projected through 2030, offers a comprehensive view of the disease (38, 39). This approach facilitates a nuanced understanding of Alzheimer’s disease in different contexts, enabling targeted public health interventions. The analysis is robust, supported by multiple intergroup tests that enhance the reliability and validity of the findings. Additionally, the global perspective of the study allows for comparisons across diverse regions, shedding light on both similarities and differences in the burden of Alzheimer’s disease (40, 41).

However, this study also has several limitations. The accuracy of our estimates may be compromised by unequal data availability across countries and regions, particularly in lower SDI areas where comprehensive Alzheimer’s disease surveillance systems and population-based registries are lacking. This discrepancy can introduce uncertainty into our projections. Additionally, the reliance on GBD data, which uses ICD codes for case definitions, may lead to biases due to variations in diagnostic criteria and healthcare access. Such differences can affect the distribution of Alzheimer’s disease, potentially underestimating mild or asymptomatic cases (42). Moreover, the GBD database does not fully account for socio-economic changes, cultural variations, or other contextual factors that may influence the burden of Alzheimer’s disease. This omission necessitates cautious interpretation of our results, as these unmeasured variables could impact our findings. Despite these limitations, our study provides valuable insights into the global trends and disparities in Alzheimer’s disease burden, underscoring the need for continued research and improved data collection methods to enhance the accuracy of future projections (43).

## 5 Conclusion

The projected burden of Alzheimer’s disease from 2021 to 2030 is expected to vary significantly on a global scale. While there is a general decline in ASIR, ASDR, and age-standardized DALY rates, indicating improvements in disease management and prevention, certain regions such as Andean Latin America and the Caribbean are predicted to experience increasing trends, revealing regional disparities. The burden is particularly higher in regions with higher SDI, showcasing the combined impact of advanced healthcare and longer life expectancies. On the other hand, regions with lower SDI, despite displaying lower disease metrics, may face challenges related to underdiagnosis and limited healthcare resources. These findings emphasize the necessity of tailored public health interventions that take into account socio-economic, cultural, and healthcare contexts in order to effectively address and alleviate the global burden of Alzheimer’s disease.

## Data Availability

The original contributions presented in the study are included in the article/Supplementary material, further inquiries can be directed to the corresponding author.
