# Supplementary material for: Assessing and projecting the global impacts of Alzheimer’s disease
Source: Front Public Health. 2025 Jan 15;12:1453489. doi: 10.3389/fpubh.2024.1453489 (PMC11775756; doi:10.3389/fpubh.2024.1453489)
Supplement: Supplementary file 1 [file Supplementary_file_1.docx]

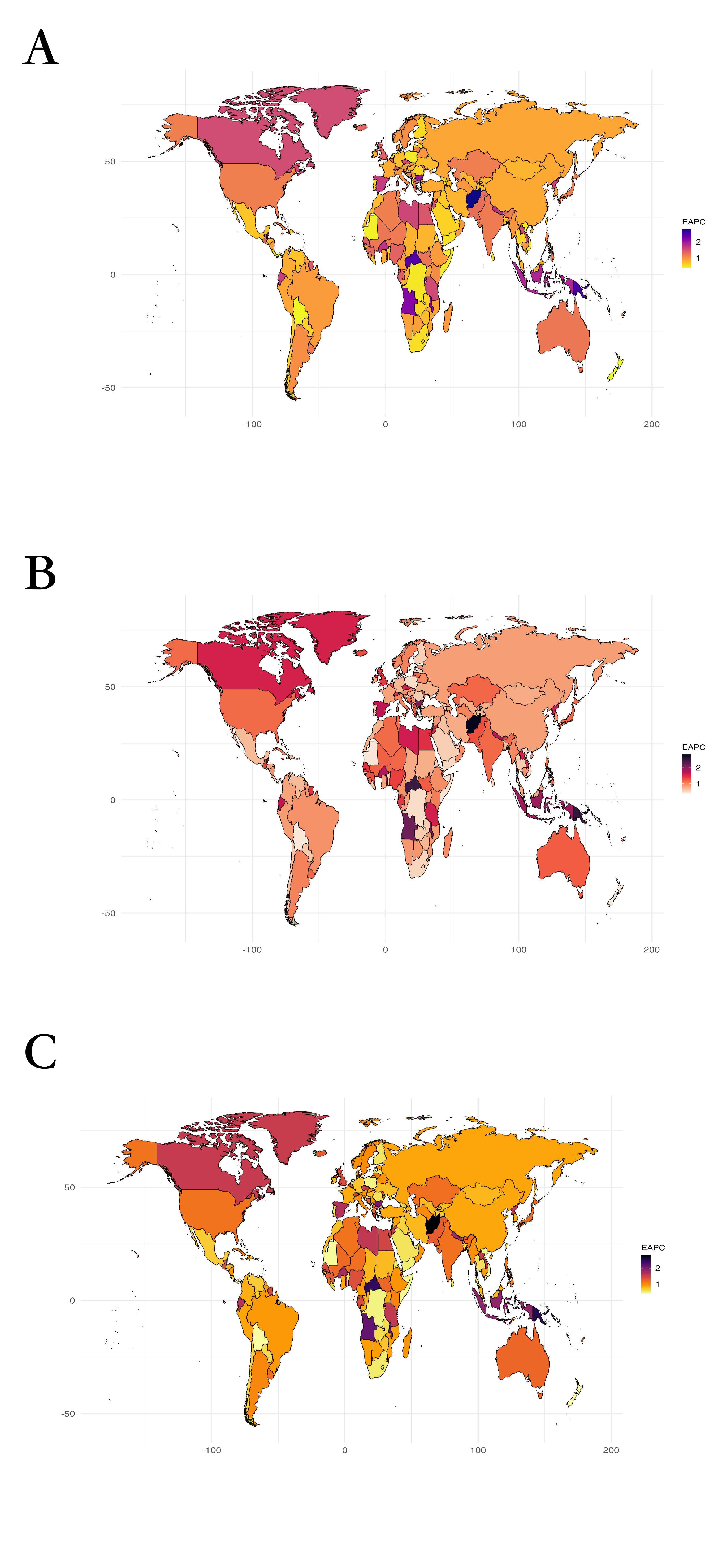


**SFigure 1: Projected global burden of age-standardized rate of Alzheimer's Disease in 2030, by countries. (A) ASIR (B) ASDR (C) age-standardized rate.** DALY = disability adjusted life-year. ASIR = age standardized incidence rate. ASDR = age standardized death rate. ASRs = age standardized rates.


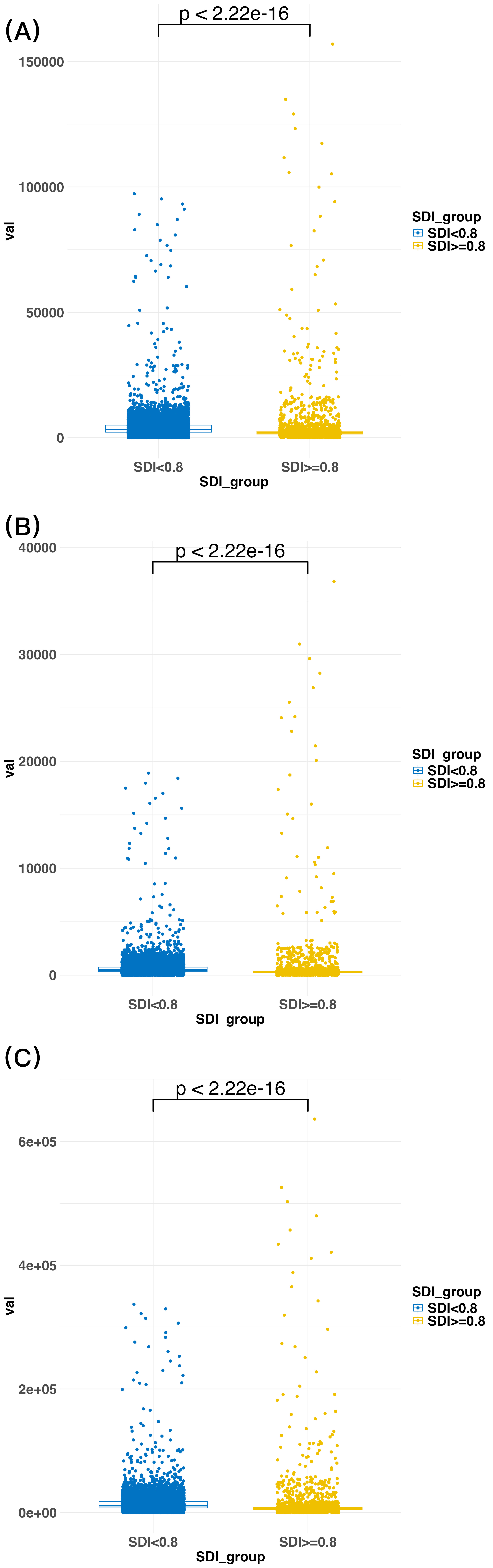


**SFigure 2: Comparison between different SDI groups of 0.8 with box plots of predicted ASRs for the global burden of Alzheimer's Disease in 2030. (A) ASIR (B) ASDR (C) Age-standardized DALY rate.** DALY = disability adjusted life-year. ASIR = age standardized incidence rate. ASDR = age standardized death rate. ASRs = age standardized rates. The box extends from the 25th percentile to the 75th percentile, with the center line indicating the median; the bottom whisker line indicates the minimum value, and the top whisker line indicates the 75th percentile plus 1.5 times the interquartile distance (the distance between the 25th and 75th percentiles). The p-values from the Kruskal-Wallis test for both groups are labeled in the figure. Statistical significance is indicated by p < 0.05.


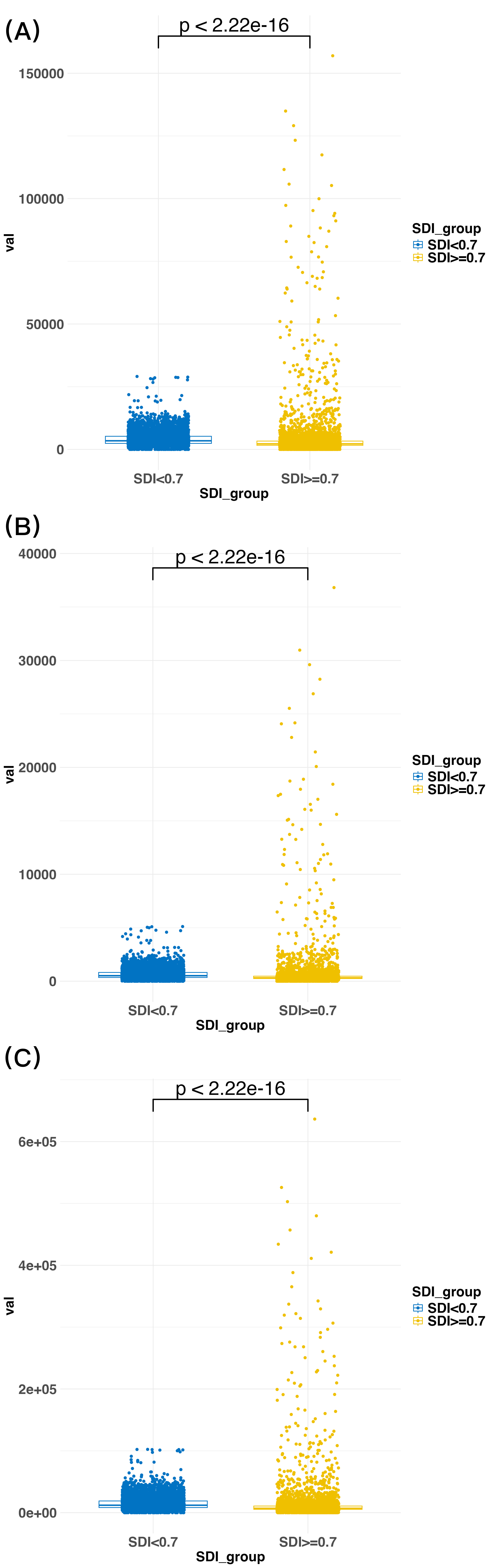


**SFigure 3: Comparison between different SDI groups of 0.7 with box plots of predicted ASRs for the global burden of Alzheimer's Disease in 2030. (A) ASIR (B) ASDR (C) Age-standardized DALY rate.** DALY = disability adjusted life-year. ASIR = age standardized incidence rate. ASDR = age standardized death rate. ASRs = age standardized rates. The box extends from the 25th percentile to the 75th percentile, with the center line indicating the median; the bottom whisker line indicates the minimum value, and the top whisker line indicates the 75th percentile plus 1.5 times the interquartile distance (the distance between the 25th and 75th percentiles). The p-values from the Kruskal-Wallis test for both groups are labeled in the figure. Statistical significance is indicated by p < 0.05.


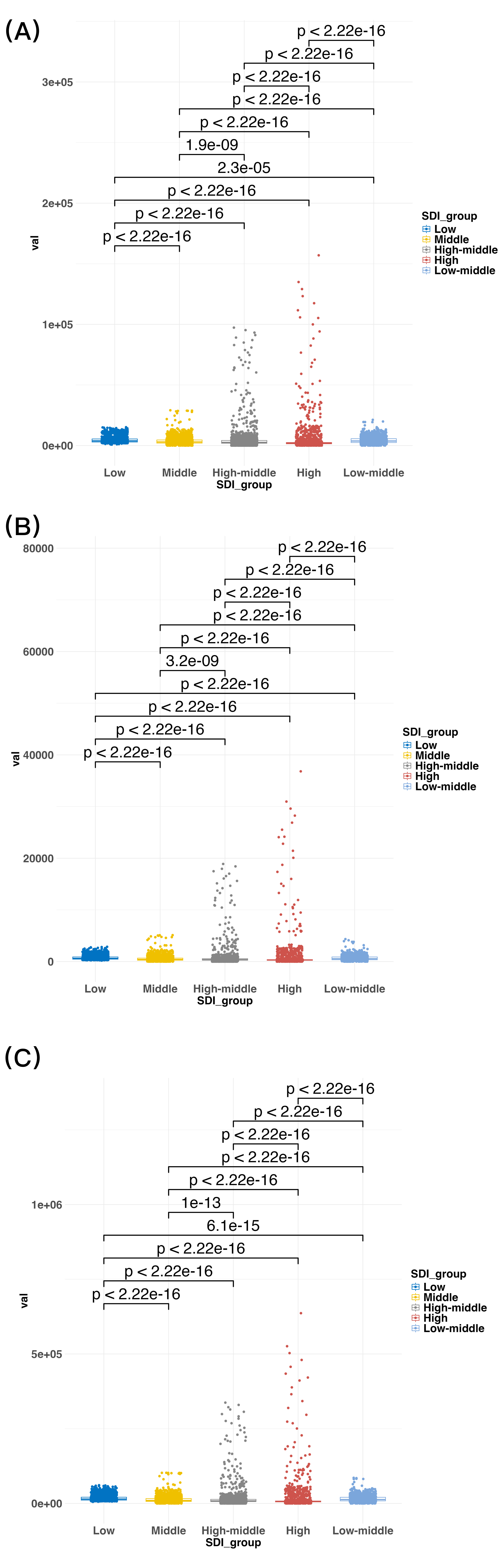


**SFigure 4: Comparison between different SDI groups with box plots of predicted ASRs for the global burden of Alzheimer's Disease in 2030. (A) ASIR (B) ASDR (C) Age-standardized DALY rate.** DALY = disability adjusted life-year. ASIR = age standardized incidence rate. ASDR = age standardized death rate. ASRs = age standardized rates. The box extends from the 25th percentile to the 75th percentile, with the center line indicating the median; the bottom whisker line indicates the minimum value, and the top whisker line indicates the 75th percentile plus 1.5 times the interquartile distance (the distance between the 25th and 75th percentiles). The p-values from the Kruskal-Wallis test for both groups are labeled in the figure. Statistical significance is indicated by p < 0.05.


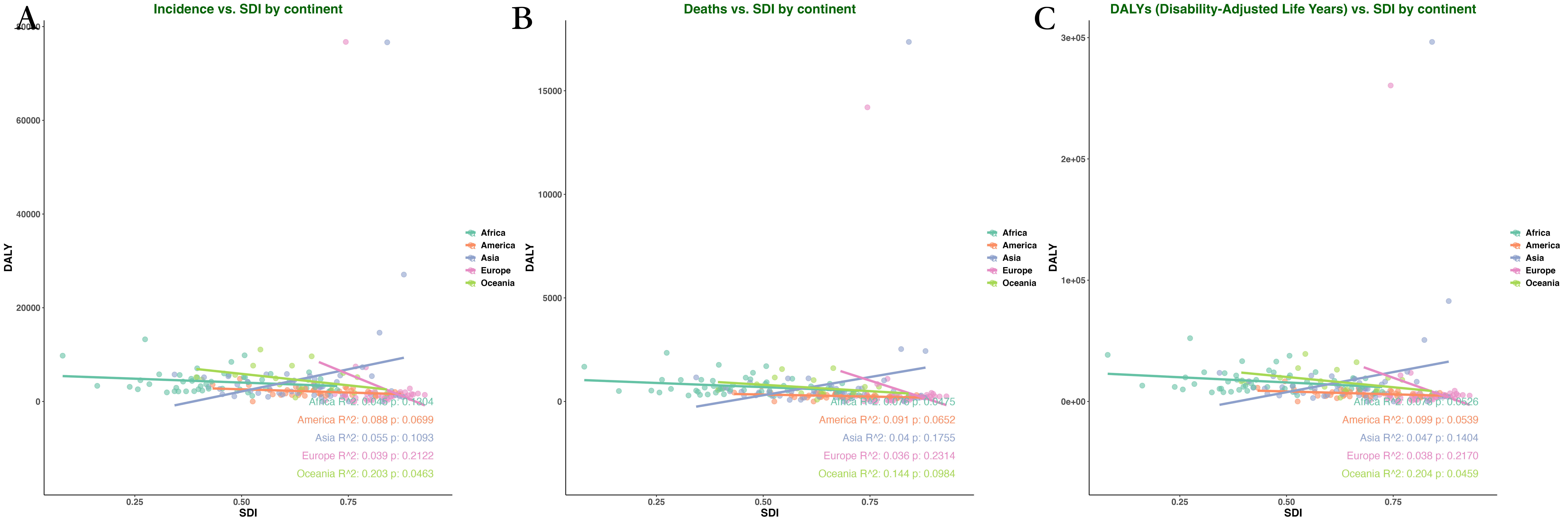


**SFigure 5: Correlation analysis between projected global burden of age-standardized rate of Alzheimer's Disease and SDI levels in 2030, by continents. (A) ASIR (B) ASDR (C) age-standardized rate.** DALY = disability adjusted life-year. ASIR = age standardized incidence rate. ASDR = age standardized death rate. ASRs = age standardized rates.
