# Supplementary material for: Assessing and projecting the global impacts of Alzheimer’s disease
Source: Front Public Health. 2025 Jan 15;12:1453489. doi: 10.3389/fpubh.2024.1453489 (PMC11775756; doi:10.3389/fpubh.2024.1453489)
Supplement: Supplementary file 2 [file Table_1.docx]

**STable 1. The projected age-standardized rates of Alzheimer's Disease in 1990 and 2030 in different regions.**

| location | Deaths | | DALYs (Disability-Adjusted Life Years) | | Incidence | |
| --- | --- | --- | --- | --- | --- | --- |
|  | 1990 | 2030 | 1990 | 2030 | 1990 | 2030 |
| Global | 14775.89 (14760.61, 14791.18) | 7210.92 (6730.65, 7691.19) | 644.16 (640.90, 647.43) | 299.92 (274.94, 324.91) | 4483.76 (4475.36, 4492.18) | 2199.67 (2054.49, 2344.86) |
| High SDI | 11924.98 (11904.03, 11945.95) | 4648.20 (4167.93, 5128.46) | 537.10 (532.62, 541.61) | 208.69 (181.69, 235.70) | 3791.43 (3779.61, 3803.28) | 1328.72 (1168.04, 1489.41) |
| High-middle SDI | 19765.70 (19723.27, 19808.22) | 6442.73 (5063.42, 7822.04) | 894.04 (884.75, 903.41) | 244.15 (173.74, 314.57) | 5768.05 (5745.27, 5790.92) | 2177.52 (1779.83, 2575.21) |
| Middle SDI | 18183.06 (18144.79, 18221.41) | 10481.59 (10030.08, 10933.10) | 769.83 (761.73, 778.01) | 434.37 (410.83, 457.91) | 5249.41 (5228.91, 5269.97) | 3219.02 (3055.21, 3382.84) |
| Low-middle SDI | 11647.67 (11614.20, 11681.22) | 9800.55 (9440.64, 10160.45) | 481.52 (474.58, 488.54) | 420.53 (405.62, 435.44) | 3708.16 (3689.30, 3727.11) | 2842.88 (2720.94, 2964.81) |
| Low SDI | 17552.18 (17463.77, 17640.98) | 15730.25 (14992.73, 16467.77) | 736.93 (718.28, 755.99) | 720.13 (682.26, 757.99) | 5183.45 (5135.45, 5231.86) | 4231.99 (4034.98, 4429.00) |
| Andean Latin America | 4990.84 (4919.48, 5063.12) | 4583.57 (4410.91, 4756.22) | 203.67 (189.79, 218.47) | 187.97 (181.53, 194.41) | 1498.90 (1459.66, 1539.09) | 1367.18 (1310.71, 1423.65) |
| Australasia | 12298.69 (12136.38, 12462.87) | 3338.99 (2566.57, 4111.42) | 546.19 (511.75, 582.57) | 120.69 (71.35, 170.03) | 3901.39 (3810.44, 3994.22) | 1066.49 (889.38, 1243.60) |
| Caribbean | 11323.42 (11178.45, 11470.06) | 5047.12 (4390.30, 5703.93) | 499.48 (468.62, 532.09) | 206.91 (174.67, 239.16) | 3649.12 (3567.46, 3732.44) | 1638.10 (1434.20, 1841.99) |
| Central Asia | 9812.17 (9709.75, 9915.53) | 6561.52 (5631.14, 7491.90) | 421.51 (400.13, 443.87) | 281.39 (257.89, 304.89) | 3200.44 (3141.98, 3259.86) | 1682.71 (1292.82, 2072.60) |
| Central Europe | 17056.51 (16958.01, 17155.50) | 2242.78 (84.33, 4401.23) | 756.44 (735.24, 778.15) | 38.33 (0.05, 150.56) | 5690.62 (5633.95, 5747.80) | 761.82 (79.21, 1444.42) |
| Central Latin America | 7693.82 (7641.28, 7746.68) | 5659.25 (5383.87, 5934.63) | 299.11 (288.89, 309.64) | 217.42 (209.14, 225.69) | 2730.38 (2698.86, 2762.22) | 1927.95 (1803.11, 2052.80) |
| Central Sub-Saharan Africa | 26695.60 (26292.10, 27104.57) | 23415.63 (22026.59, 24804.68) | 1097.82 (1014.59, 1186.82) | 1042.61 (983.46, 1101.76) | 7358.82 (7148.96, 7574.13) | 5704.88 (5353.12, 6056.63) |
| East Asia | 58702.33 (58545.93, 58859.11) | 16026.62 (10318.51, 21734.73) | 2919.45 (2882.79, 2956.52) | 691.46 (396.82, 986.10) | 14821.21 (14743.39, 14899.40) | 4755.73 (3123.08, 6388.38) |
| Eastern Europe | 16731.21 (16649.34, 16813.43) | 337.20 (0.05, 2212.57) | 703.41 (686.20, 720.99) | 0.05 (0.05, 87.35) | 5637.54 (5590.17, 5685.26) | 0.05 (0.05, 599.98) |
| Eastern Sub-Saharan Africa | 23275.22 (23076.87, 23475.10) | 14109.52 (13438.59, 14780.44) | 1002.02 (959.57, 1046.10) | 633.17 (602.00, 664.35) | 6403.44 (6299.64, 6508.78) | 3491.55 (3316.79, 3666.31) |
| High-income Asia Pacific | 13761.36 (13698.44, 13824.54) | 2637.74 (2257.17, 3018.30) | 647.32 (633.37, 661.54) | 139.19 (121.16, 157.22) | 4045.14 (4011.25, 4079.28) | 693.84 (558.54, 829.13) |
| High-income North America | 10710.28 (10680.99, 10739.65) | 6293.00 (5860.31, 6725.69) | 458.72 (452.75, 464.76) | 265.67 (246.04, 285.30) | 3452.87 (3436.09, 3469.72) | 1837.79 (1675.21, 2000.38) |
| Oceania | 30507.37 (29192.13, 31873.45) | 13319.76 (12089.81, 14549.71) | 1218.77 (960.74, 1531.98) | 449.17 (375.19, 523.16) | 9424.54 (8700.11, 10200.34) | 4184.36 (3855.20, 4513.52) |
| Southeast Asia | 13756.65 (13696.11, 13817.43) | 7927.48 (7544.39, 8310.57) | 547.17 (534.78, 559.80) | 279.03 (256.22, 301.84) | 4432.70 (4398.43, 4467.21) | 2263.44 (2163.37, 2363.50) |
| Southern Latin America | 9685.65 (9589.87, 9782.25) | 6310.95 (6027.38, 6594.51) | 408.08 (388.35, 428.64) | 252.39 (237.44, 267.35) | 3418.51 (3361.65, 3476.19) | 2211.47 (2104.46, 2318.47) |
| Southern Sub-Saharan Africa | 10137.80 (9994.02, 10283.44) | 15620.00 (14235.86, 17004.14) | 420.44 (390.75, 452.08) | 634.90 (559.92, 709.88) | 3223.10 (3142.00, 3306.07) | 4706.07 (4287.85, 5124.29) |
| Tropical Latin America | 17406.79 (17303.68, 17510.42) | 8427.64 (7872.65, 8982.62) | 739.44 (717.91, 761.53) | 326.23 (286.64, 365.81) | 5030.47 (4975.31, 5086.16) | 2626.11 (2555.75, 2696.48) |
| Western Europe | 13059.94 (13026.97, 13092.98) | 4566.30 (3814.27, 5318.33) | 624.59 (617.27, 631.98) | 222.59 (179.75, 265.43) | 4106.01 (4087.65, 4124.44) | 1422.18 (1175.90, 1668.45) |

**STable 2. The projected EAPC of Alzheimer's Disease from 2022 to 2030 in different regions, by sexes.**

| location | DALYs (Disability-Adjusted Life Years) | | | Deaths | | | Incidence | | |
| --- | --- | --- | --- | --- | --- | --- | --- | --- | --- |
|  | Both | Female | Male | Both | Female | Male | Both | Female | Male |
| Global | -1.4375 (-1.4548, -1.4201) | -1.1938 (-1.2057, -1.1818) | -1.8351 (-1.8633, -1.8068) | -1.7982 (-1.8254, -1.7711) | -1.4261 (-1.4432, -1.4091) | -2.2756 (-2.3191, -2.2321) | -1.2718 (-1.2854, -1.2583) | -1.0309 (-1.0398, -1.0219) | -1.7293 (-1.7544, -1.7042) |
| High SDI | -1.3768 (-1.3927, -1.3609) | -0.7272 (-0.7316, -0.7227) | -3.1339 (-3.2164, -3.0513) | -1.2757 (-1.2893, -1.2620) | -0.5275 (-0.5299, -0.5252) | -3.4527 (-3.5529, -3.3525) | -1.5120 (-1.5312, -1.4928) | -0.8066 (-0.8121, -0.8012) | -3.6626 (-3.7753, -3.5497) |
| High-middle SDI | -3.5088 (-3.6123, -3.4053) | -3.0408 (-3.1185, -2.9631) | -4.6870 (-4.8717, -4.5019) | -4.4899 (-4.6594, -4.3201) | -3.6581 (-3.7706, -3.5456) | -6.3052 (-6.6401, -5.9691) | -2.5446 (-2.5990, -2.4902) | -2.1194 (-2.1571, -2.0816) | -3.7177 (-3.8338, -3.6014) |
| Middle SDI | -1.5541 (-1.5744, -1.5338) | -2.2618 (-2.3047, -2.2188) | -0.5016 (-0.5037, -0.4995) | -1.7881 (-1.8150, -1.7613) | -2.3266 (-2.3720, -2.2811) | -0.5668 (-0.5695, -0.5642) | -1.4431 (-1.4606, -1.4256) | -1.9515 (-1.9835, -1.9195) | -0.4188 (-0.4202, -0.4173) |
| Low-middle SDI | 0.3827 (0.3815, 0.3840) | 0.1010 (0.1009, 0.1011) | 0.7780 (0.7729, 0.7831) | 0.6081 (0.6050, 0.6112) | 0.4233 (0.4218, 0.4248) | 0.9058 (0.8989, 0.9127) | 0.0898 (0.0897, 0.0899) | -0.3971 (-0.3984, -0.3958) | 0.7549 (0.7502, 0.7597) |
| Low SDI | 1.4387 (1.4213, 1.4560) | 1.0115 (1.0030, 1.0201) | 1.9073 (1.8768, 1.9379) | 1.7575 (1.7315, 1.7834) | 1.4537 (1.4359, 1.4714) | 2.1331 (2.0949, 2.1713) | 1.1660 (1.1546, 1.1774) | 0.5683 (0.5656, 0.5710) | 1.8344 (1.8061, 1.8626) |
| Andean Latin America | 0.9371 (0.9298, 0.9445) | 1.2822 (1.2684, 1.2961) | 0.8081 (0.8026, 0.8136) | 0.7193 (0.7150, 0.7237) | 1.1517 (1.1406, 1.1628) | 0.6186 (0.6154, 0.6218) | 0.9506 (0.9430, 0.9582) | 1.4008 (1.3843, 1.4172) | 0.6428 (0.6393, 0.6463) |
| Australasia | -1.6019 (-1.6234, -1.5804) | -0.8865 (-0.8931, -0.8799) | -4.5602 (-4.7350, -4.3850) | -2.6780 (-2.7383, -2.6178) | -1.6651 (-1.6884, -1.6419) | -7.0716 (-7.4933, -6.6480) | -0.8525 (-0.8586, -0.8464) | 0.0190 (0.0190, 0.0190) | -3.6428 (-3.7543, -3.5312) |
| Caribbean | 0.5931 (0.5902, 0.5961) | 0.3713 (0.3702, 0.3725) | 0.9084 (0.9015, 0.9153) | 0.5578 (0.5552, 0.5605) | 0.3511 (0.3500, 0.3521) | 0.8815 (0.8749, 0.8880) | 0.7553 (0.7505, 0.7600) | 0.5498 (0.5473, 0.5524) | 1.0319 (1.0229, 1.0408) |
| Central Asia | -1.8372 (-1.8655, -1.8088) | -0.9292 (-0.9364, -0.9219) | -6.7460 (-7.1296, -6.3609) | -1.8902 (-1.9202, -1.8602) | -0.8309 (-0.8367, -0.8251) | -7.0124 (-7.4270, -6.5959) | -2.8444 (-2.9123, -2.7764) | -1.7739 (-1.8003, -1.7474) | -8.8625 (-9.5267, -8.1935) |
| Central Europe | -12.0323 (-13.2652, -10.7819) | -11.7667 (-12.9449, -10.5725) | -11.5728 (-12.7119, -10.4188) | -17.4959 (-20.1515, -14.7520) | -15.2338 (-17.2295, -13.1899) | -19.6474 (-23.0291, -16.1170) | -11.7452 (-12.9190, -10.5555) | -11.8058 (-12.9919, -10.6034) | -11.1788 (-12.2407, -10.1041) |
| Central Latin America | 0.5100 (0.5078, 0.5122) | 0.1845 (0.1842, 0.1848) | 1.0250 (1.0162, 1.0338) | 0.3524 (0.3513, 0.3534) | 0.0068 (0.0068, 0.0068) | 0.9260 (0.9188, 0.9332) | 0.5536 (0.5510, 0.5562) | 0.2568 (0.2563, 0.2574) | 1.0443 (1.0351, 1.0534) |
| Central Sub-Saharan Africa | -0.2830 (-0.2837, -0.2823) | -0.7919 (-0.7972, -0.7867) | 0.7522 (0.7474, 0.7569) | 0.0273 (0.0273, 0.0273) | -0.2978 (-0.2986, -0.2971) | 0.9006 (0.8938, 0.9074) | -0.7369 (-0.7414, -0.7323) | -1.3278 (-1.3426, -1.3130) | 0.3760 (0.3748, 0.3772) |
| East Asia | -2.7709 (-2.8354, -2.7064) | -2.9286 (-3.0006, -2.8565) | -1.7780 (-1.8045, -1.7514) | -3.0900 (-3.1702, -3.0097) | -3.0420 (-3.1198, -2.9643) | -2.2873 (-2.3312, -2.2433) | -2.9029 (-2.9737, -2.8321) | -3.0074 (-3.0834, -2.9314) | -1.9089 (-1.9395, -1.8783) |
| Eastern Europe | -16.3071 (-18.6030, -13.9464) | -14.5649 (-16.3851, -12.7052) | -27.6057 (-34.5776, -19.8909) | -19.0569 (-22.2295, -15.7549) | -15.5543 (-17.6373, -13.4186) | -53.4727 (-71.0456, -25.2344) | -18.4027 (-21.3524, -15.3424) | -16.4407 (-18.7757, -14.0387) | -57.4891 (-77.9197, -18.1543) |
| Eastern Sub-Saharan Africa | -0.0259 (-0.0259, -0.0259) | 0.0110 (0.0110, 0.0110) | -0.1177 (-0.1178, -0.1176) | 0.1719 (0.1717, 0.1722) | 0.2440 (0.2435, 0.2445) | 0.0014 (0.0014, 0.0014) | -0.3794 (-0.3806, -0.3782) | -0.4017 (-0.4031, -0.4004) | -0.3275 (-0.3284, -0.3266) |
| High-income Asia Pacific | -2.0629 (-2.0986, -2.0271) | -0.7482 (-0.7529, -0.7435) | -7.2142 (-7.6531, -6.7731) | -0.7750 (-0.7801, -0.7700) | 0.4483 (0.4466, 0.4500) | -6.0536 (-6.3622, -5.7440) | -2.7236 (-2.7859, -2.6612) | -1.2818 (-1.2956, -1.2680) | -8.3863 (-8.9805, -7.7882) |
| High-income North America | 0.2162 (0.2158, 0.2166) | 0.4548 (0.4530, 0.4565) | -1.1713 (-1.1828, -1.1598) | -0.5813 (-0.5841, -0.5784) | -0.4796 (-0.4815, -0.4777) | -1.9498 (-1.9817, -1.9178) | 0.5365 (0.5340, 0.5389) | 0.9516 (0.9440, 0.9592) | -1.2983 (-1.3125, -1.2842) |
| Oceania | 0.3937 (0.3924, 0.3951) | 0.5703 (0.5675, 0.5730) | 0.1330 (0.1328, 0.1331) | -0.5430 (-0.5455, -0.5405) | -0.1416 (-0.1417, -0.1414) | -1.1481 (-1.1592, -1.1370) | 0.5631 (0.5604, 0.5657) | 0.7396 (0.7350, 0.7442) | 0.3099 (0.3091, 0.3107) |
| Southeast Asia | -2.2938 (-2.3379, -2.2496) | -2.5134 (-2.5665, -2.4604) | -1.7305 (-1.7556, -1.7053) | -3.0312 (-3.1084, -2.9540) | -3.1568 (-3.2405, -3.0730) | -2.6001 (-2.6569, -2.5433) | -2.4581 (-2.5088, -2.4073) | -2.6533 (-2.7124, -2.5942) | -1.9751 (-2.0078, -1.9423) |
| Southern Latin America | 0.7651 (0.7602, 0.7700) | 0.3025 (0.3017, 0.3032) | 1.7885 (1.7617, 1.8154) | 0.3688 (0.3677, 0.3700) | -0.0824 (-0.0825, -0.0824) | 1.5200 (1.5006, 1.5394) | 0.9995 (0.9911, 1.0079) | 0.5603 (0.5577, 0.5630) | 1.9250 (1.8939, 1.9561) |
| Southern Sub-Saharan Africa | 0.0033 (0.0033, 0.0033) | -0.4432 (-0.4449, -0.4416) | 1.2028 (1.1907, 1.2150) | -0.6755 (-0.6794, -0.6717) | -1.1202 (-1.1307, -1.1097) | 0.6163 (0.6131, 0.6195) | 0.0592 (0.0592, 0.0593) | -0.4523 (-0.4540, -0.4506) | 1.3580 (1.3425, 1.3735) |
| Tropical Latin America | -1.0657 (-1.0753, -1.0562) | -1.3925 (-1.4088, -1.3762) | -0.3809 (-0.3821, -0.3797) | -1.3193 (-1.3339, -1.3047) | -1.5190 (-1.5384, -1.4996) | -0.7033 (-0.7075, -0.6992) | -0.6871 (-0.6910, -0.6831) | -1.1106 (-1.1209, -1.1002) | 0.0616 (0.0616, 0.0616) |
| Western Europe | -2.1207 (-2.1585, -2.0829) | -1.4089 (-1.4256, -1.3923) | -3.3584 (-3.4531, -3.2636) | -1.8998 (-1.9301, -1.8695) | -0.9671 (-0.9749, -0.9592) | -3.4843 (-3.5862, -3.3822) | -1.6820 (-1.7058, -1.6582) | -1.1183 (-1.1288, -1.1078) | -3.0288 (-3.1059, -2.9517) |

**STable 3. The projected age-standardized rates of Alzheimer's Disease in 2030 in different regions, by sexes.**

| location | Deaths | | | DALYs (Disability-Adjusted Life Years) | | | Incidence | | |
| --- | --- | --- | --- | --- | --- | --- | --- | --- | --- |
|  | Male | Female | Both | Male | Female | Both | Male | Female | Both |
| Global | 6786.66 (6401.84, 7171.47) | 6751.16 (6389.90, 7112.42) | 7210.92 (6730.65, 7691.19) | 289.55 (269.55, 309.55) | 298.85 (279.72, 317.97) | 299.92 (274.94, 324.91) | 2009.00 (1895.67, 2122.33) | 1947.26 (1843.42, 2051.10) | 2199.67 (2054.49, 2344.86) |
| High SDI | 5096.05 (4773.92, 5418.19) | 5501.98 (5207.09, 5796.87) | 4648.20 (4167.93, 5128.46) | 255.85 (236.64, 275.05) | 296.12 (276.49, 315.76) | 208.69 (181.69, 235.70) | 1426.02 (1340.76, 1511.27) | 1476.47 (1413.08, 1539.86) | 1328.72 (1168.04, 1489.41) |
| High-middle SDI | 6182.76 (5291.31, 7074.20) | 6140.39 (5369.03, 6911.74) | 6442.73 (5063.42, 7822.04) | 252.78 (206.59, 298.97) | 265.30 (224.59, 306.02) | 244.15 (173.74, 314.57) | 2034.05 (1789.44, 2278.66) | 1976.71 (1768.88, 2184.55) | 2177.52 (1779.83, 2575.21) |
| Middle SDI | 8897.50 (8426.84, 9368.16) | 8066.11 (7676.63, 8455.58) | 10481.59 (10030.08, 10933.10) | 366.15 (342.79, 389.50) | 341.87 (323.03, 360.70) | 434.37 (410.83, 457.91) | 2670.24 (2506.68, 2833.80) | 2433.72 (2279.84, 2587.60) | 3219.02 (3055.21, 3382.84) |
| Low-middle SDI | 9809.02 (9485.28, 10132.77) | 9846.71 (9451.00, 10242.41) | 9800.55 (9440.64, 10160.45) | 427.14 (412.67, 441.61) | 435.05 (415.85, 454.24) | 420.53 (405.62, 435.44) | 2598.90 (2496.82, 2700.98) | 2447.49 (2327.95, 2567.03) | 2842.88 (2720.94, 2964.81) |
| Low SDI | 16493.42 (15769.98, 17216.86) | 16895.52 (16096.44, 17694.60) | 15730.25 (14992.73, 16467.77) | 758.42 (723.56, 793.27) | 783.71 (747.00, 820.43) | 720.13 (682.26, 757.99) | 4114.06 (3934.58, 4293.54) | 4007.75 (3804.40, 4211.11) | 4231.99 (4034.98, 4429.00) |
| Andean Latin America | 4841.25 (4697.77, 4984.72) | 5203.94 (5015.47, 5392.41) | 4583.57 (4410.91, 4756.22) | 197.81 (192.53, 203.08) | 215.09 (206.43, 223.74) | 187.97 (181.53, 194.41) | 1478.75 (1425.09, 1532.41) | 1611.40 (1538.57, 1684.22) | 1367.18 (1310.71, 1423.65) |
| Australasia | 4581.69 (4256.90, 4906.48) | 5187.12 (4933.01, 5441.23) | 3338.99 (2566.57, 4111.42) | 199.96 (177.39, 222.52) | 243.90 (225.51, 262.29) | 120.69 (71.35, 170.03) | 1373.51 (1305.60, 1441.41) | 1529.09 (1481.61, 1576.57) | 1066.49 (889.38, 1243.60) |
| Caribbean | 4810.02 (4414.99, 5205.06) | 4698.36 (4435.19, 4961.53) | 5047.12 (4390.30, 5703.93) | 198.98 (180.57, 217.38) | 197.37 (185.63, 209.11) | 206.91 (174.67, 239.16) | 1575.26 (1449.89, 1700.63) | 1543.78 (1459.91, 1627.64) | 1638.10 (1434.20, 1841.99) |
| Central Asia | 6802.39 (6210.08, 7394.69) | 6650.23 (6161.77, 7138.70) | 6561.52 (5631.14, 7491.90) | 279.73 (254.99, 304.47) | 279.76 (256.31, 303.21) | 281.39 (257.89, 304.89) | 1919.42 (1715.53, 2123.31) | 1874.73 (1717.45, 2032.01) | 1682.71 (1292.82, 2072.60) |
| Central Europe | 1872.69 (330.75, 3414.64) | 1852.75 (466.56, 3238.95) | 2242.78 (84.33, 4401.23) | 45.12 (0.05, 124.39) | 57.64 (0.05, 128.90) | 38.33 (0.05, 150.56) | 597.79 (128.29, 1067.30) | 555.60 (141.53, 969.66) | 761.82 (79.21, 1444.42) |
| Central Latin America | 5713.13 (5454.63, 5971.62) | 5794.48 (5503.77, 6085.18) | 5659.25 (5383.87, 5934.63) | 222.33 (215.38, 229.28) | 228.24 (220.05, 236.42) | 217.42 (209.14, 225.69) | 1911.96 (1792.57, 2031.36) | 1917.35 (1785.70, 2049.00) | 1927.95 (1803.11, 2052.80) |
| Central Sub-Saharan Africa | 22703.53 (21500.55, 23906.51) | 22281.23 (21099.20, 23463.25) | 23415.63 (22026.59, 24804.68) | 1037.46 (984.77, 1090.15) | 1042.08 (986.50, 1097.67) | 1042.61 (983.46, 1101.76) | 5259.96 (4953.43, 5566.49) | 5042.83 (4731.47, 5354.18) | 5704.88 (5353.12, 6056.63) |
| East Asia | 10603.49 (7733.02, 13473.96) | 9265.69 (6970.27, 11561.10) | 16026.62 (10318.51, 21734.73) | 449.61 (308.17, 591.05) | 400.02 (287.70, 512.34) | 691.46 (396.82, 986.10) | 3135.23 (2297.63, 3972.83) | 2746.37 (2089.11, 3403.62) | 4755.73 (3123.08, 6388.38) |
| Eastern Europe | 1404.84 (0.05, 3014.43) | 1660.49 (84.94, 3236.03) | 337.20 (0.05, 2212.57) | 46.23 (0.05, 123.65) | 68.88 (0.05, 145.06) | 0.05 (0.05, 87.35) | 335.48 (0.05, 808.30) | 403.65 (0.05, 856.42) | 0.05 (0.05, 599.98) |
| Eastern Sub-Saharan Africa | 15299.70 (14724.70, 15874.71) | 16052.86 (15538.16, 16567.56) | 14109.52 (13438.59, 14780.44) | 694.63 (667.86, 721.40) | 732.56 (708.46, 756.66) | 633.17 (602.00, 664.35) | 3625.15 (3483.17, 3767.14) | 3740.77 (3614.28, 3867.26) | 3491.55 (3316.79, 3666.31) |
| High-income Asia Pacific | 4365.22 (4190.62, 4539.83) | 5331.95 (5156.51, 5507.38) | 2637.74 (2257.17, 3018.30) | 268.69 (256.77, 280.61) | 355.37 (341.02, 369.73) | 139.19 (121.16, 157.22) | 1100.66 (1024.20, 1177.11) | 1271.04 (1194.13, 1347.96) | 693.84 (558.54, 829.13) |
| High-income North America | 6850.69 (6670.46, 7030.92) | 7072.82 (6947.41, 7198.23) | 6293.00 (5860.31, 6725.69) | 298.34 (288.22, 308.46) | 315.79 (307.46, 324.12) | 265.67 (246.04, 285.30) | 1987.15 (1930.75, 2043.54) | 2011.40 (1974.26, 2048.55) | 1837.79 (1675.21, 2000.38) |
| Oceania | 13522.19 (12900.14, 14144.24) | 13765.81 (13193.08, 14338.53) | 13319.76 (12089.81, 14549.71) | 460.90 (430.99, 490.82) | 472.82 (448.27, 497.37) | 449.17 (375.19, 523.16) | 4228.61 (4039.74, 4417.48) | 4294.38 (4110.94, 4477.82) | 4184.36 (3855.20, 4513.52) |
| Southeast Asia | 7877.21 (7508.03, 8246.39) | 7973.55 (7634.38, 8312.73) | 7927.48 (7544.39, 8310.57) | 295.62 (276.91, 314.32) | 310.50 (295.14, 325.85) | 279.03 (256.22, 301.84) | 2193.81 (2090.68, 2296.95) | 2182.60 (2080.42, 2284.77) | 2263.44 (2163.37, 2363.50) |
| Southern Latin America | 5945.10 (5576.45, 6313.74) | 5939.34 (5490.35, 6388.34) | 6310.95 (6027.38, 6594.51) | 255.69 (233.84, 277.54) | 267.09 (241.51, 292.67) | 252.39 (237.44, 267.35) | 1925.46 (1818.05, 2032.87) | 1836.88 (1707.91, 1965.85) | 2211.47 (2104.46, 2318.47) |
| Southern Sub-Saharan Africa | 13477.61 (12428.99, 14526.24) | 12812.37 (11865.69, 13759.06) | 15620.00 (14235.86, 17004.14) | 566.10 (504.99, 627.20) | 547.19 (490.72, 603.65) | 634.90 (559.92, 709.88) | 3810.41 (3523.57, 4097.25) | 3531.80 (3278.60, 3785.00) | 4706.07 (4287.85, 5124.29) |
| Tropical Latin America | 7542.95 (7093.23, 7992.66) | 7200.64 (6827.34, 7573.94) | 8427.64 (7872.65, 8982.62) | 313.74 (285.12, 342.37) | 316.29 (295.54, 337.03) | 326.23 (286.64, 365.81) | 2121.87 (2043.55, 2200.19) | 1880.20 (1793.49, 1966.91) | 2626.11 (2555.75, 2696.48) |
| Western Europe | 4555.77 (3986.67, 5124.87) | 4904.98 (4353.66, 5456.29) | 4566.30 (3814.27, 5318.33) | 243.99 (210.10, 277.88) | 283.51 (248.78, 318.25) | 222.59 (179.75, 265.43) | 1346.47 (1184.37, 1508.56) | 1355.52 (1215.89, 1495.16) | 1422.18 (1175.90, 1668.45) |

**STable 4. Top and Buttom 10 regions of the projected EAPC of age-standardized rates of Alzheimer's Disease from 2022 to 2030.**

| Top |  |  |  |  |  |  |
| --- | --- | --- | --- | --- | --- | --- |
| Rank | DALYs (Disability-Adjusted Life Years) Country | DALYs (Disability-Adjusted Life Years) Value | Deaths Country | Deaths Value | Incidence Country | Incidence Value |
| 1 | Andean Latin America | 0.9371 (0.9298, 0.9445) | Andean Latin America | 0.7193 (0.7150, 0.7237) | Southern Latin America | 0.9995 (0.9911, 1.0079) |
| 2 | Southern Latin America | 0.7651 (0.7602, 0.7700) | Caribbean | 0.5578 (0.5552, 0.5605) | Andean Latin America | 0.9506 (0.9430, 0.9582) |
| 3 | Caribbean | 0.5931 (0.5902, 0.5961) | Southern Latin America | 0.3688 (0.3677, 0.3700) | Caribbean | 0.7553 (0.7505, 0.7600) |
| 4 | Central Latin America | 0.5100 (0.5078, 0.5122) | Central Latin America | 0.3524 (0.3513, 0.3534) | Oceania | 0.5631 (0.5604, 0.5657) |
| 5 | Oceania | 0.3937 (0.3924, 0.3951) | Eastern Sub-Saharan Africa | 0.1719 (0.1717, 0.1722) | Central Latin America | 0.5536 (0.5510, 0.5562) |
| 6 | High-income North America | 0.2162 (0.2158, 0.2166) | Central Sub-Saharan Africa | 0.0273 (0.0273, 0.0273) | High-income North America | 0.5365 (0.5340, 0.5389) |
| 7 | Southern Sub-Saharan Africa | 0.0033 (0.0033, 0.0033) | Oceania | -0.5430 (-0.5455, -0.5405) | Southern Sub-Saharan Africa | 0.0592 (0.0592, 0.0593) |
| 8 | Eastern Sub-Saharan Africa | -0.0259 (-0.0259, -0.0259) | High-income North America | -0.5813 (-0.5841, -0.5784) | Eastern Sub-Saharan Africa | -0.3794 (-0.3806, -0.3782) |
| 9 | Central Sub-Saharan Africa | -0.2830 (-0.2837, -0.2823) | Southern Sub-Saharan Africa | -0.6755 (-0.6794, -0.6717) | Tropical Latin America | -0.6871 (-0.6910, -0.6831) |
| 10 | Tropical Latin America | -1.0657 (-1.0753, -1.0562) | High-income Asia Pacific | -0.7750 (-0.7801, -0.7700) | Central Sub-Saharan Africa | -0.7369 (-0.7414, -0.7323) |
|  |  |  |  |  |  |  |
|  |  |  |  |  |  |  |
| Bottom |  |  |  |  |  |  |
| Rank | DALYs (Disability-Adjusted Life Years) Country | DALYs (Disability-Adjusted Life Years) Value | Deaths Country | Deaths Value | Incidence Country | Incidence Value |
| 1 | Eastern Europe | -16.3071 (-18.6030, -13.9464) | Eastern Europe | -19.0569 (-22.2295, -15.7549) | Eastern Europe | -18.4027 (-21.3524, -15.3424) |
| 2 | Central Europe | -12.0323 (-13.2652, -10.7819) | Central Europe | -17.4959 (-20.1515, -14.7520) | Central Europe | -11.7452 (-12.9190, -10.5555) |
| 3 | East Asia | -2.7709 (-2.8354, -2.7064) | East Asia | -3.0900 (-3.1702, -3.0097) | East Asia | -2.9029 (-2.9737, -2.8321) |
| 4 | Southeast Asia | -2.2938 (-2.3379, -2.2496) | Southeast Asia | -3.0312 (-3.1084, -2.9540) | Central Asia | -2.8444 (-2.9123, -2.7764) |
| 5 | Western Europe | -2.1207 (-2.1585, -2.0829) | Australasia | -2.6780 (-2.7383, -2.6178) | High-income Asia Pacific | -2.7236 (-2.7859, -2.6612) |
| 6 | High-income Asia Pacific | -2.0629 (-2.0986, -2.0271) | Western Europe | -1.8998 (-1.9301, -1.8695) | Southeast Asia | -2.4581 (-2.5088, -2.4073) |
| 7 | Central Asia | -1.8372 (-1.8655, -1.8088) | Central Asia | -1.8902 (-1.9202, -1.8602) | Western Europe | -1.6820 (-1.7058, -1.6582) |
| 8 | Australasia | -1.6019 (-1.6234, -1.5804) | Tropical Latin America | -1.3193 (-1.3339, -1.3047) | Australasia | -0.8525 (-0.8586, -0.8464) |
| 9 | Tropical Latin America | -1.0657 (-1.0753, -1.0562) | High-income Asia Pacific | -0.7750 (-0.7801, -0.7700) | Central Sub-Saharan Africa | -0.7369 (-0.7414, -0.7323) |
| 10 | Central Sub-Saharan Africa | -0.2830 (-0.2837, -0.2823) | Southern Sub-Saharan Africa | -0.6755 (-0.6794, -0.6717) | Tropical Latin America | -0.6871 (-0.6910, -0.6831) |

**STable 5. Top and Buttom 10 regions of the projected age-standardized rates of Alzheimer's Disease in 2030.**

| Top |  |  |  |  |  |  |
| --- | --- | --- | --- | --- | --- | --- |
| Rank | DALYs (Disability-Adjusted Life Years) Country | DALYs (Disability-Adjusted Life Years) Value | Deaths Country | Deaths Value | Incidence Country | Incidence Value |
| 1 | Central Sub-Saharan Africa | 22703.53 (21500.55, 23906.51) | Central Sub-Saharan Africa | 1037.46 (984.77, 1090.15) | Central Sub-Saharan Africa | 5259.96 (4953.43, 5566.49) |
| 2 | Eastern Sub-Saharan Africa | 15299.70 (14724.70, 15874.71) | Eastern Sub-Saharan Africa | 694.63 (667.86, 721.40) | Oceania | 4228.61 (4039.74, 4417.48) |
| 3 | Oceania | 13522.19 (12900.14, 14144.24) | Southern Sub-Saharan Africa | 566.10 (504.99, 627.20) | Southern Sub-Saharan Africa | 3810.41 (3523.57, 4097.25) |
| 4 | Southern Sub-Saharan Africa | 13477.61 (12428.99, 14526.24) | Oceania | 460.90 (430.99, 490.82) | Eastern Sub-Saharan Africa | 3625.15 (3483.17, 3767.14) |
| 5 | East Asia | 10603.49 (7733.02, 13473.96) | East Asia | 449.61 (308.17, 591.05) | East Asia | 3135.23 (2297.63, 3972.83) |
| 6 | Southeast Asia | 7877.21 (7508.03, 8246.39) | Tropical Latin America | 313.74 (285.12, 342.37) | Southeast Asia | 2193.81 (2090.68, 2296.95) |
| 7 | Tropical Latin America | 7542.95 (7093.23, 7992.66) | High-income North America | 298.34 (288.22, 308.46) | Tropical Latin America | 2121.87 (2043.55, 2200.19) |
| 8 | High-income North America | 6850.69 (6670.46, 7030.92) | Southeast Asia | 295.62 (276.91, 314.32) | High-income North America | 1987.15 (1930.75, 2043.54) |
| 9 | Central Asia | 6802.39 (6210.08, 7394.69) | Central Asia | 279.73 (254.99, 304.47) | Southern Latin America | 1925.46 (1818.05, 2032.87) |
| 10 | Southern Latin America | 5945.10 (5576.45, 6313.74) | High-income Asia Pacific | 268.69 (256.77, 280.61) | Central Asia | 1919.42 (1715.53, 2123.31) |
|  |  |  |  |  |  |  |
|  |  |  |  |  |  |  |
| Bottom |  |  |  |  |  |  |
| Rank | DALYs (Disability-Adjusted Life Years) Country | DALYs (Disability-Adjusted Life Years) Value | Deaths Country | Deaths Value | Incidence Country | Incidence Value |
| 1 | Eastern Europe | 1404.84 (0.05, 3014.43) | Central Europe | 45.12 (0.05, 124.39) | Eastern Europe | 335.48 (0.05, 808.30) |
| 2 | Central Europe | 1872.69 (330.75, 3414.64) | Eastern Europe | 46.23 (0.05, 123.65) | Central Europe | 597.79 (128.29, 1067.30) |
| 3 | High-income Asia Pacific | 4365.22 (4190.62, 4539.83) | Andean Latin America | 197.81 (192.53, 203.08) | High-income Asia Pacific | 1100.66 (1024.20, 1177.11) |
| 4 | Western Europe | 4555.77 (3986.67, 5124.87) | Caribbean | 198.98 (180.57, 217.38) | Western Europe | 1346.47 (1184.37, 1508.56) |
| 5 | Australasia | 4581.69 (4256.90, 4906.48) | Australasia | 199.96 (177.39, 222.52) | Australasia | 1373.51 (1305.60, 1441.41) |
| 6 | Caribbean | 4810.02 (4414.99, 5205.06) | Central Latin America | 222.33 (215.38, 229.28) | Andean Latin America | 1478.75 (1425.09, 1532.41) |
| 7 | Andean Latin America | 4841.25 (4697.77, 4984.72) | Western Europe | 243.99 (210.10, 277.88) | Caribbean | 1575.26 (1449.89, 1700.63) |
| 8 | Central Latin America | 5713.13 (5454.63, 5971.62) | Southern Latin America | 255.69 (233.84, 277.54) | Central Latin America | 1911.96 (1792.57, 2031.36) |
| 9 | Southern Latin America | 5945.10 (5576.45, 6313.74) | High-income Asia Pacific | 268.69 (256.77, 280.61) | Central Asia | 1919.42 (1715.53, 2123.31) |
| 10 | Central Asia | 6802.39 (6210.08, 7394.69) | Central Asia | 279.73 (254.99, 304.47) | Southern Latin America | 1925.46 (1818.05, 2032.87) |

**STable 6. The projected EAPC of Alzheimer's Disease from 1990 to 2021 and from 2022 to 2030 in different countries.**

| location | DALYs (Disability-Adjusted Life Years) | | Deaths | | Incidence | |
| --- | --- | --- | --- | --- | --- | --- |
|  | 1990-2021 | 2022-2030 | 1990-2021 | 2022-2030 | 1990-2021 | 2022-2030 |
| Afghanistan | -0.5939 (-0.7769, -0.4106) | -0.5844 (-0.5872, -0.5815) | -0.4656 (-0.7081, -0.2226) | -0.3232 (-0.3240, -0.3223) | -0.5242 (-0.7087, -0.3393) | -0.7985 (-0.8039, -0.7932) |
| Albania | 0.5881 (0.3955, 0.7811) | -0.2246 (-0.2250, -0.2242) | 1.0028 (0.8322, 1.1737) | 0.3048 (0.3041, 0.3056) | 0.7039 (0.4934, 0.9149) | -0.4952 (-0.4972, -0.4931) |
| Algeria | -3.6104 (-4.4252, -2.7886) | -13.5350 (-15.1016, -11.9394) | -3.6711 (-4.5941, -2.7393) | -11.5381 (-12.6704, -10.3912) | -3.4370 (-4.2210, -2.6465) | -14.0714 (-15.7675, -12.3411) |
| American Samoa | -1.9265 (-2.0725, -1.7803) | -1.6254 (-1.6475, -1.6032) | -1.7877 (-1.9708, -1.6042) | -2.4166 (-2.4656, -2.3676) | -1.8338 (-2.0211, -1.6461) | -0.9548 (-0.9625, -0.9472) |
| Andorra | -5.8354 (-6.9409, -4.7166) | 5.1059 (4.8866, 5.3256) | -5.6695 (-6.9045, -4.4182) | 4.1747 (4.0283, 4.3214) | -5.9625 (-6.9914, -4.9222) | 5.8660 (5.5764, 6.1564) |
| Angola | -0.3771 (-0.5011, -0.2529) | -0.7870 (-0.7922, -0.7818) | -0.0950 (-0.2262, 0.0363) | -0.4956 (-0.4977, -0.4936) | -0.7723 (-0.9192, -0.6251) | -1.3305 (-1.3454, -1.3157) |
| Antigua and Barbuda | 1.2830 (1.0350, 1.5316) | 1.4749 (1.4566, 1.4932) | 0.9911 (0.7244, 1.2586) | 1.0035 (0.9950, 1.0119) | 1.3813 (1.1373, 1.6259) | 1.7803 (1.7537, 1.8069) |
| Argentina | -1.7039 (-1.9136, -1.4937) | 1.8937 (1.8636, 1.9238) | -1.4975 (-1.7002, -1.2944) | 1.4575 (1.4396, 1.4753) | -1.8984 (-2.1222, -1.6740) | 2.2495 (2.2070, 2.2920) |
| Armenia | 1.2559 (0.3938, 2.1254) | -85.4069 (-92.8028, -70.4109) | 1.9455 (1.1526, 2.7446) | -75.6245 (-84.8331, -60.8247) | 0.9956 (0.0666, 1.9333) | -81.9912 (-90.0409, -67.4353) |
| Australia | -2.0097 (-2.1221, -1.8971) | -1.6253 (-1.6475, -1.6031) | -1.5781 (-1.6780, -1.4782) | -2.7596 (-2.8236, -2.6956) | -2.5138 (-2.6414, -2.3861) | -0.8229 (-0.8286, -0.8173) |
| Austria | -2.2032 (-2.5820, -1.8230) | -3.9006 (-4.0284, -3.7725) | -2.0281 (-2.4898, -1.5642) | -4.2947 (-4.4498, -4.1394) | -2.3733 (-2.7019, -2.0436) | -3.8880 (-4.0150, -3.7608) |
| Azerbaijan | -0.4046 (-1.0207, 0.2153) | 1.7191 (1.6943, 1.7439) | -0.8021 (-1.5814, -0.0165) | 2.5513 (2.4966, 2.6059) | -0.2631 (-0.8659, 0.3434) | 0.9201 (0.9130, 0.9272) |
| Bahamas | -0.5232 (-0.7254, -0.3205) | 0.7033 (0.6992, 0.7075) | -0.6166 (-0.7957, -0.4371) | 0.5436 (0.5411, 0.5461) | -0.5088 (-0.7312, -0.2860) | 0.8060 (0.8005, 0.8114) |
| Bahrain | 0.6879 (-0.0017, 1.3822) | -87.2833 (-94.6637, -69.6952) | 0.9640 (0.2372, 1.6960) | -61.5585 (-78.9395, -29.8334) | 0.8420 (0.1146, 1.5747) | -86.9348 (-93.8452, -72.2653) |
| Bangladesh | 1.6103 (1.1051, 2.1180) | -7.8685 (-8.3912, -7.3428) | 1.8011 (1.3508, 2.2534) | -5.3421 (-5.5822, -5.1013) | 1.3952 (0.8768, 1.9163) | -9.7633 (-10.5707, -8.9485) |
| Barbados | -0.9999 (-1.6143, -0.3817) | -2.9263 (-2.9982, -2.8543) | -1.1644 (-1.7989, -0.5258) | -4.0420 (-4.1794, -3.9045) | -1.0849 (-1.7147, -0.4511) | -2.8967 (-2.9671, -2.8261) |
| Belarus | -0.1962 (-0.5034, 0.1119) | -8.6574 (-9.2910, -8.0194) | 0.0178 (-0.3323, 0.3692) | -10.2716 (-11.1663, -9.3680) | -0.2125 (-0.5657, 0.1420) | -10.3760 (-11.2892, -9.4535) |
| Belgium | -2.0802 (-2.3517, -1.8081) | -4.5852 (-4.7620, -4.4081) | -1.6953 (-2.0204, -1.3692) | -4.8844 (-5.0850, -4.6833) | -2.4026 (-2.6583, -2.1462) | -3.9661 (-4.0983, -3.8337) |
| Belize | 0.6017 (0.3366, 0.8675) | -0.4044 (-0.4057, -0.4030) | 0.5327 (0.2934, 0.7727) | -1.3368 (-1.3518, -1.3218) | 0.5508 (0.2749, 0.8274) | -0.2824 (-0.2830, -0.2817) |
| Benin | -0.0187 (-0.1677, 0.1305) | -0.5783 (-0.5811, -0.5755) | 0.0852 (-0.0840, 0.2546) | -0.8398 (-0.8457, -0.8338) | -0.1541 (-0.2877, -0.0205) | 0.0841 (0.0840, 0.0841) |
| Bermuda | -2.1565 (-2.2478, -2.0652) | -1.5235 (-1.5430, -1.5040) | -1.9724 (-2.1152, -1.8294) | -1.0327 (-1.0417, -1.0238) | -2.1805 (-2.2605, -2.1005) | -1.6665 (-1.6898, -1.6431) |
| Bhutan | -1.4909 (-1.7600, -1.2210) | -1.8416 (-1.8701, -1.8132) | -0.7450 (-1.0519, -0.4371) | -1.9072 (-1.9377, -1.8767) | -2.1389 (-2.3849, -1.8922) | -1.8771 (-1.9066, -1.8475) |
| Bolivia (Plurinational State of) | -0.8680 (-0.9752, -0.7606) | -0.4626 (-0.4644, -0.4608) | -0.7976 (-0.9166, -0.6785) | -1.4412 (-1.4586, -1.4237) | -0.9045 (-1.0191, -0.7897) | -0.2075 (-0.2078, -0.2071) |
| Bosnia and Herzegovina | -0.6984 (-1.3204, -0.0725) | -3.6429 (-3.7544, -3.5313) | -0.6176 (-1.4514, 0.2233) | -2.6342 (-2.6925, -2.5759) | -0.5171 (-1.1367, 0.1065) | -4.8306 (-5.0268, -4.6339) |
| Botswana | -2.8631 (-3.0478, -2.6781) | -2.2613 (-2.3043, -2.2184) | -2.8085 (-2.9869, -2.6297) | -2.6302 (-2.6883, -2.5721) | -2.8857 (-3.0827, -2.6884) | -1.7104 (-1.7349, -1.6858) |
| Brazil | -2.3405 (-2.6017, -2.0787) | -1.1951 (-1.2071, -1.1832) | -2.4170 (-2.7242, -2.1089) | -1.4694 (-1.4875, -1.4512) | -2.3577 (-2.5934, -2.1215) | -0.7961 (-0.8014, -0.7908) |
| Brunei Darussalam | 2.0260 (1.7549, 2.2978) | 0.8187 (0.8131, 0.8243) | 2.0944 (1.7885, 2.4012) | 0.4656 (0.4638, 0.4674) | 1.7792 (1.5270, 2.0319) | 1.2943 (1.2803, 1.3084) |
| Bulgaria | -7.6307 (-8.8302, -6.4155) | 0.0000 (0.0000, 0.0000) | -7.2524 (-8.6490, -5.8346) | 0.0000 (0.0000, 0.0000) | -7.8649 (-8.9823, -6.7339) | 0.0000 (0.0000, 0.0000) |
| Burkina Faso | -2.1500 (-2.4251, -1.8742) | -0.4738 (-0.4757, -0.4719) | -2.3457 (-2.6185, -2.0721) | -0.5422 (-0.5447, -0.5397) | -1.9947 (-2.2300, -1.7587) | -0.2395 (-0.2400, -0.2390) |
| Burundi | -1.4267 (-1.6620, -1.1908) | -0.5213 (-0.5236, -0.5190) | -1.4958 (-1.7373, -1.2537) | -0.7708 (-0.7758, -0.7658) | -1.7662 (-2.0130, -1.5188) | -0.5626 (-0.5653, -0.5600) |
| Cabo Verde | -0.5076 (-0.8874, -0.1263) | 1.1404 (1.1295, 1.1513) | -0.3323 (-0.7170, 0.0538) | 1.4355 (1.4182, 1.4528) | -0.7211 (-1.1330, -0.3076) | 0.3168 (0.3159, 0.3176) |
| Cambodia | 0.0395 (-0.0320, 0.1111) | 0.5294 (0.5271, 0.5318) | 0.4018 (0.3089, 0.4949) | 0.6849 (0.6810, 0.6888) | -0.4997 (-0.5651, -0.4343) | 0.4226 (0.4211, 0.4241) |
| Cameroon | 0.2221 (-0.0167, 0.4613) | -2.9098 (-2.9809, -2.8386) | 0.3277 (0.1060, 0.5499) | -3.0487 (-3.1268, -2.9706) | 0.3027 (0.0121, 0.5941) | -2.6163 (-2.6738, -2.5588) |
| Canada | -1.5550 (-1.6701, -1.4399) | -1.3218 (-1.3365, -1.3072) | -1.0035 (-1.0961, -0.9108) | -0.9955 (-1.0038, -0.9871) | -1.9908 (-2.1264, -1.8550) | -1.5798 (-1.6007, -1.5588) |
| Central African Republic | 0.2445 (-0.1069, 0.5972) | 1.8069 (1.7795, 1.8343) | 0.5844 (0.1824, 0.9880) | 2.4102 (2.3614, 2.4590) | 0.1365 (-0.2285, 0.5029) | 1.4866 (1.4681, 1.5052) |
| Chad | 0.1148 (-0.0441, 0.2740) | 1.1125 (1.1021, 1.1229) | 0.1157 (-0.0455, 0.2772) | 1.3146 (1.3001, 1.3291) | -0.2227 (-0.3808, -0.0644) | 0.9478 (0.9403, 0.9554) |
| Chile | -1.8678 (-2.2702, -1.4637) | -2.3855 (-2.4333, -2.3377) | -1.9126 (-2.3898, -1.4331) | -2.5142 (-2.5673, -2.4611) | -1.8327 (-2.2106, -1.4533) | -2.6735 (-2.7335, -2.6134) |
| China | -3.8777 (-4.0894, -3.6656) | -3.3014 (-3.3929, -3.2097) | -4.2182 (-4.4581, -3.9777) | -3.8098 (-3.9318, -3.6877) | -3.4016 (-3.6121, -3.1906) | -3.4693 (-3.5704, -3.3681) |
| Colombia | -1.3811 (-1.4658, -1.2964) | -0.9030 (-0.9099, -0.8962) | -0.9870 (-1.1102, -0.8637) | -0.2946 (-0.2953, -0.2938) | -1.5345 (-1.6169, -1.4519) | -1.4315 (-1.4487, -1.4143) |
| Comoros | -1.6360 (-1.6884, -1.5836) | -1.5290 (-1.5486, -1.5094) | -1.4102 (-1.4729, -1.3474) | -1.4507 (-1.4684, -1.4330) | -2.0572 (-2.1033, -2.0111) | -1.8070 (-1.8344, -1.7796) |
| Congo | -3.5489 (-3.9287, -3.1677) | -2.1238 (-2.1617, -2.0859) | -3.6110 (-4.0282, -3.1919) | -2.5887 (-2.6450, -2.5324) | -3.5423 (-3.8841, -3.1993) | -1.9242 (-1.9553, -1.8931) |
| Cook Islands | -2.0854 (-2.1815, -1.9892) | -3.8474 (-3.9717, -3.7228) | -2.1460 (-2.2755, -2.0164) | -3.5855 (-3.6935, -3.4774) | -1.7982 (-1.8894, -1.7069) | -3.4727 (-3.5740, -3.3712) |
| Costa Rica | -0.8671 (-0.9718, -0.7622) | 1.0764 (1.0667, 1.0862) | -0.5960 (-0.6982, -0.4937) | 0.8811 (0.8746, 0.8876) | -0.9943 (-1.1157, -0.8727) | 1.0330 (1.0240, 1.0419) |
| Côte d'Ivoire | -1.0177 (-1.2096, -0.8254) | -1.7638 (-1.7899, -1.7377) | -0.7632 (-0.9866, -0.5394) | -2.5739 (-2.6296, -2.5183) | -1.1689 (-1.3810, -0.9563) | -1.5494 (-1.5695, -1.5292) |
| Croatia | -0.4154 (-0.9289, 0.1007) | -5.7574 (-6.0364, -5.4775) | -0.0707 (-0.6500, 0.5120) | -6.8643 (-7.2615, -6.4654) | -0.4218 (-0.9654, 0.1248) | -5.6008 (-5.8648, -5.3360) |
| Cuba | -2.9799 (-3.8528, -2.0990) | 4.9480 (4.7421, 5.1542) | -3.0865 (-4.0914, -2.0710) | 5.5482 (5.2892, 5.8078) | -3.0390 (-3.8731, -2.1977) | 5.0410 (4.8273, 5.2551) |
| Cyprus | -6.4140 (-7.9587, -4.8433) | 12.5532 (11.2142, 13.9082) | -6.4106 (-8.0842, -4.7066) | 12.8393 (11.4379, 14.2584) | -6.1325 (-7.5959, -4.6459) | 12.1836 (10.9232, 13.4584) |
| Czechia | -1.7494 (-2.2648, -1.2312) | -7.1307 (-7.5594, -6.6999) | -1.7463 (-2.4008, -1.0875) | -15.4358 (-17.4863, -13.3343) | -1.8094 (-2.2982, -1.3181) | -5.7775 (-6.0585, -5.4956) |
| Democratic People's Republic of Korea | 0.0862 (-1.1561, 1.3440) | 2.6441 (2.5854, 2.7028) | 0.5262 (-0.8097, 1.8801) | 3.5092 (3.4058, 3.6128) | -0.1718 (-1.3893, 1.0608) | 2.5584 (2.5034, 2.6134) |
| Democratic Republic of the Congo | -2.1745 (-2.5138, -1.8340) | -0.3235 (-0.3244, -0.3226) | -1.7452 (-2.1865, -1.3019) | 0.1584 (0.1581, 0.1586) | -2.6245 (-2.9765, -2.2712) | -0.8871 (-0.8937, -0.8805) |
| Denmark | -1.2745 (-1.4732, -1.0755) | -0.1999 (-0.2002, -0.1995) | -1.0404 (-1.1717, -0.9089) | -0.8916 (-0.8983, -0.8849) | -1.8886 (-2.1694, -1.6070) | 0.5492 (0.5467, 0.5518) |
| Djibouti | 0.0706 (-0.0378, 0.1791) | 0.7674 (0.7624, 0.7723) | 0.2766 (0.1343, 0.4191) | 1.2072 (1.1950, 1.2194) | -0.2373 (-0.3273, -0.1473) | 0.3669 (0.3658, 0.3681) |
| Dominica | -1.3716 (-1.5098, -1.2333) | 0.5756 (0.5728, 0.5784) | -1.5196 (-1.6255, -1.4136) | 0.2302 (0.2298, 0.2307) | -1.3955 (-1.5498, -1.2410) | 0.6348 (0.6314, 0.6382) |
| Dominican Republic | -1.8316 (-2.3221, -1.3386) | -2.1246 (-2.1625, -2.0867) | -1.7507 (-2.2426, -1.2563) | -2.1731 (-2.2128, -2.1334) | -1.9351 (-2.4319, -1.4357) | -2.3657 (-2.4127, -2.3186) |
| Ecuador | -0.4330 (-0.7983, -0.0663) | 2.7338 (2.6710, 2.7966) | -0.4104 (-0.8669, 0.0481) | 2.9446 (2.8718, 3.0175) | -0.3866 (-0.7466, -0.0254) | 2.8810 (2.8113, 2.9508) |
| Egypt | -1.8835 (-2.3563, -1.4085) | 0.4107 (0.4093, 0.4121) | -2.7750 (-3.3016, -2.2457) | -0.1831 (-0.1833, -0.1828) | -1.4245 (-1.8708, -0.9761) | 0.6802 (0.6764, 0.6841) |
| El Salvador | -0.5388 (-0.6189, -0.4586) | -0.0857 (-0.0858, -0.0857) | -0.0909 (-0.1706, -0.0112) | -0.6290 (-0.6323, -0.6257) | -0.5500 (-0.6129, -0.4870) | 0.0524 (0.0524, 0.0524) |
| Equatorial Guinea | -1.8332 (-1.9318, -1.7345) | -0.4316 (-0.4332, -0.4300) | -1.6652 (-1.7687, -1.5616) | -0.2052 (-0.2055, -0.2048) | -2.0983 (-2.2220, -1.9744) | -0.4663 (-0.4682, -0.4645) |
| Eritrea | 0.0236 (-0.2099, 0.2577) | -1.4193 (-1.4362, -1.4024) | 0.6513 (0.3719, 0.9314) | -0.2785 (-0.2792, -0.2779) | -0.5729 (-0.7585, -0.3871) | -2.2347 (-2.2766, -2.1927) |
| Estonia | -1.5700 (-1.8928, -1.2461) | -20.7481 (-24.5402, -16.7655) | -1.3308 (-1.6807, -0.9796) | -17.3097 (-19.9071, -14.6281) | -1.7248 (-2.0732, -1.3751) | -27.3273 (-34.1493, -19.7985) |
| Eswatini | 2.9475 (2.7951, 3.1001) | 3.2696 (3.1798, 3.3595) | 3.1013 (2.9016, 3.3013) | 4.0242 (3.8881, 4.1605) | 2.7023 (2.5440, 2.8609) | 3.1176 (3.0360, 3.1994) |
| Ethiopia | -4.6441 (-4.7916, -4.4963) | -4.0760 (-4.2156, -3.9362) | -4.3950 (-4.5945, -4.1952) | -3.5150 (-3.6188, -3.4111) | -4.8924 (-5.0336, -4.7511) | -5.0972 (-5.3158, -4.8781) |
| Fiji | 0.4498 (-0.1133, 1.0161) | 2.0185 (1.9843, 2.0528) | 0.4797 (-0.2543, 1.2192) | 3.1281 (3.0459, 3.2104) | 0.5926 (0.0669, 1.1210) | 1.7600 (1.7340, 1.7860) |
| Finland | -1.9268 (-2.1839, -1.6690) | -2.6686 (-2.7285, -2.6088) | -1.6281 (-1.9402, -1.3151) | -3.3806 (-3.4766, -3.2845) | -2.2846 (-2.5071, -2.0616) | -1.7766 (-1.8031, -1.7501) |
| France | -1.6941 (-1.9314, -1.4562) | -5.1042 (-5.3234, -4.8846) | -1.3336 (-1.6458, -1.0204) | -4.4460 (-4.6122, -4.2796) | -1.9985 (-2.2473, -1.7490) | -5.6469 (-5.9153, -5.3777) |
| Gabon | -0.1006 (-0.2550, 0.0539) | -0.1548 (-0.1550, -0.1546) | -0.0681 (-0.2269, 0.0909) | -0.6139 (-0.6170, -0.6107) | -0.1208 (-0.2782, 0.0368) | -0.1507 (-0.1508, -0.1505) |
| Gambia | -1.3854 (-1.6041, -1.1663) | 0.3687 (0.3676, 0.3699) | -1.2810 (-1.5554, -1.0059) | 0.7772 (0.7721, 0.7823) | -1.5556 (-1.7593, -1.3515) | -0.9857 (-0.9939, -0.9775) |
| Georgia | -1.4052 (-1.8018, -1.0070) | 0.8793 (0.8728, 0.8858) | -1.0511 (-1.5275, -0.5724) | 1.5108 (1.4916, 1.5300) | -1.5673 (-1.9684, -1.1645) | -0.1621 (-0.1623, -0.1618) |
| Germany | -1.0403 (-1.4220, -0.6572) | -1.2101 (-1.2224, -1.1978) | -1.0294 (-1.4780, -0.5787) | 0.0895 (0.0894, 0.0895) | -1.0887 (-1.4715, -0.7044) | -3.3503 (-3.4446, -3.2559) |
| Ghana | -0.2363 (-0.3043, -0.1683) | 0.2334 (0.2329, 0.2338) | -0.0254 (-0.1146, 0.0640) | 0.4398 (0.4382, 0.4415) | -0.5930 (-0.6619, -0.5240) | -0.5073 (-0.5095, -0.5052) |
| Greece | -2.0165 (-2.6806, -1.3478) | -20.5512 (-24.2679, -16.6521) | -1.6814 (-2.3764, -0.9815) | -19.9498 (-23.4416, -16.2987) | -2.2194 (-2.9017, -1.5324) | -21.9870 (-26.2734, -17.4514) |
| Greenland | -1.7964 (-2.2598, -1.3308) | -5.4557 (-5.7062, -5.2046) | -2.1244 (-2.7643, -1.4803) | -6.3619 (-6.7028, -6.0197) | -1.6707 (-2.1047, -1.2349) | -5.4816 (-5.7345, -5.2281) |
| Grenada | 1.8994 (1.5174, 2.2829) | 3.3067 (3.2148, 3.3986) | 1.0237 (0.7250, 1.3232) | 3.1327 (3.0503, 3.2152) | 2.0558 (1.6215, 2.4919) | 3.3295 (3.2364, 3.4227) |
| Guam | -6.6336 (-7.5361, -5.7222) | 2.5807 (2.5247, 2.6366) | -6.4122 (-7.5420, -5.2687) | 2.3780 (2.3305, 2.4255) | -6.5474 (-7.3816, -5.7056) | 2.2959 (2.2517, 2.3402) |
| Guatemala | -0.1121 (-0.8795, 0.6612) | -78.3742 (-90.6146, -50.1698) | 0.4189 (-0.4277, 1.2728) | -70.8300 (-82.4232, -51.5902) | -0.2602 (-1.0147, 0.5001) | -75.1766 (-88.3065, -47.3039) |
| Guinea | -1.1177 (-1.5858, -0.6474) | 0.2899 (0.2892, 0.2906) | -0.7273 (-1.2647, -0.1870) | -0.9246 (-0.9318, -0.9174) | -1.4116 (-1.8796, -0.9414) | 0.1135 (0.1134, 0.1136) |
| Guinea-Bissau | -0.4255 (-0.8020, -0.0476) | 2.0751 (2.0390, 2.1113) | -0.5022 (-0.8986, -0.1041) | 2.3553 (2.3087, 2.4019) | -0.5802 (-0.9548, -0.2042) | 1.9129 (1.8822, 1.9436) |
| Guyana | 0.2807 (-1.1609, 1.7433) | -8.0788 (-8.6300, -7.5243) | 0.4590 (-1.2426, 2.1899) | -69.9293 (-80.4358, -53.7805) | 0.1146 (-1.2655, 1.5140) | -4.1650 (-4.3108, -4.0190) |
| Haiti | -1.2994 (-1.3680, -1.2306) | -0.7723 (-0.7773, -0.7673) | -1.1638 (-1.2401, -1.0874) | -1.1089 (-1.1192, -1.0986) | -1.4218 (-1.4936, -1.3500) | -0.9399 (-0.9473, -0.9325) |
| Honduras | 1.0924 (0.5838, 1.6035) | -0.1230 (-0.1231, -0.1228) | 1.2153 (0.6262, 1.8079) | -0.3487 (-0.3497, -0.3476) | 0.7990 (0.2981, 1.3023) | 0.2122 (0.2118, 0.2126) |
| Hungary | -2.8881 (-3.1258, -2.6498) | -11.1296 (-12.1820, -10.0645) | -2.9621 (-3.2764, -2.6467) | -14.8341 (-16.7238, -12.9015) | -2.9478 (-3.1630, -2.7321) | -11.4788 (-12.5993, -10.3440) |
| Iceland | -0.7664 (-0.9036, -0.6290) | -2.2606 (-2.3035, -2.2177) | -0.2772 (-0.4117, -0.1425) | -2.7627 (-2.8268, -2.6985) | -1.1632 (-1.3094, -1.0167) | -1.4605 (-1.4784, -1.4426) |
| India | -0.8732 (-1.1989, -0.5464) | 0.6691 (0.6654, 0.6729) | -0.5317 (-0.9343, -0.1275) | 0.8319 (0.8261, 0.8377) | -1.4616 (-1.7845, -1.1376) | 0.3790 (0.3778, 0.3802) |
| Indonesia | 0.8782 (0.7304, 1.0262) | 1.3857 (1.3696, 1.4019) | 1.1401 (0.9499, 1.3306) | 1.2788 (1.2650, 1.2925) | 0.5381 (0.3623, 0.7142) | 1.2062 (1.1939, 1.2184) |
| Iran (Islamic Republic of) | -2.2691 (-3.3425, -1.1839) | 1.4292 (1.4121, 1.4463) | -1.2915 (-2.5026, -0.0654) | 2.0293 (1.9948, 2.0639) | -2.3786 (-3.4494, -1.2959) | 1.5331 (1.5134, 1.5529) |
| Iraq | 1.3177 (1.2136, 1.4218) | 2.3769 (2.3295, 2.4243) | 1.1056 (0.9963, 1.2150) | 2.4293 (2.3798, 2.4789) | 1.4690 (1.3728, 1.5652) | 2.3504 (2.3041, 2.3968) |
| Ireland | -2.8986 (-3.1245, -2.6722) | -0.8317 (-0.8375, -0.8259) | -2.8437 (-3.0721, -2.6147) | -1.1151 (-1.1255, -1.1046) | -3.0731 (-3.3020, -2.8436) | -0.6867 (-0.6906, -0.6827) |
| Israel | -3.6798 (-4.0175, -3.3409) | -1.8776 (-1.9072, -1.8480) | -3.5476 (-3.9184, -3.1754) | -2.6127 (-2.6700, -2.5554) | -3.8900 (-4.2261, -3.5528) | -1.1187 (-1.1292, -1.1082) |
| Italy | -2.2688 (-2.5699, -1.9668) | -3.6395 (-3.7508, -3.5281) | -2.0690 (-2.4298, -1.7069) | -4.6367 (-4.8175, -4.4556) | -2.0825 (-2.4199, -1.7439) | 0.4891 (0.4871, 0.4911) |
| Jamaica | -0.7998 (-1.0104, -0.5887) | 1.0026 (0.9942, 1.0110) | 0.0224 (-0.1801, 0.2254) | 0.6232 (0.6200, 0.6265) | -1.1850 (-1.4010, -0.9685) | 1.0552 (1.0459, 1.0646) |
| Japan | -2.4276 (-2.7054, -2.1489) | -1.5103 (-1.5295, -1.4912) | -1.8106 (-2.1980, -1.4217) | -0.2391 (-0.2396, -0.2386) | -2.6342 (-2.8387, -2.4293) | -2.1169 (-2.1545, -2.0793) |
| Jordan | 0.3655 (0.1962, 0.5351) | 1.2505 (1.2374, 1.2636) | 0.1890 (0.0492, 0.3290) | 1.5699 (1.5492, 1.5906) | 0.7506 (0.5922, 0.9093) | 1.2106 (1.1983, 1.2229) |
| Kazakhstan | 1.9954 (1.7948, 2.1964) | 0.1640 (0.1637, 0.1642) | 2.1902 (1.9949, 2.3859) | 0.3807 (0.3794, 0.3819) | 2.1559 (1.9073, 2.4050) | -0.6018 (-0.6048, -0.5988) |
| Kenya | 0.2403 (-0.1949, 0.6774) | 2.7980 (2.7323, 2.8638) | 0.1254 (-0.3750, 0.6283) | 2.7558 (2.6921, 2.8197) | -0.1442 (-0.5549, 0.2682) | 2.6056 (2.5486, 2.6626) |
| Kiribati | 0.2868 (0.1177, 0.4562) | 0.4251 (0.4236, 0.4267) | 0.5734 (0.3231, 0.8244) | 0.1529 (0.1527, 0.1531) | 0.0543 (-0.1135, 0.2223) | 0.3602 (0.3591, 0.3613) |
| Kuwait | -1.1606 (-1.4861, -0.8340) | -0.9230 (-0.9301, -0.9158) | -0.7198 (-1.0541, -0.3844) | -2.2832 (-2.3270, -2.2394) | -1.1206 (-1.4112, -0.8291) | -0.4627 (-0.4645, -0.4609) |
| Kyrgyzstan | 0.1380 (-0.1641, 0.4409) | -5.4476 (-5.6974, -5.1972) | 0.0617 (-0.3002, 0.4249) | -7.5070 (-7.9825, -7.0291) | 0.2355 (-0.1031, 0.5753) | -6.5717 (-6.9356, -6.2063) |
| Lao People's Democratic Republic | -3.1609 (-3.3243, -2.9972) | -0.3341 (-0.3350, -0.3331) | -3.0897 (-3.2463, -2.9329) | -0.9115 (-0.9184, -0.9045) | -3.3765 (-3.5697, -3.1828) | -0.1041 (-0.1042, -0.1040) |
| Latvia | -1.0303 (-1.2617, -0.7984) | -4.1125 (-4.2546, -3.9701) | -0.9492 (-1.2612, -0.6362) | -2.9233 (-2.9950, -2.8514) | -1.0008 (-1.2491, -0.7518) | -4.7235 (-4.9111, -4.5355) |
| Lebanon | -1.6501 (-2.2111, -1.0859) | -6.7282 (-7.1097, -6.3451) | -1.0738 (-1.5997, -0.5450) | -7.1781 (-7.6126, -6.7415) | -1.7752 (-2.3160, -1.2313) | -6.9709 (-7.3806, -6.5594) |
| Lesotho | 3.0407 (2.6647, 3.4181) | 3.5503 (3.4444, 3.6563) | 3.1114 (2.7072, 3.5173) | 4.0070 (3.8721, 4.1421) | 2.7941 (2.3982, 3.1916) | 3.3252 (3.2324, 3.4182) |
| Liberia | -1.9458 (-2.1398, -1.7515) | 0.1445 (0.1444, 0.1447) | -2.0028 (-2.1604, -1.8450) | -0.0755 (-0.0756, -0.0755) | -2.0874 (-2.2808, -1.8937) | 0.2701 (0.2695, 0.2707) |
| Libya | 0.3810 (0.2122, 0.5501) | -1.2572 (-1.2705, -1.2439) | 0.3052 (0.1358, 0.4748) | -1.0912 (-1.1012, -1.0812) | 0.6227 (0.4363, 0.8095) | -1.3769 (-1.3928, -1.3610) |
| Lithuania | 0.3162 (-0.1558, 0.7904) | -15.0706 (-17.0227, -13.0726) | 0.6209 (0.0203, 1.2251) | -15.0542 (-17.0019, -13.0607) | 0.3093 (-0.1805, 0.8015) | -17.0871 (-19.6158, -14.4789) |
| Luxembourg | -2.7013 (-3.1093, -2.2917) | -5.0694 (-5.2856, -4.8527) | -2.3778 (-2.8456, -1.9077) | -4.9421 (-5.1476, -4.7362) | -3.1388 (-3.5672, -2.7085) | -5.4296 (-5.6777, -5.1809) |
| Madagascar | 0.7509 (0.6599, 0.8420) | 1.4935 (1.4748, 1.5123) | 0.7329 (0.6265, 0.8394) | 1.0661 (1.0565, 1.0756) | 0.5907 (0.4955, 0.6859) | 1.3743 (1.3584, 1.3901) |
| Malawi | -1.1013 (-1.2539, -0.9485) | 0.9092 (0.9023, 0.9162) | -0.9857 (-1.1795, -0.7915) | 0.6735 (0.6697, 0.6773) | -1.3973 (-1.5440, -1.2504) | 0.6665 (0.6628, 0.6702) |
| Malaysia | 2.2843 (1.9125, 2.6574) | -6.7256 (-7.1069, -6.3428) | 2.3816 (2.0132, 2.7514) | -4.4208 (-4.5851, -4.2562) | 2.2432 (1.8468, 2.6413) | -8.4925 (-9.1020, -7.8790) |
| Maldives | -3.4637 (-3.7513, -3.1751) | -10.7005 (-11.6724, -9.7180) | -3.1492 (-3.4598, -2.8376) | -14.8332 (-16.7228, -12.9008) | -3.4998 (-3.7724, -3.2264) | -9.6602 (-10.4505, -8.8629) |
| Mali | 0.2261 (-0.0351, 0.4880) | 1.4897 (1.4710, 1.5083) | 0.4526 (0.1092, 0.7971) | 2.5213 (2.4680, 2.5747) | 0.0564 (-0.1867, 0.3000) | 1.2532 (1.2400, 1.2664) |
| Malta | -2.1195 (-2.4595, -1.7782) | 0.6240 (0.6207, 0.6272) | -1.9181 (-2.3171, -1.5174) | 0.6619 (0.6582, 0.6656) | -2.2738 (-2.6203, -1.9262) | 0.7368 (0.7323, 0.7414) |
| Marshall Islands | -0.6377 (-1.2542, -0.0174) | 3.0542 (2.9759, 3.1326) | -1.3133 (-1.8996, -0.7235) | 3.0233 (2.9465, 3.1001) | -0.4579 (-1.0739, 0.1619) | 3.2030 (3.1178, 3.2903) |
| Mauritania | -2.1352 (-2.4116, -1.8581) | 2.4519 (2.4014, 2.5024) | -2.2620 (-2.5535, -1.9697) | 2.9413 (2.8686, 3.0140) | -2.3114 (-2.5751, -2.0469) | 2.0537 (2.0183, 2.0892) |
| Mauritius | -2.0865 (-2.3317, -1.8406) | -0.1108 (-0.1109, -0.1107) | -2.1312 (-2.3890, -1.8729) | -0.9791 (-0.9871, -0.9710) | -2.0061 (-2.2158, -1.7960) | 0.5194 (0.5171, 0.5216) |
| Mexico | -2.7476 (-3.0737, -2.4205) | 2.9634 (2.8897, 3.0372) | -3.0344 (-3.3752, -2.6925) | 3.2202 (3.1331, 3.3074) | -2.8595 (-3.2165, -2.5012) | 3.2003 (3.1143, 3.2864) |
| Micronesia (Federated States of) | -1.3420 (-1.4847, -1.1991) | 0.1904 (0.1901, 0.1907) | -1.5577 (-1.6461, -1.4692) | -0.9965 (-1.0049, -0.9882) | -1.2388 (-1.3604, -1.1170) | 0.5778 (0.5750, 0.5806) |
| Monaco | -1.3215 (-1.5662, -1.0762) | -0.0372 (-0.0372, -0.0372) | -0.9133 (-1.1809, -0.6450) | -1.1525 (-1.1636, -1.1413) | -1.6828 (-1.9326, -1.4325) | 0.6961 (0.6920, 0.7002) |
| Mongolia | 2.7904 (2.3242, 3.2588) | -1.0507 (-1.0600, -1.0414) | 3.0976 (2.5182, 3.6802) | 0.2573 (0.2568, 0.2579) | 2.8786 (2.4161, 3.3431) | -1.9906 (-2.0239, -1.9574) |
| Montenegro | 1.9496 (1.4382, 2.4636) | 5.1585 (4.9347, 5.3828) | 2.0466 (1.3484, 2.7497) | 5.6008 (5.3368, 5.8654) | 2.0706 (1.6195, 2.5236) | 4.9177 (4.7143, 5.1214) |
| Morocco | 0.4805 (0.2219, 0.7397) | 1.4148 (1.3980, 1.4316) | 0.6467 (0.3281, 0.9662) | 1.9109 (1.8803, 1.9416) | 0.3391 (0.1078, 0.5709) | 1.1739 (1.1623, 1.1855) |
| Mozambique | -0.7168 (-1.0637, -0.3686) | 2.8506 (2.7824, 2.9189) | -0.8281 (-1.1548, -0.5003) | 2.8343 (2.7669, 2.9018) | -1.0523 (-1.3877, -0.7157) | 2.7779 (2.7131, 2.8428) |
| Myanmar | -2.0719 (-2.3732, -1.7696) | -2.6073 (-2.6644, -2.5502) | -1.6326 (-1.9978, -1.2660) | -4.3549 (-4.5144, -4.1952) | -2.4589 (-2.7400, -2.1770) | -2.8034 (-2.8694, -2.7373) |
| Namibia | -0.9833 (-1.5557, -0.4077) | 0.2438 (0.2433, 0.2443) | -0.4985 (-1.2090, 0.2172) | 0.0875 (0.0874, 0.0876) | -1.3557 (-1.9107, -0.7975) | -0.0328 (-0.0328, -0.0328) |
| Nauru | -2.4642 (-2.6278, -2.3004) | 0.3747 (0.3736, 0.3759) | -2.6060 (-2.7403, -2.4715) | -0.3746 (-0.3758, -0.3734) | -2.3915 (-2.5509, -2.2318) | 0.7070 (0.7028, 0.7112) |
| Nepal | -0.6059 (-0.7538, -0.4578) | 1.8705 (1.8411, 1.8999) | -0.1494 (-0.2832, -0.0154) | 2.0990 (2.0620, 2.1360) | -1.3208 (-1.4813, -1.1601) | 1.5376 (1.5178, 1.5575) |
| Netherlands | -0.9590 (-1.0834, -0.8344) | 0.0579 (0.0579, 0.0579) | -0.9164 (-1.0457, -0.7869) | -0.2796 (-0.2803, -0.2790) | -1.1051 (-1.2141, -0.9960) | 0.1903 (0.1900, 0.1906) |
| New Zealand | -1.6999 (-1.7599, -1.6399) | -1.4996 (-1.5185, -1.4807) | -1.5459 (-1.6000, -1.4918) | -1.9634 (-1.9957, -1.9310) | -1.8367 (-1.9071, -1.7662) | -0.7857 (-0.7909, -0.7805) |
| Nicaragua | 0.8056 (0.4657, 1.1466) | 1.7201 (1.6953, 1.7449) | 1.1519 (0.7987, 1.5063) | 1.9250 (1.8939, 1.9561) | 0.7851 (0.4413, 1.1301) | 1.8244 (1.7965, 1.8524) |
| Niger | -1.0581 (-1.1845, -0.9316) | 0.5360 (0.5336, 0.5384) | -1.0660 (-1.1914, -0.9404) | -0.1300 (-0.1302, -0.1299) | -1.2939 (-1.4114, -1.1762) | 0.4039 (0.4025, 0.4053) |
| Nigeria | -0.7447 (-0.8645, -0.6248) | 0.4224 (0.4209, 0.4239) | -0.6515 (-0.7217, -0.5811) | 0.2000 (0.1996, 0.2003) | -1.2430 (-1.3729, -1.1129) | 0.3865 (0.3852, 0.3877) |
| Niue | 1.0490 (0.8244, 1.2741) | -1.6903 (-1.7143, -1.6663) | 0.9336 (0.6664, 1.2016) | -1.7343 (-1.7596, -1.7091) | 1.3734 (1.1295, 1.6178) | -1.5377 (-1.5576, -1.5179) |
| North Macedonia | 13.0461 (11.8895, 14.2147) | 3.4056 (3.3081, 3.5031) | 14.6168 (13.4190, 15.8273) | 3.9028 (3.7748, 4.0310) | 12.7894 (11.5760, 14.0160) | 3.0579 (2.9794, 3.1365) |
| Northern Mariana Islands | -2.3989 (-2.7929, -2.0033) | 1.9374 (1.9059, 1.9689) | -2.8025 (-3.1642, -2.4394) | 1.9429 (1.9112, 1.9746) | -2.3506 (-2.7150, -1.9849) | 1.9485 (1.9166, 1.9804) |
| Norway | -1.3434 (-1.4193, -1.2675) | 0.3067 (0.3059, 0.3075) | -0.8289 (-0.8915, -0.7662) | -0.9321 (-0.9394, -0.9248) | -1.9171 (-2.0978, -1.7360) | 1.3569 (1.3414, 1.3723) |
| Oman | 0.0454 (-0.7896, 0.8875) | 4.0973 (3.9562, 4.2385) | -0.2820 (-1.1526, 0.5963) | 4.4384 (4.2728, 4.6042) | 0.2531 (-0.5739, 1.0870) | 4.0385 (3.9014, 4.1757) |
| Pakistan | -0.4799 (-0.7061, -0.2531) | 1.3307 (1.3159, 1.3456) | -0.5718 (-0.7787, -0.3645) | 1.3611 (1.3456, 1.3767) | -0.8531 (-1.1103, -0.5952) | 1.1116 (1.1013, 1.1220) |
| Palau | -1.2037 (-1.4950, -0.9116) | 0.3498 (0.3487, 0.3508) | -1.2151 (-1.4466, -0.9831) | -1.5599 (-1.5804, -1.5395) | -1.0381 (-1.3281, -0.7473) | 1.0411 (1.0320, 1.0502) |
| Palestine | 0.6668 (-0.0397, 1.3783) | -12.1238 (-13.3758, -10.8538) | 0.8493 (0.1058, 1.5984) | -19.1770 (-22.3916, -15.8294) | 0.6950 (0.0156, 1.3789) | -9.7044 (-10.5020, -8.8996) |
| Panama | -0.8539 (-0.9495, -0.7582) | 0.1084 (0.1083, 0.1085) | -0.4910 (-0.5430, -0.4389) | 0.1003 (0.1002, 0.1004) | -1.0352 (-1.1481, -0.9222) | 0.1531 (0.1529, 0.1533) |
| Papua New Guinea | -3.2954 (-3.6041, -2.9857) | 1.1958 (1.1838, 1.2078) | -3.2712 (-3.6208, -2.9204) | 0.0922 (0.0921, 0.0923) | -3.3782 (-3.6910, -3.0644) | 1.2453 (1.2323, 1.2584) |
| Paraguay | -0.4553 (-0.6400, -0.2703) | 1.5918 (1.5705, 1.6131) | -0.2330 (-0.3621, -0.1036) | 1.1134 (1.1030, 1.1238) | -0.6833 (-0.8872, -0.4789) | 1.7118 (1.6872, 1.7364) |
| Peru | -0.9215 (-1.1134, -0.7292) | 0.8148 (0.8093, 0.8204) | -0.6408 (-0.7693, -0.5121) | 0.6546 (0.6510, 0.6582) | -0.9403 (-1.1835, -0.6964) | 0.5974 (0.5944, 0.6004) |
| Philippines | -5.0129 (-6.5190, -3.4826) | 2.3405 (2.2946, 2.3866) | -5.6853 (-7.3855, -3.9539) | -0.0203 (-0.0203, -0.0203) | -5.2041 (-6.6625, -3.7230) | 3.3951 (3.2983, 3.4921) |
| Poland | -1.7718 (-2.2202, -1.3214) | -13.7207 (-15.3316, -12.0792) | -1.4967 (-2.0097, -0.9810) | -20.4945 (-24.1896, -16.6192) | -1.9634 (-2.4293, -1.4953) | -14.4987 (-16.3019, -12.6566) |
| Portugal | -3.7606 (-3.9282, -3.5927) | -1.5410 (-1.5609, -1.5211) | -3.5756 (-3.7697, -3.3811) | -0.7600 (-0.7648, -0.7551) | -3.8942 (-4.0430, -3.7451) | -2.1429 (-2.1814, -2.1043) |
| Puerto Rico | -3.0349 (-3.5252, -2.5421) | -2.3489 (-2.3952, -2.3026) | -2.6684 (-3.2365, -2.0971) | -1.9952 (-2.0286, -1.9618) | -3.1224 (-3.6044, -2.6380) | -1.9717 (-2.0043, -1.9391) |
| Qatar | 0.2724 (-0.6962, 1.2504) | -85.3979 (-93.1383, -68.9255) | -0.0530 (-1.1125, 1.0178) | -76.5709 (-85.5561, -61.9964) | 0.6548 (-0.2571, 1.5749) | -82.5664 (-91.4038, -64.6436) |
| Republic of Korea | -4.2404 (-4.3962, -4.0844) | -9.1446 (-9.8521, -8.4316) | -4.5959 (-4.7909, -4.4005) | -8.8323 (-9.4920, -8.1679) | -3.8171 (-3.9739, -3.6600) | -10.4857 (-11.4185, -9.5431) |
| Republic of Moldova | -4.2184 (-4.4586, -3.9775) | -15.2461 (-17.2452, -13.1988) | -4.3656 (-4.6477, -4.0828) | -16.8729 (-19.3365, -14.3342) | -4.1698 (-4.3979, -3.9412) | -17.7240 (-20.4519, -14.9025) |
| Romania | -3.6258 (-4.0663, -3.1834) | -84.4990 (-92.0805, -69.6595) | -3.6272 (-4.1673, -3.0841) | -73.6195 (-82.0800, -61.1646) | -3.6732 (-4.1044, -3.2400) | -81.0489 (-89.3452, -66.2927) |
| Russian Federation | -1.9819 (-2.4835, -1.4778) | -17.5781 (-20.2597, -14.8064) | -1.8538 (-2.2960, -1.4096) | -18.6861 (-21.7312, -15.5225) | -2.0668 (-2.6352, -1.4950) | -20.8225 (-24.6432, -16.8080) |
| Rwanda | -1.1401 (-1.3578, -0.9219) | -0.0009 (-0.0009, -0.0009) | -0.9033 (-1.1602, -0.6457) | -0.0322 (-0.0322, -0.0322) | -1.4897 (-1.7000, -1.2789) | -0.2478 (-0.2483, -0.2472) |
| Saint Kitts and Nevis | -3.3419 (-3.7768, -2.9050) | 1.7667 (1.7405, 1.7929) | -3.6779 (-4.0245, -3.3299) | -0.4254 (-0.4269, -0.4239) | -3.5562 (-4.0251, -3.0851) | 2.0833 (2.0469, 2.1198) |
| Saint Lucia | -4.6740 (-6.0087, -3.3203) | 2.8976 (2.8271, 2.9681) | -4.6756 (-6.2173, -3.1086) | 3.9841 (3.8507, 4.1177) | -4.7769 (-6.0280, -3.5091) | 2.2468 (2.2044, 2.2892) |
| Saint Vincent and the Grenadines | -1.0701 (-1.4717, -0.6670) | 0.9023 (0.8955, 0.9091) | -1.0659 (-1.4991, -0.6308) | 1.4002 (1.3837, 1.4166) | -1.0643 (-1.4470, -0.6800) | 0.4647 (0.4629, 0.4665) |
| Samoa | -0.4780 (-0.9192, -0.0349) | -2.0108 (-2.0447, -1.9768) | -0.1231 (-0.6088, 0.3649) | -3.4220 (-3.5204, -3.3235) | -0.4960 (-0.9885, -0.0012) | -1.4327 (-1.4499, -1.4154) |
| San Marino | -1.2289 (-1.4657, -0.9916) | -1.1140 (-1.1244, -1.1036) | -0.2000 (-0.3819, -0.0177) | -1.6363 (-1.6588, -1.6138) | -1.8789 (-2.1150, -1.6423) | -0.9247 (-0.9319, -0.9175) |
| Sao Tome and Principe | 0.8762 (0.6921, 1.0606) | 1.0538 (1.0445, 1.0631) | 1.1102 (0.8738, 1.3471) | 1.2956 (1.2816, 1.3097) | 0.7412 (0.5588, 0.9241) | 0.6645 (0.6607, 0.6682) |
| Saudi Arabia | -0.0092 (-0.3504, 0.3331) | 0.8719 (0.8655, 0.8782) | -0.2489 (-0.6393, 0.1429) | -1.4694 (-1.4875, -1.4512) | 0.1704 (-0.1693, 0.5113) | 1.5663 (1.5457, 1.5869) |
| Senegal | -0.7100 (-0.8615, -0.5582) | 0.3163 (0.3155, 0.3172) | -0.5255 (-0.7079, -0.3428) | 0.0926 (0.0925, 0.0927) | -1.1351 (-1.2989, -0.9710) | -0.0438 (-0.0438, -0.0437) |
| Serbia | -3.6843 (-5.3622, -1.9766) | 9.6416 (8.8556, 10.4333) | -3.4695 (-5.1394, -1.7702) | 9.4331 (8.6809, 10.1904) | -3.6747 (-5.4057, -1.9120) | 10.0610 (9.2046, 10.9241) |
| Seychelles | 0.2975 (0.2726, 0.3224) | 0.0808 (0.0807, 0.0809) | 0.2898 (0.2230, 0.3567) | -0.6674 (-0.6711, -0.6636) | 0.4301 (0.4002, 0.4600) | 0.3046 (0.3038, 0.3054) |
| Sierra Leone | -0.2229 (-0.4632, 0.0179) | 1.1859 (1.1741, 1.1977) | -0.2502 (-0.4730, -0.0269) | 1.5557 (1.5354, 1.5760) | -0.4117 (-0.6374, -0.1855) | 0.8998 (0.8930, 0.9066) |
| Singapore | -1.4660 (-1.6381, -1.2936) | -2.6700 (-2.7299, -2.6101) | -1.3640 (-1.5767, -1.1509) | -2.4355 (-2.4853, -2.3856) | -1.4107 (-1.5783, -1.2428) | -1.5383 (-1.5581, -1.5184) |
| Slovakia | -1.1244 (-1.4057, -0.8424) | 0.4950 (0.4929, 0.4970) | -1.1538 (-1.5016, -0.8047) | -0.1792 (-0.1795, -0.1790) | -1.1482 (-1.4038, -0.8919) | 0.6215 (0.6182, 0.6247) |
| Slovenia | -0.9779 (-1.3376, -0.6169) | -8.0658 (-8.6152, -7.5131) | -0.7156 (-1.1102, -0.3195) | -7.7304 (-8.2348, -7.2232) | -0.9973 (-1.3873, -0.6058) | -8.6941 (-9.3331, -8.0506) |
| Solomon Islands | -1.3971 (-1.6239, -1.1697) | -4.4878 (-4.6571, -4.3182) | -1.1271 (-1.3785, -0.8750) | -5.3899 (-5.6344, -5.1448) | -1.3910 (-1.6424, -1.1388) | -3.9724 (-4.1050, -3.8396) |
| Somalia | 3.7392 (3.3723, 4.1074) | -1.9863 (-2.0194, -1.9532) | 4.0494 (3.6208, 4.4798) | -1.8220 (-1.8499, -1.7941) | 3.3072 (2.9654, 3.6501) | -1.9756 (-2.0084, -1.9428) |
| South Africa | 1.4258 (0.9937, 1.8597) | -0.5572 (-0.5598, -0.5546) | 1.7935 (1.2959, 2.2935) | -1.4468 (-1.4644, -1.4292) | 1.1849 (0.7946, 1.5767) | -0.3974 (-0.3987, -0.3960) |
| South Sudan | -2.0244 (-2.4796, -1.5672) | 3.4138 (3.3159, 3.5118) | -2.2502 (-2.6357, -1.8632) | 3.4112 (3.3134, 3.5090) | -2.1763 (-2.6261, -1.7244) | 3.2366 (3.1486, 3.3247) |
| Spain | -2.2077 (-2.3748, -2.0402) | -5.1553 (-5.3789, -4.9312) | -1.7207 (-1.9247, -1.5162) | -4.7935 (-4.9867, -4.5998) | -2.5639 (-2.7463, -2.3811) | -4.7036 (-4.8896, -4.5172) |
| Sri Lanka | -1.9629 (-2.4778, -1.4452) | 0.4065 (0.4051, 0.4079) | -2.2943 (-2.8443, -1.7413) | 0.8919 (0.8852, 0.8985) | -2.0153 (-2.5435, -1.4842) | 0.4665 (0.4646, 0.4683) |
| Sudan | -3.2455 (-3.6709, -2.8182) | 2.1391 (2.1007, 2.1776) | -3.4426 (-3.8397, -3.0439) | 2.2898 (2.2458, 2.3339) | -3.1912 (-3.6301, -2.7503) | 2.1439 (2.1053, 2.1825) |
| Suriname | 0.2840 (0.0222, 0.5466) | -0.2975 (-0.2983, -0.2968) | 0.3182 (0.1240, 0.5127) | -0.0648 (-0.0648, -0.0648) | 0.3533 (0.0497, 0.6579) | -0.5521 (-0.5546, -0.5495) |
| Sweden | -1.5231 (-1.7064, -1.3394) | 0.6181 (0.6149, 0.6213) | -1.2857 (-1.3758, -1.1955) | -1.1258 (-1.1365, -1.1152) | -1.9044 (-2.1971, -1.6109) | 2.0755 (2.0393, 2.1117) |
| Switzerland | -1.4739 (-1.7008, -1.2465) | -0.2199 (-0.2203, -0.2195) | -1.0946 (-1.3148, -0.8739) | -0.5364 (-0.5388, -0.5340) | -1.7432 (-1.9978, -1.4879) | 0.1620 (0.1618, 0.1622) |
| Syrian Arab Republic | 3.7363 (2.9828, 4.4954) | -7.3992 (-7.8611, -6.9351) | 4.5281 (3.7702, 5.2916) | -8.4358 (-9.0371, -7.8305) | 3.4772 (2.7011, 4.2592) | -7.0845 (-7.5077, -6.6594) |
| Taiwan (Province of China) | -3.4741 (-3.6822, -3.2655) | 1.1002 (1.0901, 1.1104) | -3.2145 (-3.4869, -2.9412) | 2.4020 (2.3535, 2.4504) | -3.3526 (-3.4770, -3.2280) | -1.7916 (-1.8185, -1.7646) |
| Tajikistan | 0.9761 (0.7130, 1.2399) | -3.2930 (-3.3841, -3.2018) | 0.9675 (0.6636, 1.2723) | -3.9790 (-4.1121, -3.8458) | 0.8621 (0.5651, 1.1600) | -4.4661 (-4.6338, -4.2981) |
| Thailand | -1.4539 (-1.6363, -1.2712) | -5.3006 (-5.5370, -5.0636) | -1.3253 (-1.4425, -1.2080) | -4.3790 (-4.5402, -4.2175) | -1.2766 (-1.5398, -1.0127) | -5.9810 (-6.2822, -5.6788) |
| Timor-Leste | 1.7198 (1.1282, 2.3148) | -2.1164 (-2.1540, -2.0788) | 2.5626 (1.9762, 3.1524) | -3.0515 (-3.1298, -2.9733) | 1.2698 (0.6701, 1.8730) | -1.3025 (-1.3167, -1.2882) |
| Togo | 0.6321 (0.4803, 0.7841) | 0.5914 (0.5885, 0.5944) | 0.5416 (0.3568, 0.7267) | 0.8204 (0.8147, 0.8260) | 0.4138 (0.2818, 0.5460) | 0.3412 (0.3402, 0.3422) |
| Tokelau | -1.8679 (-2.1635, -1.5713) | -10.3543 (-11.2637, -9.4357) | -1.7131 (-2.0535, -1.3716) | -15.3533 (-17.3814, -13.2755) | -1.6584 (-1.9672, -1.3486) | -10.0958 (-10.9598, -9.2235) |
| Tonga | -1.3470 (-1.4714, -1.2225) | -0.1389 (-0.1390, -0.1387) | -1.0683 (-1.2241, -0.9123) | 0.2601 (0.2595, 0.2607) | -1.4029 (-1.5072, -1.2985) | -0.1179 (-0.1180, -0.1178) |
| Trinidad and Tobago | -4.5066 (-5.5478, -3.4540) | 1.5923 (1.5711, 1.6136) | -5.1064 (-6.2155, -3.9841) | 0.9745 (0.9665, 0.9824) | -4.4149 (-5.4372, -3.3816) | 2.0949 (2.0581, 2.1318) |
| Tunisia | -4.0509 (-4.5114, -3.5881) | -0.7966 (-0.8020, -0.7913) | -4.2905 (-4.7705, -3.8081) | -1.6310 (-1.6534, -1.6087) | -3.9139 (-4.3750, -3.4507) | -0.3885 (-0.3897, -0.3872) |
| Turkmenistan | -1.5943 (-1.8771, -1.3106) | -1.8603 (-1.8893, -1.8312) | -1.5908 (-1.7945, -1.3867) | -1.7395 (-1.7649, -1.7140) | -1.6050 (-1.9384, -1.2705) | -3.1834 (-3.2685, -3.0982) |
| Tuvalu | -2.9167 (-3.3363, -2.4953) | -2.6637 (-2.7233, -2.6041) | -2.6292 (-3.1867, -2.0685) | -5.4735 (-5.7257, -5.2207) | -2.9196 (-3.3290, -2.5085) | -1.7716 (-1.7980, -1.7453) |
| Uganda | -0.7039 (-0.9058, -0.5016) | -0.3261 (-0.3270, -0.3252) | -0.7126 (-0.9484, -0.4763) | 0.1510 (0.1508, 0.1512) | -1.1354 (-1.3283, -0.9422) | -0.5644 (-0.5671, -0.5618) |
| Ukraine | -1.1482 (-1.8856, -0.4052) | -12.1964 (-13.4637, -10.9106) | -0.9737 (-1.6398, -0.3031) | -34.4860 (-45.5510, -21.1725) | -1.2794 (-2.0888, -0.4634) | -10.7782 (-11.7644, -9.7810) |
| United Arab Emirates | 2.9774 (1.9946, 3.9697) | 3.6218 (3.5116, 3.7321) | 1.7332 (0.7696, 2.7060) | 2.5468 (2.4923, 2.6013) | 3.5583 (2.5493, 4.5773) | 3.9547 (3.8233, 4.0863) |
| United Kingdom | -1.7503 (-1.9786, -1.5214) | 0.7884 (0.7831, 0.7936) | -1.5517 (-1.7632, -1.3397) | 0.2141 (0.2137, 0.2145) | -1.9924 (-2.2244, -1.7598) | 1.4318 (1.4146, 1.4490) |
| United Republic of Tanzania | -2.3556 (-2.5814, -2.1293) | -0.5373 (-0.5397, -0.5349) | -2.4097 (-2.6598, -2.1589) | 0.1133 (0.1132, 0.1134) | -2.5216 (-2.7443, -2.2984) | -1.0498 (-1.0591, -1.0406) |
| United States of America | -0.8801 (-0.9372, -0.8229) | 0.3733 (0.3722, 0.3745) | -0.6958 (-0.7487, -0.6430) | -0.5188 (-0.5211, -0.5166) | -1.0844 (-1.1675, -1.0013) | 0.7486 (0.7439, 0.7533) |
| United States Virgin Islands | -2.7874 (-3.3936, -2.1774) | 0.3664 (0.3653, 0.3676) | -3.2309 (-3.8837, -2.5736) | 0.0896 (0.0895, 0.0896) | -2.4383 (-3.0634, -1.8092) | 0.9408 (0.9334, 0.9483) |
| Uruguay | -1.3859 (-1.5724, -1.1991) | -1.5195 (-1.5389, -1.5001) | -0.9283 (-1.1435, -0.7127) | -1.3439 (-1.3590, -1.3287) | -1.7555 (-1.9201, -1.5907) | -1.6467 (-1.6695, -1.6240) |
| Uzbekistan | 1.0227 (0.5876, 1.4596) | -3.6834 (-3.7974, -3.5693) | 0.6830 (0.1425, 1.2264) | -4.3461 (-4.5049, -4.1870) | 1.2014 (0.7422, 1.6627) | -4.5390 (-4.7122, -4.3654) |
| Vanuatu | -1.2747 (-1.4674, -1.0816) | -1.2680 (-1.2815, -1.2545) | -1.1819 (-1.4444, -0.9187) | -1.5203 (-1.5397, -1.5009) | -1.3401 (-1.5397, -1.1402) | -0.9532 (-0.9608, -0.9456) |
| Venezuela (Bolivarian Republic of) | -1.0641 (-1.4538, -0.6728) | 1.7228 (1.6979, 1.7477) | -1.0369 (-1.4432, -0.6288) | 1.1617 (1.1503, 1.1730) | -1.1367 (-1.5042, -0.7677) | 1.5280 (1.5084, 1.5476) |
| Viet Nam | 0.1503 (-0.0892, 0.3903) | -1.5089 (-1.5280, -1.4898) | 0.6000 (0.3385, 0.8622) | -2.4530 (-2.5036, -2.4025) | -0.1944 (-0.4298, 0.0415) | -1.6874 (-1.7113, -1.6635) |
| Yemen | -1.6763 (-1.8068, -1.5456) | 0.7671 (0.7622, 0.7720) | -1.5829 (-1.6972, -1.4684) | 0.6499 (0.6464, 0.6535) | -1.7176 (-1.8577, -1.5773) | 0.7504 (0.7457, 0.7552) |
| Zambia | 0.0481 (-0.1863, 0.2830) | 0.2520 (0.2515, 0.2525) | 0.1197 (-0.1898, 0.4302) | 0.0524 (0.0524, 0.0525) | -0.1771 (-0.4010, 0.0473) | 0.2806 (0.2799, 0.2812) |
| Zimbabwe | 1.0385 (0.8267, 1.2508) | 3.5224 (3.4182, 3.6267) | 1.0841 (0.8713, 1.2973) | 3.4970 (3.3943, 3.5999) | 0.9850 (0.7600, 1.2105) | 3.2633 (3.1739, 3.3528) |

**STable 7. The projected age-standardized rates of Alzheimer's Disease in 2019 and 2030 in different countries.**

| location | Deaths | | DALYs (Disability-Adjusted Life Years) | | Incidence | |
| --- | --- | --- | --- | --- | --- | --- |
|  | 2019 | 2030 | 2019 | 2030 | 2019 | 2030 |
| Afghanistan | 24313.90 (24031.54, 24599.15) | 23605.94 (21987.23, 25224.65) | 1159.21 (1096.79, 1224.61) | 1161.05 (1077.38, 1244.73) | 6078.23 (5938.74, 6220.57) | 5760.18 (5352.03, 6168.33) |
| Albania | 9480.66 (9322.94, 9640.67) | 9247.26 (8847.97, 9646.55) | 401.19 (369.31, 435.41) | 413.93 (393.23, 434.63) | 2998.59 (2909.83, 3089.66) | 2838.08 (2738.27, 2937.89) |
| Algeria | 42195.47 (41901.30, 42491.42) | 10446.63 (0.05, 21217.58) | 2060.17 (1994.25, 2127.93) | 632.34 (0.05, 1265.18) | 12245.16 (12087.92, 12404.17) | 2841.14 (167.97, 5514.31) |
| American Samoa | 12494.47 (10290.19, 15096.93) | 10455.23 (9789.01, 11121.44) | 544.65 (168.85, 1407.70) | 419.66 (378.91, 460.40) | 3670.22 (2529.30, 5227.70) | 3296.94 (3118.75, 3475.14) |
| Andorra | 4955.30 (4567.45, 5375.20) | 8885.39 (4439.77, 13331.01) | 258.81 (176.54, 377.10) | 414.90 (152.63, 677.17) | 1399.35 (1197.86, 1633.81) | 2768.38 (1580.18, 3956.58) |
| Angola | 25152.39 (24852.64, 25455.31) | 23994.58 (22322.57, 25666.59) | 1087.66 (1024.42, 1154.21) | 1070.86 (993.78, 1147.94) | 6307.60 (6159.10, 6459.26) | 5662.85 (5238.02, 6087.69) |
| Antigua and Barbuda | 7459.65 (6526.12, 8505.21) | 8899.27 (8509.76, 9288.78) | 293.77 (134.42, 582.59) | 333.49 (316.76, 350.22) | 2487.16 (1959.43, 3130.58) | 3052.20 (2931.28, 3173.12) |
| Argentina | 5542.23 (5517.78, 5566.77) | 6787.88 (6366.45, 7209.31) | 249.36 (244.35, 254.46) | 289.95 (265.75, 314.15) | 1739.28 (1725.43, 1753.24) | 2220.63 (2094.68, 2346.58) |
| Armenia | 11056.62 (10863.71, 11252.54) | 0.05 (0.05, 1653.38) | 529.61 (487.21, 575.14) | 0.05 (0.05, 58.38) | 3319.07 (3213.95, 3427.20) | 0.05 (0.05, 591.20) |
| Australia | 5328.49 (5304.16, 5352.92) | 4451.26 (4111.43, 4791.08) | 262.35 (257.04, 267.75) | 194.78 (171.50, 218.06) | 1454.75 (1442.02, 1467.59) | 1324.93 (1254.48, 1395.37) |
| Austria | 5248.37 (5214.10, 5282.85) | 3503.89 (2416.32, 4591.45) | 262.64 (255.18, 270.31) | 169.98 (102.70, 237.27) | 1545.29 (1526.58, 1564.21) | 1024.76 (742.04, 1307.48) |
| Azerbaijan | 8842.10 (8730.44, 8955.03) | 10780.76 (10470.10, 11091.42) | 364.06 (341.88, 387.52) | 485.33 (468.92, 501.75) | 2761.44 (2698.99, 2825.16) | 3091.78 (2957.36, 3226.21) |
| Bahamas | 5804.13 (5434.34, 6196.42) | 6370.58 (6116.78, 6624.38) | 219.89 (155.21, 308.49) | 236.65 (226.87, 246.43) | 1917.91 (1705.92, 2153.01) | 2131.38 (2034.47, 2228.30) |
| Bahrain | 72359.86 (67217.67, 77837.08) | 0.05 (0.05, 12522.80) | 3523.92 (2427.52, 4994.87) | 0.05 (0.05, 863.09) | 21440.94 (18745.66, 24469.40) | 0.05 (0.05, 3395.84) |
| Bangladesh | 10983.91 (10941.05, 11026.93) | 4882.55 (3589.23, 6175.87) | 508.92 (499.46, 518.52) | 292.63 (236.32, 348.95) | 3001.41 (2979.04, 3023.93) | 1098.69 (786.37, 1411.01) |
| Barbados | 5936.66 (5632.25, 6255.27) | 4299.04 (3918.71, 4679.37) | 249.69 (190.49, 324.00) | 160.46 (141.81, 179.11) | 1896.86 (1726.25, 2081.86) | 1380.60 (1272.92, 1488.28) |
| Belarus | 6833.68 (6779.14, 6888.61) | 2845.30 (1881.13, 3809.47) | 306.83 (295.34, 318.70) | 108.12 (65.32, 150.92) | 2126.46 (2096.05, 2157.25) | 740.85 (421.06, 1060.65) |
| Belgium | 5681.54 (5649.95, 5713.29) | 3501.80 (2707.42, 4296.18) | 304.87 (297.50, 312.40) | 181.93 (136.58, 227.28) | 1571.80 (1555.34, 1588.42) | 1030.59 (823.08, 1238.09) |
| Belize | 5460.25 (5061.29, 5886.88) | 5257.73 (5019.79, 5495.67) | 215.86 (144.64, 316.86) | 187.15 (176.62, 197.68) | 1745.24 (1521.49, 1997.40) | 1708.59 (1638.32, 1778.87) |
| Benin | 8103.27 (7939.39, 8270.11) | 7631.63 (7239.28, 8023.97) | 348.09 (314.95, 384.25) | 319.25 (301.71, 336.78) | 2126.51 (2042.92, 2213.10) | 2147.00 (2027.53, 2266.47) |
| Bermuda | 4577.02 (4165.79, 5025.12) | 3886.01 (3688.33, 4083.70) | 195.87 (120.50, 312.24) | 175.01 (163.69, 186.33) | 1478.58 (1247.02, 1748.31) | 1237.43 (1186.15, 1288.71) |
| Bhutan | 7704.31 (7317.54, 8108.83) | 6287.30 (5995.70, 6578.90) | 365.36 (285.30, 464.18) | 295.39 (280.44, 310.34) | 1886.31 (1697.02, 2093.68) | 1533.99 (1456.37, 1611.62) |
| Bolivia (Plurinational State of) | 7792.78 (7680.15, 7906.82) | 7797.14 (7044.13, 8550.15) | 325.90 (303.07, 350.16) | 294.42 (261.74, 327.10) | 2336.57 (2274.73, 2399.83) | 2401.14 (2177.31, 2624.98) |
| Bosnia and Herzegovina | 8052.43 (7944.81, 8161.34) | 5519.38 (4298.96, 6739.80) | 352.33 (330.30, 375.67) | 268.34 (202.48, 334.20) | 2567.30 (2506.28, 2629.64) | 1559.96 (1192.06, 1927.86) |
| Botswana | 12712.11 (12269.65, 13168.10) | 10177.65 (9102.79, 11252.51) | 538.62 (449.91, 641.53) | 413.96 (362.49, 465.43) | 3526.50 (3295.67, 3770.97) | 2991.43 (2739.07, 3243.78) |
| Brazil | 8472.02 (8455.92, 8488.14) | 7465.70 (6969.03, 7962.37) | 363.96 (360.74, 367.20) | 309.90 (278.25, 341.55) | 2281.19 (2272.77, 2289.64) | 2104.60 (2017.94, 2191.25) |
| Brunei Darussalam | 48287.56 (43190.40, 53893.48) | 50716.72 (43843.82, 57589.62) | 2495.11 (1419.88, 4152.50) | 2529.95 (2117.29, 2942.60) | 13475.72 (10894.76, 16571.34) | 14678.66 (14056.16, 15301.16) |
| Bulgaria | 16790.56 (16616.14, 16966.58) | 0.05 (0.05, 54018.61) | 817.38 (778.49, 857.94) | 0.05 (0.05, 2871.58) | 5216.77 (5120.07, 5315.07) | 0.05 (0.05, 16859.28) |
| Burkina Faso | 10076.49 (9928.32, 10226.56) | 9908.54 (9192.51, 10624.57) | 441.73 (411.05, 474.35) | 432.70 (396.62, 468.79) | 2588.82 (2513.99, 2665.57) | 2604.89 (2430.68, 2779.11) |
| Burundi | 15001.21 (14702.94, 15304.70) | 14383.05 (13696.82, 15069.28) | 654.13 (591.99, 721.72) | 606.89 (576.94, 636.84) | 3869.62 (3719.56, 4024.91) | 3701.94 (3497.38, 3906.49) |
| Cabo Verde | 4542.14 (4288.80, 4809.33) | 5129.06 (4997.79, 5260.33) | 226.45 (172.68, 295.17) | 261.34 (252.47, 270.21) | 1167.74 (1042.23, 1307.26) | 1212.38 (1171.64, 1253.12) |
| Cambodia | 19531.85 (19307.55, 19758.42) | 20750.75 (20593.04, 20908.45) | 844.34 (797.05, 894.01) | 913.03 (906.48, 919.58) | 5114.85 (5000.85, 5231.12) | 5365.71 (5302.92, 5428.51) |
| Cameroon | 11525.14 (11362.16, 11690.19) | 8625.95 (7899.14, 9352.76) | 518.68 (483.70, 555.81) | 381.57 (346.48, 416.65) | 2927.91 (2846.12, 3011.76) | 2257.66 (2105.24, 2410.09) |
| Canada | 5598.47 (5578.88, 5618.12) | 4875.70 (4529.05, 5222.34) | 238.62 (234.65, 242.66) | 215.59 (205.45, 225.73) | 1893.55 (1882.09, 1905.06) | 1595.03 (1431.76, 1758.29) |
| Central African Republic | 40489.59 (39276.85, 41735.02) | 52156.45 (47941.08, 56371.83) | 1697.56 (1449.03, 1981.18) | 2348.17 (2173.98, 2522.37) | 10699.28 (10089.27, 11341.77) | 13262.81 (12139.08, 14386.54) |
| Chad | 10267.85 (10061.00, 10478.43) | 12015.62 (11414.47, 12616.76) | 442.15 (399.91, 488.23) | 527.17 (501.00, 553.34) | 2717.22 (2611.12, 2827.07) | 3123.85 (2968.44, 3279.26) |
| Chile | 5661.16 (5622.53, 5700.02) | 4409.51 (3609.53, 5209.48) | 249.74 (241.81, 257.91) | 192.17 (146.26, 238.08) | 1836.04 (1813.92, 1858.40) | 1380.62 (1162.44, 1598.80) |
| China | 14865.12 (14854.44, 14875.79) | 10456.64 (6752.15, 14161.13) | 658.72 (656.45, 661.00) | 436.69 (249.23, 624.14) | 4423.05 (4417.25, 4428.85) | 3075.06 (2043.66, 4106.46) |
| Colombia | 4895.48 (4872.59, 4918.46) | 4446.55 (4254.47, 4638.63) | 206.32 (201.77, 210.97) | 199.26 (190.82, 207.71) | 1591.33 (1578.27, 1604.49) | 1365.08 (1308.39, 1421.76) |
| Comoros | 14157.58 (13410.62, 14939.35) | 11996.93 (11429.77, 12564.09) | 646.02 (493.61, 835.44) | 552.32 (521.01, 583.64) | 3343.03 (2986.19, 3735.15) | 2748.53 (2625.09, 2871.98) |
| Congo | 29648.20 (28895.44, 30418.37) | 24223.12 (14295.54, 34150.71) | 1406.71 (1241.02, 1591.01) | 1098.16 (391.62, 1804.71) | 7074.64 (6712.83, 7453.81) | 5899.87 (4050.28, 7749.46) |
| Cook Islands | 8515.79 (6774.48, 10637.18) | 5813.26 (5086.57, 6539.95) | 377.51 (95.13, 1130.96) | 265.57 (232.74, 298.39) | 2579.19 (1664.41, 3897.03) | 1818.46 (1623.97, 2012.94) |
| Costa Rica | 4999.44 (4924.31, 5075.59) | 5595.86 (5404.50, 5787.21) | 216.21 (201.18, 232.26) | 235.43 (225.00, 245.85) | 1597.02 (1554.53, 1640.55) | 1782.05 (1714.87, 1849.23) |
| Côte d'Ivoire | 10287.17 (10131.86, 10444.54) | 8739.98 (8246.67, 9233.28) | 444.45 (412.22, 478.80) | 346.82 (321.71, 371.94) | 2691.66 (2612.56, 2772.83) | 2323.60 (2224.93, 2422.28) |
| Croatia | 9155.97 (9053.68, 9259.28) | 4957.50 (3413.84, 6501.16) | 430.48 (408.25, 453.77) | 208.48 (137.45, 279.51) | 2849.07 (2792.18, 2906.99) | 1567.89 (1079.87, 2055.91) |
| Cuba | 4044.93 (4012.37, 4077.73) | 7316.79 (2822.17, 11811.41) | 174.88 (168.36, 181.65) | 339.64 (99.46, 579.83) | 1271.77 (1253.42, 1290.35) | 2337.98 (970.72, 3705.24) |
| Cyprus | 54418.01 (52745.94, 56134.20) | 296472.95 (127878.51, 465067.40) | 3004.05 (2619.51, 3434.16) | 17357.37 (7484.31, 27230.43) | 15230.03 (14352.40, 16152.06) | 76644.31 (35594.75, 117693.87) |
| Czechia | 5798.29 (5758.64, 5838.18) | 2754.85 (841.80, 4667.90) | 249.19 (241.30, 257.32) | 48.97 (0.05, 149.02) | 1816.42 (1794.05, 1839.04) | 988.37 (390.90, 1585.83) |
| Democratic People's Republic of Korea | 15981.96 (15876.54, 16087.99) | 21333.09 (10949.54, 31716.65) | 751.00 (727.78, 774.85) | 1110.25 (594.75, 1625.75) | 4386.37 (4331.55, 4441.79) | 5792.88 (2866.29, 8719.48) |
| Democratic Republic of the Congo | 21693.44 (21558.18, 21829.43) | 21429.90 (20346.87, 22512.92) | 961.22 (932.46, 990.74) | 1001.73 (939.67, 1063.79) | 5224.40 (5158.42, 5291.12) | 4849.38 (4555.31, 5143.44) |
| Denmark | 5310.93 (5263.47, 5358.78) | 5140.97 (4897.32, 5384.63) | 275.89 (265.52, 286.66) | 248.03 (234.49, 261.58) | 1253.96 (1230.62, 1277.69) | 1317.59 (1240.20, 1394.98) |
| Djibouti | 23107.95 (21807.43, 24474.53) | 25672.85 (24472.64, 26873.06) | 983.40 (725.27, 1313.47) | 1149.76 (1101.77, 1197.74) | 5609.28 (4982.57, 6302.70) | 5976.74 (5681.71, 6271.77) |
| Dominica | 6155.91 (5364.47, 7050.67) | 6584.95 (6516.02, 6653.89) | 243.38 (111.48, 495.94) | 250.72 (247.18, 254.26) | 2020.73 (1574.36, 2575.16) | 2171.77 (2149.32, 2194.21) |
| Dominican Republic | 5515.75 (5450.18, 5582.00) | 4433.04 (4082.52, 4783.57) | 230.49 (217.33, 244.33) | 183.39 (168.54, 198.25) | 1766.95 (1729.81, 1804.77) | 1390.25 (1245.00, 1535.49) |
| Ecuador | 5829.72 (5768.27, 5891.76) | 7886.66 (7171.66, 8601.66) | 248.61 (235.90, 261.91) | 342.52 (303.03, 382.01) | 1849.90 (1815.23, 1885.15) | 2552.97 (2342.92, 2763.01) |
| Egypt | 17506.49 (17409.00, 17604.48) | 18647.89 (17154.88, 20140.89) | 622.69 (604.84, 641.03) | 624.24 (546.89, 701.58) | 5698.50 (5642.55, 5754.96) | 6232.02 (5752.84, 6711.20) |
| El Salvador | 4697.25 (4635.59, 4759.67) | 4683.57 (4510.66, 4856.47) | 216.21 (203.30, 229.89) | 202.38 (189.57, 215.20) | 1489.77 (1455.09, 1525.22) | 1502.40 (1462.74, 1542.07) |
| Equatorial Guinea | 19355.56 (18344.87, 20413.18) | 19088.29 (17853.06, 20323.52) | 865.77 (660.48, 1121.33) | 875.43 (817.42, 933.44) | 4892.62 (4393.35, 5439.32) | 4800.29 (4517.56, 5083.01) |
| Eritrea | 38287.13 (37166.76, 39437.82) | 33225.57 (31345.95, 35105.20) | 1814.76 (1567.43, 2094.94) | 1769.36 (1682.23, 1856.49) | 8868.88 (8339.89, 9428.17) | 7078.70 (6669.45, 7487.94) |
| Estonia | 6704.45 (6583.46, 6827.40) | 697.31 (0.05, 1711.66) | 322.82 (296.66, 350.99) | 51.82 (2.62, 101.03) | 1981.86 (1916.35, 2049.34) | 78.23 (0.05, 368.61) |
| Eswatini | 34125.72 (32024.27, 36347.99) | 52861.67 (49104.71, 56618.63) | 1507.56 (1079.49, 2070.23) | 2569.45 (2387.45, 2751.44) | 8728.26 (7688.63, 9890.35) | 13270.54 (12330.14, 14210.94) |
| Ethiopia | 15849.61 (15758.77, 15940.91) | 10453.31 (9514.84, 11391.77) | 752.32 (732.25, 772.85) | 526.18 (482.54, 569.83) | 3687.58 (3644.00, 3731.61) | 2185.82 (1965.31, 2406.32) |
| Fiji | 25760.09 (24337.07, 27256.56) | 32385.86 (24868.86, 39902.85) | 1126.29 (835.05, 1499.18) | 1608.35 (1193.92, 2022.78) | 7914.44 (7141.25, 8761.55) | 9637.33 (7529.74, 11744.91) |
| Finland | 5496.68 (5453.07, 5540.61) | 4095.66 (3515.20, 4676.11) | 289.66 (279.78, 299.86) | 200.10 (165.54, 234.66) | 1463.70 (1441.19, 1486.52) | 1197.80 (1041.68, 1353.92) |
| France | 4395.90 (4384.75, 4407.07) | 2565.80 (2145.86, 2985.74) | 249.32 (246.58, 252.08) | 155.42 (129.00, 181.83) | 1122.63 (1117.16, 1128.13) | 618.99 (498.83, 739.16) |
| Gabon | 19227.51 (18560.59, 19914.93) | 19344.51 (18490.22, 20198.81) | 894.97 (753.84, 1057.71) | 855.82 (811.85, 899.78) | 4575.24 (4254.05, 4917.07) | 4608.76 (4412.22, 4805.30) |
| Gambia | 11433.42 (10923.90, 11963.13) | 12111.42 (11492.10, 12730.74) | 536.86 (429.61, 665.59) | 594.44 (563.63, 625.25) | 2826.84 (2576.16, 3098.01) | 2610.68 (2464.99, 2756.37) |
| Georgia | 5142.24 (5080.14, 5205.04) | 5780.56 (5067.29, 6493.82) | 245.22 (231.85, 259.30) | 294.17 (257.20, 331.14) | 1504.00 (1470.73, 1537.97) | 1508.71 (1303.05, 1714.37) |
| Germany | 7427.50 (7414.06, 7440.97) | 6440.97 (4918.37, 7963.57) | 369.06 (366.12, 372.02) | 368.38 (285.90, 450.87) | 2292.44 (2284.89, 2300.01) | 1590.26 (1096.21, 2084.32) |
| Ghana | 10945.04 (10815.09, 11076.32) | 11626.61 (11001.62, 12251.60) | 485.26 (457.83, 514.06) | 525.90 (499.73, 552.07) | 2748.26 (2683.42, 2814.45) | 2698.13 (2527.65, 2868.61) |
| Greece | 6170.54 (6136.79, 6204.44) | 634.25 (0.05, 3193.57) | 318.38 (310.75, 326.18) | 35.41 (0.05, 182.27) | 1819.24 (1800.92, 1837.73) | 153.71 (0.05, 856.80) |
| Greenland | 15592.13 (13309.80, 18213.87) | 8597.94 (5449.02, 11746.86) | 616.03 (232.43, 1412.51) | 306.80 (144.55, 469.06) | 5191.29 (3917.47, 6816.19) | 2853.74 (1897.60, 3809.89) |
| Grenada | 7321.32 (6433.01, 8315.33) | 10773.26 (10569.57, 10976.95) | 256.13 (119.74, 512.80) | 369.64 (352.42, 386.86) | 2482.79 (1974.40, 3100.71) | 3669.65 (3621.70, 3717.59) |
| Guam | 5092.13 (4713.54, 5497.85) | 6724.17 (6253.69, 7194.65) | 252.97 (173.10, 363.02) | 319.16 (287.53, 350.79) | 1463.84 (1265.60, 1689.66) | 1877.20 (1740.38, 2014.01) |
| Guatemala | 19986.46 (19680.00, 20297.10) | 0.05 (0.05, 4016.29) | 969.91 (901.83, 1042.36) | 0.05 (0.05, 226.53) | 6396.90 (6225.16, 6572.82) | 0.05 (0.05, 1202.01) |
| Guinea | 6951.39 (6827.25, 7077.47) | 7512.97 (6897.08, 8128.85) | 306.98 (281.47, 334.46) | 290.40 (261.83, 318.96) | 1827.75 (1764.17, 1893.29) | 1936.31 (1776.54, 2096.09) |
| Guinea-Bissau | 17823.21 (16733.02, 18974.72) | 23364.35 (22159.86, 24568.84) | 770.11 (552.11, 1055.76) | 1046.87 (985.92, 1107.82) | 4383.48 (3851.32, 4978.22) | 5650.84 (5351.72, 5949.96) |
| Guyana | 6680.88 (6324.67, 7053.80) | 2693.02 (0.05, 13495.87) | 251.48 (186.90, 333.67) | 0.05 (0.05, 564.05) | 2261.88 (2056.00, 2484.72) | 1377.60 (0.05, 4710.28) |
| Haiti | 11999.49 (11783.78, 12218.65) | 11350.72 (10722.41, 11979.03) | 470.30 (427.54, 516.66) | 429.01 (403.97, 454.06) | 3857.17 (3735.00, 3982.81) | 3583.13 (3370.34, 3795.93) |
| Honduras | 16571.63 (16310.99, 16835.95) | 16618.44 (13752.87, 19484.02) | 741.09 (685.18, 800.89) | 725.89 (592.85, 858.93) | 4657.73 (4520.62, 4798.52) | 4843.62 (4012.86, 5674.39) |
| Hungary | 6014.00 (5971.67, 6056.60) | 1917.71 (552.57, 3282.86) | 265.89 (257.19, 274.85) | 57.01 (0.05, 130.13) | 1871.09 (1847.37, 1895.07) | 572.61 (186.58, 958.64) |
| Iceland | 5154.31 (4950.72, 5365.61) | 4051.82 (3889.32, 4214.31) | 261.84 (217.65, 314.14) | 194.65 (182.93, 206.37) | 1543.21 (1433.14, 1661.05) | 1318.89 (1276.55, 1361.22) |
| India | 9486.71 (9474.32, 9499.12) | 10296.85 (9475.02, 11118.67) | 427.32 (424.66, 430.00) | 468.73 (427.20, 510.25) | 2456.66 (2450.34, 2462.99) | 2625.06 (2423.39, 2826.73) |
| Indonesia | 15739.56 (15695.62, 15783.62) | 18659.10 (17980.05, 19338.16) | 628.94 (619.97, 638.01) | 736.25 (703.14, 769.37) | 4658.25 (4634.45, 4682.16) | 5419.83 (5217.10, 5622.56) |
| Iran (Islamic Republic of) | 9518.48 (9482.22, 9554.86) | 11325.45 (8761.83, 13889.06) | 415.90 (408.37, 423.56) | 525.90 (423.35, 628.45) | 2896.04 (2876.02, 2916.19) | 3489.21 (2555.47, 4422.95) |
| Iraq | 11247.89 (11160.44, 11335.93) | 15302.45 (14413.84, 16191.07) | 445.18 (428.23, 462.73) | 609.35 (571.35, 647.36) | 3539.52 (3490.29, 3589.37) | 4793.73 (4533.19, 5054.26) |
| Ireland | 6232.14 (6162.87, 6302.11) | 5628.29 (5072.96, 6183.62) | 298.36 (283.74, 313.68) | 260.97 (228.58, 293.36) | 1852.67 (1814.48, 1891.58) | 1701.38 (1551.27, 1851.48) |
| Israel | 5588.89 (5539.91, 5638.26) | 4529.37 (3825.22, 5233.53) | 280.97 (270.10, 292.22) | 209.31 (171.13, 247.49) | 1608.19 (1581.94, 1634.83) | 1416.05 (1208.60, 1623.50) |
| Italy | 6619.21 (6605.78, 6632.65) | 4481.70 (3537.52, 5425.88) | 355.95 (352.85, 359.08) | 218.89 (161.70, 276.07) | 1826.31 (1819.27, 1833.37) | 1861.88 (1613.10, 2110.66) |
| Jamaica | 4287.21 (4201.75, 4374.24) | 4787.75 (4639.40, 4936.11) | 208.80 (189.88, 229.37) | 221.24 (210.80, 231.67) | 1344.24 (1297.19, 1392.84) | 1517.59 (1480.56, 1554.62) |
| Japan | 5167.51 (5160.64, 5174.39) | 4424.66 (4242.48, 4606.84) | 285.57 (283.92, 287.22) | 285.27 (271.55, 299.00) | 1384.36 (1380.87, 1387.86) | 1094.63 (1011.35, 1177.91) |
| Jordan | 14151.71 (13936.48, 14369.90) | 16330.77 (15988.10, 16673.43) | 520.03 (480.09, 562.94) | 626.72 (611.15, 642.30) | 4870.43 (4744.18, 4999.67) | 5553.80 (5425.62, 5681.98) |
| Kazakhstan | 13985.70 (13836.86, 14135.95) | 14053.77 (12435.75, 15671.78) | 609.90 (578.01, 643.28) | 623.43 (553.88, 692.98) | 4488.99 (4405.49, 4573.90) | 4171.83 (3593.61, 4750.04) |
| Kenya | 15282.98 (15156.20, 15410.68) | 21551.75 (20832.20, 22271.31) | 661.77 (635.59, 688.87) | 928.88 (893.07, 964.68) | 3726.49 (3664.07, 3789.84) | 5151.56 (4973.65, 5329.47) |
| Kiribati | 26109.22 (21862.66, 31069.35) | 27527.20 (27205.96, 27848.43) | 1127.87 (383.14, 2755.40) | 1157.95 (1139.65, 1176.25) | 7334.65 (5192.58, 10220.68) | 7655.28 (7573.24, 7737.32) |
| Kuwait | 7265.33 (7101.11, 7432.87) | 6668.72 (5540.87, 7796.58) | 302.52 (270.48, 337.91) | 244.67 (191.97, 297.37) | 2358.20 (2264.13, 2455.64) | 2234.83 (1976.97, 2492.70) |
| Kyrgyzstan | 9147.91 (8977.37, 9321.33) | 5273.53 (4079.13, 6467.92) | 391.98 (356.62, 430.37) | 182.49 (135.36, 229.62) | 2837.55 (2743.11, 2934.89) | 1463.49 (1057.69, 1869.28) |
| Lao People's Democratic Republic | 12115.33 (11882.57, 12352.02) | 11821.82 (11186.33, 12457.30) | 481.53 (435.65, 531.47) | 441.62 (410.14, 473.10) | 3623.48 (3497.00, 3753.90) | 3615.10 (3443.34, 3786.86) |
| Latvia | 6626.65 (6528.34, 6726.24) | 4279.76 (3580.34, 4979.18) | 305.41 (284.67, 327.47) | 223.17 (186.80, 259.54) | 2064.20 (2009.39, 2120.30) | 1251.31 (1055.96, 1446.65) |
| Lebanon | 6974.56 (6893.61, 7056.37) | 3495.87 (3105.82, 3885.92) | 306.82 (290.30, 324.20) | 146.57 (119.27, 173.87) | 2296.63 (2250.02, 2344.10) | 1121.39 (1008.03, 1234.76) |
| Lesotho | 23987.59 (23033.91, 24974.82) | 37719.33 (35751.89, 39686.77) | 1023.23 (830.45, 1251.83) | 1703.63 (1615.12, 1792.14) | 6422.88 (5935.90, 6943.66) | 9849.16 (9306.20, 10392.13) |
| Liberia | 7699.95 (7444.82, 7962.62) | 8001.12 (7477.32, 8524.93) | 334.36 (282.87, 393.68) | 338.66 (311.31, 366.00) | 2059.23 (1928.09, 2197.99) | 2166.10 (2068.39, 2263.81) |
| Libya | 9148.52 (9010.16, 9288.85) | 8148.70 (7740.22, 8557.18) | 389.80 (362.65, 418.89) | 352.98 (330.86, 375.09) | 2681.52 (2605.96, 2759.08) | 2357.59 (2264.91, 2450.26) |
| Lithuania | 7703.83 (7604.11, 7804.73) | 1579.84 (1205.22, 1954.46) | 369.57 (347.78, 392.56) | 76.24 (49.93, 102.56) | 2333.13 (2278.45, 2388.99) | 378.24 (254.11, 502.37) |
| Luxembourg | 4078.78 (3940.86, 4221.09) | 2407.83 (2002.80, 2812.87) | 206.08 (176.28, 240.42) | 123.50 (105.04, 141.96) | 1217.86 (1142.78, 1297.40) | 688.57 (519.91, 857.22) |
| Madagascar | 14256.27 (14044.59, 14470.68) | 17306.81 (16495.81, 18117.81) | 544.52 (503.12, 588.76) | 631.41 (593.40, 669.42) | 4293.25 (4177.94, 4411.31) | 5148.12 (4901.78, 5394.46) |
| Malawi | 15743.92 (15510.36, 15980.46) | 17902.82 (16862.42, 18943.23) | 670.52 (622.76, 721.33) | 743.81 (696.19, 791.42) | 3966.36 (3849.85, 4085.86) | 4389.50 (4122.41, 4656.58) |
| Malaysia | 12930.29 (12838.58, 13022.60) | 6349.65 (5725.84, 6973.46) | 553.22 (533.82, 573.22) | 342.49 (282.35, 402.62) | 3521.47 (3474.10, 3569.42) | 1441.92 (1294.61, 1589.23) |
| Maldives | 8752.41 (8160.04, 9382.10) | 2850.80 (2354.63, 3346.98) | 366.95 (252.78, 522.18) | 76.26 (42.24, 110.28) | 2728.21 (2402.83, 3091.48) | 989.10 (836.25, 1141.96) |
| Mali | 14969.72 (14736.29, 15206.29) | 19834.11 (17389.19, 22279.03) | 693.27 (642.98, 746.81) | 1047.82 (904.59, 1191.05) | 3508.56 (3396.21, 3624.06) | 4533.78 (3953.84, 5113.72) |
| Malta | 5930.93 (5749.76, 6117.43) | 6219.85 (5611.16, 6828.55) | 283.16 (245.81, 325.91) | 296.93 (261.87, 332.00) | 1767.37 (1667.57, 1872.63) | 1880.51 (1734.47, 2026.55) |
| Marshall Islands | 27952.31 (20742.46, 37252.22) | 39205.66 (38719.17, 39692.15) | 1114.14 (120.08, 4850.34) | 1562.98 (1533.93, 1592.02) | 7791.71 (4256.43, 13576.45) | 11082.09 (10929.91, 11234.28) |
| Mauritania | 7943.20 (7711.38, 8181.37) | 10568.68 (9382.90, 11754.45) | 347.83 (301.38, 400.78) | 490.44 (423.64, 557.24) | 2030.80 (1914.07, 2153.97) | 2584.69 (2283.27, 2886.10) |
| Mauritius | 6789.80 (6612.57, 6971.30) | 6652.06 (5953.09, 7351.04) | 266.70 (233.89, 303.77) | 237.72 (200.79, 274.64) | 2061.11 (1963.32, 2163.23) | 2168.68 (2043.23, 2294.12) |
| Mexico | 5606.12 (5585.90, 5626.40) | 7811.88 (6962.73, 8661.03) | 220.06 (216.14, 224.05) | 314.65 (282.27, 347.03) | 1925.04 (1913.14, 1937.00) | 2760.20 (2401.02, 3119.37) |
| Micronesia (Federated States of) | 16325.70 (13853.80, 19178.43) | 16755.51 (16141.30, 17369.72) | 690.65 (258.73, 1585.88) | 626.28 (598.56, 654.01) | 4617.76 (3353.75, 6279.66) | 4935.79 (4782.23, 5089.36) |
| Monaco | 5233.81 (4786.07, 5719.89) | 5133.87 (4773.73, 5494.01) | 285.42 (189.10, 424.22) | 247.02 (219.98, 274.05) | 1422.78 (1193.52, 1691.67) | 1515.44 (1430.44, 1600.43) |
| Mongolia | 24064.74 (23234.76, 24920.93) | 20869.89 (18069.76, 23670.01) | 1118.81 (939.92, 1325.99) | 1114.75 (956.37, 1273.13) | 7484.09 (7029.33, 7965.01) | 5868.06 (5105.31, 6630.82) |
| Montenegro | 12824.82 (12344.68, 13320.91) | 23588.14 (22960.55, 24215.73) | 561.71 (463.50, 676.83) | 1092.24 (1058.52, 1125.97) | 4080.21 (3811.80, 4364.68) | 7279.84 (7089.09, 7470.59) |
| Morocco | 13708.87 (13626.88, 13791.28) | 16296.65 (15800.82, 16792.47) | 602.47 (585.22, 620.14) | 759.07 (736.43, 781.71) | 4017.87 (3973.63, 4062.52) | 4640.28 (4497.93, 4782.64) |
| Mozambique | 17193.85 (16983.71, 17406.23) | 24089.40 (23399.31, 24779.49) | 743.42 (700.26, 788.85) | 1040.63 (1005.50, 1075.76) | 4164.08 (4061.09, 4269.34) | 5794.87 (5631.06, 5958.67) |
| Myanmar | 10185.50 (10129.37, 10241.90) | 7795.07 (7266.74, 8323.39) | 420.18 (408.68, 431.95) | 267.31 (242.94, 291.69) | 3036.82 (3006.29, 3067.61) | 2281.15 (2100.77, 2461.54) |
| Namibia | 16216.91 (15656.55, 16794.40) | 17048.06 (16065.57, 18030.55) | 708.69 (593.65, 841.85) | 732.16 (685.37, 778.96) | 4185.66 (3904.27, 4484.29) | 4265.69 (4007.39, 4524.00) |
| Nauru | 25101.43 (12258.03, 48046.05) | 26204.26 (25620.62, 26787.90) | 1070.25 (0.26, 15508.58) | 1031.64 (987.28, 1076.01) | 7068.67 (1445.35, 24091.71) | 7648.02 (7546.05, 7749.98) |
| Nepal | 8984.90 (8902.13, 9068.33) | 11288.78 (11004.56, 11573.00) | 377.96 (361.13, 395.46) | 488.02 (474.16, 501.88) | 2554.09 (2509.98, 2598.86) | 3089.31 (2997.08, 3181.54) |
| Netherlands | 6715.52 (6683.61, 6747.57) | 6740.54 (6416.33, 7064.75) | 329.72 (322.84, 336.73) | 320.62 (305.44, 335.80) | 1937.25 (1919.88, 1954.76) | 1975.95 (1865.37, 2086.53) |
| New Zealand | 6330.56 (6265.33, 6396.39) | 5359.30 (5103.27, 5615.32) | 295.47 (281.80, 309.73) | 239.28 (223.62, 254.93) | 1862.46 (1826.96, 1898.57) | 1704.15 (1639.30, 1769.00) |
| Nicaragua | 8372.56 (8222.23, 8525.37) | 10234.00 (10006.20, 10461.81) | 349.67 (318.86, 383.09) | 435.88 (423.93, 447.83) | 2816.93 (2730.27, 2906.08) | 3491.66 (3407.13, 3576.19) |
| Niger | 11910.22 (11708.47, 12114.94) | 13090.35 (12334.87, 13845.83) | 494.23 (453.31, 538.22) | 504.84 (466.03, 543.66) | 3080.43 (2978.43, 3185.41) | 3338.40 (3139.09, 3537.72) |
| Nigeria | 7331.93 (7295.73, 7368.30) | 7818.47 (7522.22, 8114.72) | 325.53 (318.03, 333.18) | 338.27 (324.50, 352.04) | 1792.78 (1774.84, 1810.88) | 1904.16 (1829.42, 1978.90) |
| Niue | 10062.96 (4135.17, 21768.59) | 8471.20 (7773.70, 9168.71) | 481.51 (0.02, 8329.84) | 403.19 (364.91, 441.46) | 2692.98 (313.50, 11639.53) | 2301.91 (2136.59, 2467.23) |
| North Macedonia | 209866.67 (202723.55, 217211.15) | 260543.83 (202167.92, 318919.74) | 10953.85 (9371.61, 12742.62) | 14198.94 (11066.80, 17331.09) | 63943.36 (60017.07, 68072.98) | 76732.27 (58645.26, 94819.29) |
| Northern Mariana Islands | 13925.66 (11325.65, 17036.70) | 17851.11 (15366.04, 20336.17) | 549.53 (144.48, 1593.23) | 709.14 (599.16, 819.13) | 4073.24 (2732.68, 5952.23) | 5188.25 (4562.06, 5814.45) |
| Norway | 5220.61 (5172.05, 5269.60) | 5369.71 (5246.51, 5492.90) | 269.22 (258.33, 280.53) | 241.55 (235.39, 247.71) | 1462.61 (1437.04, 1488.60) | 1694.53 (1636.75, 1752.32) |
| Oman | 14390.88 (13958.07, 14835.46) | 24466.02 (23279.18, 25652.87) | 569.73 (486.82, 664.81) | 1017.10 (962.30, 1071.90) | 4358.37 (4121.79, 4606.80) | 7320.10 (6983.02, 7657.17) |
| Pakistan | 9045.05 (9007.95, 9082.28) | 10724.13 (10236.32, 11211.95) | 396.50 (388.79, 404.34) | 471.45 (444.02, 498.87) | 2308.11 (2289.39, 2326.95) | 2676.33 (2556.80, 2795.86) |
| Palau | 18638.23 (12797.76, 26881.31) | 19503.77 (17429.93, 21577.60) | 833.48 (43.86, 4950.76) | 707.37 (596.59, 818.15) | 5386.96 (2543.88, 10876.93) | 6078.68 (5562.44, 6594.91) |
| Palestine | 15647.39 (15242.77, 16061.47) | 4414.60 (2324.32, 6504.89) | 651.64 (569.36, 743.92) | 81.93 (0.05, 187.05) | 4997.69 (4772.52, 5232.27) | 1818.56 (1240.64, 2396.47) |
| Panama | 4907.73 (4826.67, 4990.03) | 4967.52 (4879.58, 5055.46) | 209.86 (193.84, 227.12) | 211.13 (207.31, 214.96) | 1576.53 (1530.56, 1623.75) | 1604.90 (1577.55, 1632.25) |
| Papua New Guinea | 14351.66 (14056.83, 14651.74) | 16548.60 (14776.81, 18320.39) | 537.40 (480.88, 599.38) | 548.70 (458.39, 639.01) | 4465.42 (4301.55, 4634.58) | 5186.43 (4631.08, 5741.79) |
| Paraguay | 7595.01 (7491.81, 7699.54) | 8990.06 (8810.67, 9169.44) | 331.59 (311.30, 353.16) | 370.09 (359.24, 380.94) | 2030.55 (1976.67, 2085.77) | 2439.10 (2400.19, 2478.00) |
| Peru | 3762.10 (3736.62, 3787.73) | 4144.42 (4009.70, 4279.15) | 167.22 (162.00, 172.59) | 178.23 (172.55, 183.91) | 1111.13 (1097.31, 1125.12) | 1204.00 (1154.89, 1253.10) |
| Philippines | 10321.79 (10277.65, 10366.10) | 13320.12 (1900.77, 24739.47) | 408.00 (399.28, 416.88) | 401.83 (0.05, 1170.18) | 3064.19 (3040.18, 3088.35) | 4483.43 (1436.89, 7529.98) |
| Poland | 6188.31 (6165.44, 6211.26) | 1444.00 (0.05, 3397.93) | 284.33 (279.46, 289.27) | 29.66 (0.05, 133.42) | 1869.71 (1857.11, 1882.39) | 399.87 (0.05, 997.23) |
| Portugal | 6096.32 (6062.54, 6130.27) | 5130.61 (4230.98, 6030.24) | 311.19 (303.64, 318.91) | 284.13 (230.07, 338.19) | 1759.45 (1741.27, 1777.81) | 1391.72 (1174.31, 1609.13) |
| Puerto Rico | 3987.11 (3938.13, 4036.63) | 3089.36 (2413.55, 3765.17) | 188.82 (178.30, 199.90) | 151.42 (112.01, 190.83) | 1256.01 (1228.59, 1283.98) | 1011.67 (831.79, 1191.56) |
| Qatar | 25286.51 (23805.91, 26847.71) | 0.05 (0.05, 7354.76) | 1004.38 (717.04, 1382.11) | 0.05 (0.05, 447.36) | 8158.91 (7340.14, 9058.17) | 0.05 (0.05, 1791.62) |
| Republic of Korea | 9923.99 (9892.48, 9955.58) | 3802.00 (1392.15, 6211.84) | 460.29 (453.60, 467.06) | 182.12 (52.25, 311.98) | 2785.20 (2768.47, 2802.03) | 933.36 (361.70, 1505.02) |
| Republic of Moldova | 5653.67 (5578.33, 5729.95) | 1131.53 (0.05, 4029.35) | 239.90 (225.02, 255.70) | 39.76 (0.05, 193.11) | 1776.81 (1734.53, 1820.03) | 266.82 (0.05, 1076.22) |
| Romania | 7515.57 (7475.97, 7555.35) | 0.05 (0.05, 2897.19) | 342.19 (333.74, 350.82) | 0.05 (0.05, 148.65) | 2317.86 (2295.85, 2340.06) | 0.05 (0.05, 842.34) |
| Russian Federation | 8035.30 (8017.83, 8052.81) | 1208.33 (0.05, 3080.76) | 365.35 (361.58, 369.14) | 48.61 (0.05, 134.17) | 2454.45 (2444.79, 2464.15) | 245.33 (0.05, 800.20) |
| Rwanda | 17371.38 (17091.18, 17655.51) | 17841.62 (16339.27, 19343.97) | 765.24 (706.69, 827.84) | 787.29 (734.56, 840.02) | 4245.06 (4107.63, 4386.41) | 4233.84 (3839.88, 4627.80) |
| Saint Kitts and Nevis | 6817.03 (5709.25, 8106.89) | 8305.23 (7310.39, 9300.07) | 250.79 (82.96, 636.87) | 239.57 (183.60, 295.54) | 2199.86 (1588.81, 3001.55) | 2788.80 (2467.88, 3109.72) |
| Saint Lucia | 5731.42 (5290.34, 6204.47) | 8275.02 (0.05, 18594.84) | 240.21 (156.92, 358.77) | 397.37 (0.05, 952.51) | 1865.35 (1617.71, 2145.54) | 2484.54 (0.05, 5538.10) |
| Saint Vincent and the Grenadines | 8919.60 (7967.54, 9969.66) | 9998.29 (8551.82, 11444.75) | 379.03 (202.55, 668.26) | 447.89 (371.50, 524.28) | 2935.38 (2401.22, 3570.05) | 3138.60 (2750.44, 3526.75) |
| Samoa | 12234.77 (11068.21, 13507.93) | 9920.80 (9616.20, 10225.40) | 567.80 (336.55, 919.94) | 397.48 (374.44, 420.51) | 3169.59 (2591.54, 3857.32) | 2725.38 (2639.95, 2810.81) |
| San Marino | 4484.35 (4018.28, 5000.06) | 3901.23 (3744.37, 4058.09) | 268.00 (159.33, 434.97) | 218.71 (206.26, 231.17) | 1204.18 (978.49, 1479.53) | 1089.29 (1021.11, 1157.48) |
| Sao Tome and Principe | 8764.94 (7549.68, 10146.40) | 9917.61 (9710.17, 10125.05) | 403.41 (176.83, 825.76) | 467.70 (457.44, 477.95) | 2269.68 (1672.64, 3040.63) | 2462.94 (2414.70, 2511.17) |
| Saudi Arabia | 14616.14 (14456.41, 14777.44) | 16274.33 (15236.11, 17312.56) | 564.51 (532.90, 597.74) | 486.56 (432.69, 540.43) | 4489.62 (4401.98, 4578.82) | 5407.70 (5167.08, 5648.33) |
| Senegal | 9063.87 (8925.18, 9204.40) | 9599.00 (8933.82, 10264.17) | 412.30 (383.05, 443.43) | 425.96 (392.68, 459.25) | 2154.29 (2086.81, 2223.65) | 2198.83 (2030.25, 2367.40) |
| Serbia | 6280.03 (6229.70, 6330.75) | 21578.48 (12636.91, 30520.06) | 259.75 (250.02, 269.85) | 842.39 (572.30, 1112.49) | 2007.25 (1978.54, 2036.35) | 7464.51 (4154.39, 10774.63) |
| Seychelles | 7846.75 (7016.29, 8762.46) | 7968.48 (7852.36, 8084.60) | 322.85 (175.25, 566.76) | 302.08 (296.42, 307.73) | 2354.44 (1909.24, 2887.61) | 2455.56 (2422.03, 2489.09) |
| Sierra Leone | 8966.63 (8747.65, 9190.34) | 10601.83 (9867.01, 11336.66) | 377.22 (333.30, 426.02) | 466.64 (429.63, 503.65) | 2562.00 (2445.47, 2683.29) | 2928.56 (2715.36, 3141.75) |
| Singapore | 5110.70 (5042.55, 5179.68) | 3851.96 (3576.44, 4127.48) | 217.75 (204.35, 231.95) | 170.18 (154.33, 186.02) | 1634.02 (1595.10, 1673.79) | 1372.31 (1306.75, 1437.87) |
| Slovakia | 6418.23 (6348.49, 6488.68) | 6789.62 (5849.37, 7729.88) | 271.43 (257.73, 285.83) | 267.57 (217.38, 317.76) | 1997.11 (1957.86, 2037.10) | 2131.28 (1869.04, 2393.52) |
| Slovenia | 5256.56 (5182.97, 5331.08) | 2303.32 (1735.08, 2871.56) | 249.50 (233.83, 266.12) | 113.97 (86.95, 141.00) | 1565.72 (1525.59, 1606.79) | 640.58 (442.97, 838.19) |
| Solomon Islands | 25275.96 (23256.45, 27450.20) | 15629.17 (14623.36, 16634.99) | 1143.01 (733.25, 1729.70) | 643.59 (584.56, 702.63) | 7189.83 (6138.02, 8399.35) | 4685.34 (4467.16, 4903.53) |
| Somalia | 44655.82 (43714.06, 45616.26) | 38435.79 (32278.94, 44592.64) | 1910.24 (1710.27, 2130.44) | 1678.25 (1394.64, 1961.86) | 11324.74 (10856.69, 11811.46) | 9774.36 (8192.56, 11356.17) |
| South Africa | 12341.26 (12268.28, 12414.63) | 11964.52 (10747.45, 13181.58) | 565.31 (549.33, 581.69) | 497.52 (424.09, 570.95) | 3511.49 (3472.69, 3550.68) | 3459.31 (3134.32, 3784.30) |
| South Sudan | 10791.57 (10563.99, 11023.53) | 16372.83 (15787.64, 16958.02) | 441.74 (397.43, 490.48) | 670.42 (644.49, 696.36) | 2858.22 (2741.77, 2979.07) | 4249.08 (4105.44, 4392.72) |
| Spain | 5170.85 (5156.38, 5185.36) | 3015.46 (2688.47, 3342.46) | 287.38 (283.94, 290.87) | 174.25 (152.77, 195.73) | 1276.11 (1268.98, 1283.28) | 780.44 (706.76, 854.13) |
| Sri Lanka | 9997.63 (9923.54, 10072.20) | 10316.90 (8995.96, 11637.83) | 397.40 (382.65, 412.62) | 430.80 (362.39, 499.21) | 3066.93 (3026.03, 3108.30) | 3196.50 (2814.61, 3578.39) |
| Sudan | 9942.12 (9858.21, 10026.68) | 12897.86 (12500.96, 13294.76) | 392.57 (376.45, 409.31) | 518.00 (497.30, 538.71) | 3175.10 (3127.42, 3223.43) | 4114.36 (4006.80, 4221.92) |
| Suriname | 5658.79 (5382.81, 5948.11) | 5487.95 (5268.08, 5707.83) | 220.10 (170.79, 283.31) | 217.94 (206.69, 229.19) | 1908.71 (1746.99, 2084.22) | 1805.15 (1721.79, 1888.51) |
| Sweden | 4920.45 (4890.13, 4950.94) | 5298.45 (5168.70, 5428.19) | 239.29 (232.78, 245.97) | 213.77 (204.93, 222.61) | 1598.54 (1581.20, 1616.05) | 2026.63 (1973.34, 2079.93) |
| Switzerland | 4985.84 (4952.71, 5019.19) | 4871.23 (4735.98, 5006.47) | 265.64 (258.04, 273.44) | 249.73 (238.94, 260.51) | 1388.37 (1370.93, 1406.01) | 1416.30 (1386.84, 1445.77) |
| Syrian Arab Republic | 27433.32 (27107.69, 27762.39) | 12870.36 (9120.60, 16620.12) | 1214.69 (1144.88, 1288.10) | 513.67 (322.36, 704.99) | 8533.51 (8354.70, 8715.72) | 4121.93 (3090.80, 5153.06) |
| Taiwan (Province of China) | 4485.54 (4461.02, 4510.19) | 5328.53 (4912.12, 5744.94) | 202.05 (196.99, 207.22) | 288.11 (264.48, 311.74) | 1387.30 (1373.63, 1401.09) | 1141.95 (980.71, 1303.19) |
| Tajikistan | 9346.96 (9181.42, 9515.09) | 6925.23 (5502.27, 8348.20) | 397.05 (363.18, 433.60) | 273.11 (218.72, 327.50) | 2729.54 (2640.32, 2821.37) | 1795.04 (1340.53, 2249.56) |
| Thailand | 6400.92 (6380.66, 6421.23) | 3663.05 (3048.53, 4277.57) | 273.19 (269.14, 277.30) | 172.60 (143.48, 201.71) | 1724.00 (1713.50, 1734.57) | 917.04 (720.38, 1113.69) |
| Timor-Leste | 15283.00 (14601.07, 15992.78) | 12650.80 (11617.33, 13684.26) | 616.44 (483.03, 780.00) | 458.29 (425.93, 490.66) | 4688.16 (4314.73, 5089.72) | 4218.07 (3874.49, 4561.65) |
| Togo | 13652.19 (13296.78, 14015.71) | 14883.19 (14159.08, 15607.29) | 574.61 (502.59, 655.10) | 641.77 (607.79, 675.74) | 3462.99 (3285.29, 3648.85) | 3673.29 (3504.33, 3842.25) |
| Tokelau | 9592.41 (3222.11, 24129.51) | 3162.84 (1125.30, 5200.37) | 471.40 (0.00, 11209.04) | 89.24 (0.05, 214.36) | 2469.59 (143.68, 14154.19) | 839.60 (390.29, 1288.90) |
| Tonga | 8298.48 (7382.19, 9314.50) | 8191.25 (7509.48, 8873.02) | 382.13 (209.42, 667.59) | 394.10 (355.55, 432.66) | 2248.02 (1781.67, 2818.27) | 2220.34 (2056.60, 2384.08) |
| Trinidad and Tobago | 5354.82 (5206.67, 5506.80) | 6428.15 (5222.17, 7634.14) | 204.94 (177.96, 235.75) | 228.85 (147.99, 309.72) | 1814.75 (1727.79, 1905.62) | 2301.98 (1980.62, 2623.33) |
| Tunisia | 10302.46 (10209.97, 10395.70) | 9546.73 (8900.49, 10192.97) | 434.60 (416.07, 453.89) | 368.37 (328.94, 407.80) | 3147.25 (3095.94, 3199.32) | 3043.42 (2710.88, 3375.97) |
| Turkmenistan | 6022.13 (5903.78, 6142.61) | 5008.20 (4124.12, 5892.29) | 244.73 (222.29, 269.27) | 204.05 (166.13, 241.97) | 1845.43 (1779.91, 1913.10) | 1340.88 (1092.46, 1589.31) |
| Tuvalu | 16077.73 (10527.89, 24017.73) | 12100.89 (10381.95, 13819.84) | 712.25 (23.97, 4590.84) | 401.08 (300.75, 501.41) | 4383.36 (1793.73, 9606.46) | 3618.12 (3250.74, 3985.50) |
| Uganda | 13419.06 (13278.43, 13560.99) | 13109.67 (12669.32, 13550.02) | 585.91 (556.86, 616.27) | 603.61 (584.17, 623.05) | 3427.37 (3356.63, 3499.40) | 3266.56 (3147.00, 3386.12) |
| Ukraine | 9033.83 (8999.78, 9068.00) | 2464.35 (383.33, 4545.37) | 410.76 (403.44, 418.19) | 4.05 (0.05, 122.77) | 2793.58 (2774.65, 2812.62) | 890.27 (293.59, 1486.96) |
| United Arab Emirates | 55200.90 (53258.42, 57212.59) | 82789.36 (79632.65, 85946.07) | 1840.67 (1475.57, 2285.16) | 2435.50 (2319.34, 2551.66) | 17278.39 (16224.27, 18401.07) | 27083.35 (26026.41, 28140.29) |
| United Kingdom | 5253.71 (5239.89, 5267.55) | 5666.96 (5266.38, 6067.55) | 266.29 (263.21, 269.40) | 270.57 (248.63, 292.52) | 1558.46 (1550.93, 1566.03) | 1803.52 (1675.25, 1931.79) |
| United Republic of Tanzania | 12158.19 (12065.05, 12251.95) | 11699.95 (11165.51, 12234.38) | 544.94 (525.34, 565.17) | 561.22 (533.34, 589.10) | 2980.66 (2934.76, 3027.19) | 2717.37 (2587.76, 2846.98) |
| United States of America | 6804.03 (6796.26, 6811.81) | 7112.17 (6938.48, 7285.86) | 325.92 (324.26, 327.59) | 309.59 (299.04, 320.14) | 1870.08 (1865.99, 1874.17) | 2032.30 (1982.08, 2082.52) |
| United States Virgin Islands | 8055.21 (7345.70, 8822.14) | 8358.22 (7621.30, 9095.15) | 318.56 (190.35, 510.59) | 320.27 (263.89, 376.65) | 2742.12 (2335.66, 3207.00) | 3039.26 (2888.18, 3190.34) |
| Uruguay | 4866.54 (4804.89, 4928.93) | 4111.12 (3771.46, 4450.79) | 239.70 (226.32, 253.82) | 206.10 (188.64, 223.56) | 1455.62 (1421.88, 1490.09) | 1213.67 (1103.49, 1323.85) |
| Uzbekistan | 9062.05 (8985.04, 9139.64) | 6149.68 (5815.11, 6484.25) | 372.92 (357.19, 389.24) | 235.18 (224.68, 245.69) | 2830.74 (2787.76, 2874.31) | 1762.04 (1625.84, 1898.24) |
| Vanuatu | 22334.76 (19831.35, 25118.45) | 19654.75 (18452.01, 20857.49) | 963.98 (489.36, 1772.42) | 828.53 (772.38, 884.68) | 6249.77 (4968.03, 7820.52) | 5669.23 (5418.11, 5920.36) |
| Venezuela (Bolivarian Republic of) | 6267.70 (6227.89, 6307.74) | 7697.75 (7422.98, 7972.52) | 254.38 (246.75, 262.22) | 289.70 (276.85, 302.55) | 2025.38 (2002.57, 2048.41) | 2445.90 (2354.95, 2536.85) |
| Viet Nam | 10267.76 (10232.14, 10303.48) | 8763.04 (8539.44, 8986.64) | 463.90 (456.40, 471.52) | 358.62 (347.11, 370.12) | 2554.80 (2537.02, 2572.68) | 2137.91 (2073.51, 2202.32) |
| Yemen | 15060.44 (14906.72, 15215.53) | 16699.17 (15982.41, 17415.93) | 606.75 (576.18, 638.71) | 665.08 (632.88, 697.28) | 4666.12 (4580.72, 4752.88) | 5161.63 (4979.82, 5343.43) |
| Zambia | 18530.84 (18220.91, 18845.34) | 19725.40 (18359.95, 21090.85) | 819.09 (753.54, 889.42) | 855.34 (788.84, 921.83) | 4844.11 (4687.11, 5005.67) | 5176.28 (4846.22, 5506.34) |
| Zimbabwe | 21080.52 (20721.65, 21444.85) | 32932.49 (31179.54, 34685.44) | 889.07 (814.64, 969.23) | 1386.85 (1310.11, 1463.58) | 5573.66 (5390.75, 5762.01) | 8449.18 (7959.64, 8938.73) |

**STable 8. Top and Buttom 10 countries of the projected EAPC of age-standardized rates of Alzheimer's Disease from 2022 to 2030.**

| Top |  |  |  |  |  |  |
| --- | --- | --- | --- | --- | --- | --- |
| Rank | DALYs (Disability-Adjusted Life Years) Country | DALYs (Disability-Adjusted Life Years) Value | Deaths Country | Deaths Value | Incidence Country | Incidence Value |
| 1 | Cyprus | 12.5532 (11.2142, 13.9082) | Cyprus | 12.8393 (11.4379, 14.2584) | Cyprus | 12.1836 (10.9232, 13.4584) |
| 2 | Serbia | 9.6416 (8.8556, 10.4333) | Serbia | 9.4331 (8.6809, 10.1904) | Serbia | 10.0610 (9.2046, 10.9241) |
| 3 | Montenegro | 5.1585 (4.9347, 5.3828) | Montenegro | 5.6008 (5.3368, 5.8654) | Andorra | 5.8660 (5.5764, 6.1564) |
| 4 | Andorra | 5.1059 (4.8866, 5.3256) | Cuba | 5.5482 (5.2892, 5.8078) | Cuba | 5.0410 (4.8273, 5.2551) |
| 5 | Cuba | 4.9480 (4.7421, 5.1542) | Oman | 4.4384 (4.2728, 4.6042) | Montenegro | 4.9177 (4.7143, 5.1214) |
| 6 | Oman | 4.0973 (3.9562, 4.2385) | Andorra | 4.1747 (4.0283, 4.3214) | Oman | 4.0385 (3.9014, 4.1757) |
| 7 | United Arab Emirates | 3.6218 (3.5116, 3.7321) | Eswatini | 4.0242 (3.8881, 4.1605) | United Arab Emirates | 3.9547 (3.8233, 4.0863) |
| 8 | Lesotho | 3.5503 (3.4444, 3.6563) | Lesotho | 4.0070 (3.8721, 4.1421) | Philippines | 3.3951 (3.2983, 3.4921) |
| 9 | Zimbabwe | 3.5224 (3.4182, 3.6267) | Saint Lucia | 3.9841 (3.8507, 4.1177) | Grenada | 3.3295 (3.2364, 3.4227) |
| 10 | South Sudan | 3.4138 (3.3159, 3.5118) | North Macedonia | 3.9028 (3.7748, 4.0310) | Lesotho | 3.3252 (3.2324, 3.4182) |
|  |  |  |  |  |  |  |
|  |  |  |  |  |  |  |
| Bottom |  |  |  |  |  |  |
| Rank | DALYs (Disability-Adjusted Life Years) Country | DALYs (Disability-Adjusted Life Years) Value | Deaths Country | Deaths Value | Incidence Country | Incidence Value |
| 1 | Bahrain | -87.2833 (-94.6637, -69.6952) | Qatar | -76.5709 (-85.5561, -61.9964) | Bahrain | -86.9348 (-93.8452, -72.2653) |
| 2 | Armenia | -85.4069 (-92.8028, -70.4109) | Armenia | -75.6245 (-84.8331, -60.8247) | Qatar | -82.5664 (-91.4038, -64.6436) |
| 3 | Qatar | -85.3979 (-93.1383, -68.9255) | Romania | -73.6195 (-82.0800, -61.1646) | Armenia | -81.9912 (-90.0409, -67.4353) |
| 4 | Romania | -84.4990 (-92.0805, -69.6595) | Guatemala | -70.8300 (-82.4232, -51.5902) | Romania | -81.0489 (-89.3452, -66.2927) |
| 5 | Guatemala | -78.3742 (-90.6146, -50.1698) | Guyana | -69.9293 (-80.4358, -53.7805) | Guatemala | -75.1766 (-88.3065, -47.3039) |
| 6 | Estonia | -20.7481 (-24.5402, -16.7655) | Bahrain | -61.5585 (-78.9395, -29.8334) | Estonia | -27.3273 (-34.1493, -19.7985) |
| 7 | Greece | -20.5512 (-24.2679, -16.6521) | Ukraine | -34.4860 (-45.5510, -21.1725) | Greece | -21.9870 (-26.2734, -17.4514) |
| 8 | Russian Federation | -17.5781 (-20.2597, -14.8064) | Poland | -20.4945 (-24.1896, -16.6192) | Russian Federation | -20.8225 (-24.6432, -16.8080) |
| 9 | Republic of Moldova | -15.2461 (-17.2452, -13.1988) | Greece | -19.9498 (-23.4416, -16.2987) | Republic of Moldova | -17.7240 (-20.4519, -14.9025) |
| 10 | Lithuania | -15.0706 (-17.0227, -13.0726) | Palestine | -19.1770 (-22.3916, -15.8294) | Lithuania | -17.0871 (-19.6158, -14.4789) |

**STable 9. Top and Buttom 10 countries of the projected age-standardized rates of Alzheimer's Disease in 2030.**

| Top |  |  |  |  |  |  |
| --- | --- | --- | --- | --- | --- | --- |
| Rank | DALYs (Disability-Adjusted Life Years) Country | DALYs (Disability-Adjusted Life Years) Value | Deaths Country | Deaths Value | Incidence Country | Incidence Value |
| 1 | Cyprus | 296472.95 (127878.51, 465067.40) | Cyprus | 17357.37 (7484.31, 27230.43) | North Macedonia | 76732.27 (58645.26, 94819.29) |
| 2 | North Macedonia | 260543.83 (202167.92, 318919.74) | North Macedonia | 14198.94 (11066.80, 17331.09) | Cyprus | 76644.31 (35594.75, 117693.87) |
| 3 | United Arab Emirates | 82789.36 (79632.65, 85946.07) | Eswatini | 2569.45 (2387.45, 2751.44) | United Arab Emirates | 27083.35 (26026.41, 28140.29) |
| 4 | Eswatini | 52861.67 (49104.71, 56618.63) | Brunei Darussalam | 2529.95 (2117.29, 2942.60) | Brunei Darussalam | 14678.66 (14056.16, 15301.16) |
| 5 | Central African Republic | 52156.45 (47941.08, 56371.83) | United Arab Emirates | 2435.50 (2319.34, 2551.66) | Eswatini | 13270.54 (12330.14, 14210.94) |
| 6 | Brunei Darussalam | 50716.72 (43843.82, 57589.62) | Central African Republic | 2348.17 (2173.98, 2522.37) | Central African Republic | 13262.81 (12139.08, 14386.54) |
| 7 | Marshall Islands | 39205.66 (38719.17, 39692.15) | Eritrea | 1769.36 (1682.23, 1856.49) | Marshall Islands | 11082.09 (10929.91, 11234.28) |
| 8 | Somalia | 38435.79 (32278.94, 44592.64) | Lesotho | 1703.63 (1615.12, 1792.14) | Lesotho | 9849.16 (9306.20, 10392.13) |
| 9 | Lesotho | 37719.33 (35751.89, 39686.77) | Somalia | 1678.25 (1394.64, 1961.86) | Somalia | 9774.36 (8192.56, 11356.17) |
| 10 | Eritrea | 33225.57 (31345.95, 35105.20) | Fiji | 1608.35 (1193.92, 2022.78) | Fiji | 9637.33 (7529.74, 11744.91) |
|  |  |  |  |  |  |  |
|  |  |  |  |  |  |  |
| Bottom |  |  |  |  |  |  |
| Rank | DALYs (Disability-Adjusted Life Years) Country | DALYs (Disability-Adjusted Life Years) Value | Deaths Country | Deaths Value | Incidence Country | Incidence Value |
| 1 | Armenia | 0.05 (0.05, 1653.38) | Armenia | 0.05 (0.05, 58.38) | Armenia | 0.05 (0.05, 591.20) |
| 2 | Bulgaria | 0.05 (0.05, 54018.61) | Bulgaria | 0.05 (0.05, 2871.58) | Bulgaria | 0.05 (0.05, 16859.28) |
| 3 | Romania | 0.05 (0.05, 2897.19) | Romania | 0.05 (0.05, 148.65) | Romania | 0.05 (0.05, 842.34) |
| 4 | Guatemala | 0.05 (0.05, 4016.29) | Guyana | 0.05 (0.05, 564.05) | Guatemala | 0.05 (0.05, 1202.01) |
| 5 | Bahrain | 0.05 (0.05, 12522.80) | Guatemala | 0.05 (0.05, 226.53) | Bahrain | 0.05 (0.05, 3395.84) |
| 6 | Qatar | 0.05 (0.05, 7354.76) | Bahrain | 0.05 (0.05, 863.09) | Qatar | 0.05 (0.05, 1791.62) |
| 7 | Greece | 634.25 (0.05, 3193.57) | Qatar | 0.05 (0.05, 447.36) | Estonia | 78.23 (0.05, 368.61) |
| 8 | Estonia | 697.31 (0.05, 1711.66) | Ukraine | 4.05 (0.05, 122.77) | Greece | 153.71 (0.05, 856.80) |
| 9 | Republic of Moldova | 1131.53 (0.05, 4029.35) | Poland | 29.66 (0.05, 133.42) | Russian Federation | 245.33 (0.05, 800.20) |
| 10 | Russian Federation | 1208.33 (0.05, 3080.76) | Greece | 35.41 (0.05, 182.27) | Republic of Moldova | 266.82 (0.05, 1076.22) |
